# Minimally invasive surgical strategies for gallbladder stones with concomitant common bile duct stones: a systematic review and network meta-analysis

**DOI:** 10.1186/s12893-026-04003-x

**Published:** 2026-07-03

**Authors:** Ziwen Liao, Xueyuan Zhu, Yahui Wang, Jiao’an Pang, Jun Fu, Jingxuan Wei, Yuan Yu

**Affiliations:** 1 Department of Hepatobiliary Surgery, The First Affiliated Hospital of Guangxi University of Chinese Medicine, Nanning, 530023 China; 2 Guangxi University of Chinese Medicine, Nanning, 530200 China

**Keywords:** Choledocholithiasis, Gallstones, Laparoscopic common bile duct exploration, ERCP, Network meta-analysis

## Abstract

**Background:**

Approximately 10–20% of patients with gallstones present with concomitant common bile duct stones (CBDS). Although T-tube drainage (TTD) has traditionally been used after bile duct exploration, clinical practice is increasingly shifting toward T-tube-free minimally invasive strategies to enhance postoperative recovery. This study evaluated the effectiveness and safety of these approaches using a network meta-analysis (NMA).

**Methods:**

PubMed, Web of Science, Embase, and the Cochrane Library were searched from inception to October 2025. Randomized controlled trials (RCTs) and cohort studies were analyzed in parallel using separate frequentist NMAs. Interventions included laparoscopic cholecystectomy (LC) + laparoscopic common bile duct exploration (LCBDE) + TTD, LC + LCBDE + primary suture (PS), LC + laparoscopic transcystic common bile duct exploration (LTCBDE), and LC + endoscopic retrograde cholangiopancreatography (ERCP) performed as either a single-stage or two-stage strategy. Treatment effects were expressed as risk ratios (RRs), odds ratios (ORs), and mean differences (MDs), with treatment rankings estimated using the surface under the cumulative ranking curve (SUCRA).

**Results:**

Fifty-two studies including 11,327 patients (19 RCTs and 33 cohort studies) were included. Parallel analyses demonstrated generally consistent findings between randomized and observational evidence. In the RCT network, T-tube-free strategies achieved comparable stone clearance and overall safety compared with TTD. LC + LTCBDE showed the highest SUCRA ranking probabilities for operative time (MD − 47.01 min; SUCRA 96.4%) and length of hospital stay (MD − 5.78 days; SUCRA 86.9%). LC + LCBDE + PS was also associated with shorter operative time (MD − 20.02 min; SUCRA 51.8%) and shorter length of hospital stay (MD − 3.03 days; SUCRA 33.8%). Long-term outcomes and postoperative complication rates were generally comparable across strategies. Differences in SUCRA ranking probabilities were observed for postoperative pancreatitis-related outcomes, with LC + ERCP (two-stage) showing a lower ranking probability (SUCRA 15.8%).

**Conclusions:**

T-tube-free minimally invasive strategies achieved stone clearance and overall perioperative safety broadly comparable to those of TTD in patients with gallbladder stones and concomitant common bile duct stones. LC + LTCBDE and LC + LCBDE + PS were associated with shorter operative time and hospital stay in some network comparisons. Overall, treatment selection should remain individualized according to biliary anatomy, stone burden, patient condition, and institutional expertise.

**Supplementary Information:**

The online version contains supplementary material available at https://doi.org/10.1186/s12893-026-04003-x.

## Introduction

Gallstones are among the most common biliary tract diseases worldwide, and approximately 10–20% of patients with gallstones also present with concomitant common bile duct stones (CBDS) [1]. If not treated appropriately and promptly, CBDS may further progress to serious complications, including acute cholangitis, obstructive jaundice, and acute biliary pancreatitis [2–3]. With the increasing adoption of enhanced recovery after surgery (ERAS) principles, the goals of biliary surgery have gradually evolved beyond simple stone removal toward minimizing surgical trauma and optimizing perioperative recovery [4]. Therefore, under the premise of maintaining effective stone clearance, selecting an appropriate minimally invasive strategy for patients with CBDS has become an important issue in contemporary biliary surgery.

Traditionally, laparoscopic common bile duct exploration (LCBDE) combined with T-tube drainage (TTD) has been regarded as a standard approach for biliary decompression and postoperative management [1]. However, with accumulating clinical experience, several limitations associated with T-tube placement have become increasingly recognized, including bile leakage, electrolyte imbalance, accidental tube dislodgement, and reduced postoperative quality of life [5]. In recent years, advances in minimally invasive and endoscopic techniques have led to growing interest in T-tube-free strategies, which have gradually been adopted in experienced centers. Current guidelines and related studies recognize laparoscopic transcystic bile duct exploration (LTCBDE) as a feasible minimally invasive option in selected patients with favorable biliary anatomy, limited stone burden, and an appropriate common bile duct diameter [6]. However, validated thresholds for common bile duct diameter, stone burden, and biliary anatomical suitability for LTCBDE remain inconsistent or not well established across current guidelines and studies.

To avoid T-tube-related complications, several T-tube-free minimally invasive strategies have been increasingly adopted in clinical practice. Primary suture (PS) after LCBDE not only avoids prolonged external drainage but may also help maintain physiological bile flow into the intestine [7–8]. LTCBDE utilizes the cystic duct as an access route for bile duct exploration and may avoid choledochotomy in selected patients with favorable biliary anatomy [9]. In addition, laparoscopic cholecystectomy (LC) combined with endoscopic retrograde cholangiopancreatography (ERCP) represents another commonly used strategy and may be performed either as a single-stage procedure, such as the intraoperative rendezvous technique, or as a staged two-step approach according to the clinical scenario [10]. However, current evidence regarding different T-tube-free strategies is still largely derived from direct comparisons between selected procedures, and conclusions remain inconsistent because of limited sample sizes and heterogeneity in baseline patient characteristics [11–12]. In particular, comprehensive comparative evidence regarding the overall efficacy and safety of multiple T-tube-free strategies remains limited.

Network meta-analysis (NMA) enables the integration of both direct and indirect evidence within a unified statistical framework, allowing simultaneous comparisons across multiple interventions. Therefore, the present study conducted a systematic review and NMA to comprehensively compare commonly used T-tube-free minimally invasive strategies, including PS, LTCBDE, and single-stage or two-stage LC + ERCP, with conventional LC + LCBDE + TTD. Separate networks were constructed for RCTs and observational cohort studies to allow parallel evaluation of findings across different study designs and to reduce potential bias arising from combining heterogeneous evidence sources. By comprehensively evaluating single-session stone clearance, perioperative recovery, and major postoperative complications, this study aimed to provide comparative evidence to support individualized minimally invasive treatment decision-making for patients with CBDS while acknowledging potential variability in patient selection and procedural indications across strategies.

## Methods

### Study design and registration

This systematic review and network meta-analysis was prospectively registered in the International Prospective Register of Systematic Reviews (PROSPERO; registration number CRD420251253169). The study was conducted and reported in accordance with the Preferred Reporting Items for Systematic Reviews and Meta-Analyses (PRISMA) 2020 statement and its extension for network meta-analyses (PRISMA-NMA) [13–14].

### Search strategy

A comprehensive literature search was performed in PubMed, Web of Science, Embase, and the Cochrane Central Register of Controlled Trials (CENTRAL) from database inception to October 2025. The search aimed to identify studies evaluating minimally invasive treatment strategies for gallbladder stones with concomitant CBDS. Medical Subject Headings (MeSH) terms and free-text keywords related to gallstone disease, CBDS, laparoscopic cholecystectomy, laparoscopic common bile duct exploration, T-tube drainage, primary suture, and endoscopic retrograde cholangiopancreatography were combined using appropriate Boolean operators (“AND” and “OR”). The detailed search strategies for each database are provided in Appendix 1.

### Eligibility and exclusion criteria

RCTs and high-quality cohort studies enrolling adult patients (≥ 18 years) diagnosed with gallbladder stones concomitant with CBDS were eligible for inclusion. Interventions included T-tube-free minimally invasive strategies, such as LC + LCBDE + PS, LTCBDE, and LC + ERCP. LC + ERCP was classified as a single-stage strategy when both procedures were performed under the same anesthesia, and as a staged (two-stage) strategy when performed sequentially. Comparators included LC + LCBDE + TTD or any of the aforementioned strategies. Because treatment allocation in real-world clinical practice is influenced by anatomical characteristics, stone burden, and patient condition, potential differences in baseline characteristics across interventions were anticipated.

Exclusion criteria consisted of conference abstracts, reviews, case reports, animal studies, and non-comparative studies. Patients with malignant biliary or pancreatic diseases, congenital biliary anomalies, or exclusively pediatric populations were also excluded. When LCBDE involved mixed techniques (e.g., PS, TTD, or LTCBDE) and outcome data could not be reliably disaggregated, such studies were retained as a “mixed” LCBDE node in the main analysis to accurately reflect real-world clinical reporting limitations. Their influence was further explored through prespecified sensitivity analyses.

### Screening process

All retrieved records were imported into EndNote version 20.4.1 (Clarivate Analytics, Philadelphia, PA, USA), and duplicate records were removed. Study selection was conducted in three stages by three independent reviewers (Z.L., X.Z., and Y.W.). First, titles were screened for relevance, and potentially eligible records were retained. Second, abstracts of selected studies were reviewed, with disagreements resolved through discussion. Third, full texts of eligible studies were assessed against the predefined inclusion and exclusion criteria. Any unresolved discrepancies were adjudicated by a fourth reviewer (Y.Y.).

### Data extraction and outcome measures

Data extraction was independently performed by two reviewers using a standardized, pre-designed data collection form. Discrepancies were resolved through discussion or consultation with a third reviewer when necessary. Extracted data included first author, publication year, country, study design, total sample size, intervention strategies, and sample size for each treatment group. Baseline characteristics, when reported, included mean age, sex distribution, stone size, number of stones, common bile duct diameter, and duration of follow-up.

The primary outcomes were single-session stone clearance rate and severe postoperative complications, defined as Clavien-Dindo grade III or higher events or complications requiring reoperation, intensive care unit admission, or resulting in disability or death. Secondary outcomes included perioperative outcomes (operative time, intraoperative blood loss, total length of hospital stay, conversion to open surgery, and reintervention), postoperative complications (bile leakage, pancreatitis, biliary bleeding, surgical site infection, intra-abdominal infection, and cholangitis), and long-term outcomes (symptomatic biliary stricture and stone recurrence, confirmed by imaging or ERCP). Definitions of postoperative complications were extracted according to the criteria reported in the original studies or established international definitions when available. When available, information regarding complication severity was extracted.

### Quality assessment

The risk of bias of RCTs was assessed using the Cochrane Risk of Bias tool (RoB 2.0). The methodological quality of cohort studies was evaluated using the Newcastle-Ottawa Scale (NOS), which assesses participant selection, comparability of study groups, and outcome assessment, with a maximum score of nine stars indicating higher quality. Small-study effects were explored using funnel plots and Egger’s test when appropriate [15]. The certainty of evidence was further evaluated using the Confidence in Network Meta-Analysis (CINeMA) framework. Assessment of transitivity considered potential effect modifiers, including baseline age, stone size, and common bile duct diameter, across studies contributing direct and indirect evidence [16, 17].

### Data synthesis and statistical analysis

For continuous outcomes not reported as mean ± standard deviation (SD), the mean ± SD were estimated from the median and corresponding distribution range or interquartile range using established methods [18, 19]. In articles describing an LC + ERCP (two-stage) approach, data from “pre-ERCP sequential LC” and “post-LC postoperative ERCP” were consistently categorized under the “LC + ERCP (two-stage)” group to maintain network connectivity [20].

To enhance methodological rigor and reduce potential bias arising from differences in study design, randomized and observational evidence were synthesized in parallel but separate treatment networks within a frequentist framework using Stata version 18.0 (StataCorp LLC, College Station, TX, USA). Network plots were generated to illustrate the geometry of available direct comparisons. Node size was proportional to the total sample size of each intervention, and edge thickness represented the number of studies contributing to each comparison. Clinical transitivity was descriptively evaluated by comparing the distribution of major effect modifiers, including stone size, common bile duct diameter, and patient age, across treatment comparisons. Relative treatment effects were expressed as risk ratios (RRs) for outcomes with relatively common events, including single-session stone clearance, and as odds ratios (ORs) for other dichotomous outcomes, such as severe postoperative complications and reintervention. Continuous outcomes were summarized as mean differences (MDs) with corresponding 95% confidence intervals (CIs). Random-effects network meta-analyses using the restricted maximum likelihood (REML) method were applied to account for between-study heterogeneity [21].

Between-study heterogeneity was assessed using the variance parameter τ², with values interpreted as low (< 0.04), low-to-moderate (0.04–0.16), moderate-to-high (0.16–0.36), and high (> 0.36), according to previously published criteria [22–24]. A common heterogeneity variance was assumed across all treatment comparisons owing to limited data for several pairwise comparisons. Network inconsistency was evaluated using node-splitting models comparing direct and indirect evidence and a design-by-treatment interaction model assessing inconsistency across the entire network [25]. Treatments were ranked using the SUCRA [26], which was interpreted as a probabilistic ranking measure rather than direct evidence of clinically meaningful superiority.

### Sensitivity analyses

Sensitivity analyses were conducted by excluding studies with mixed intervention techniques from the network meta-analysis to evaluate the robustness of the findings. Because exclusion of these studies would substantially reduce network connectivity, they were retained as a separate mixed-intervention node in the primary analysis to reflect real-world reporting practices. Additional sensitivity analyses were performed to assess the stability of treatment estimates and SUCRA ranking probabilities under different assumptions regarding intervention classification.

## Results

### Literature selection and study characteristics

**Fig. 1:**
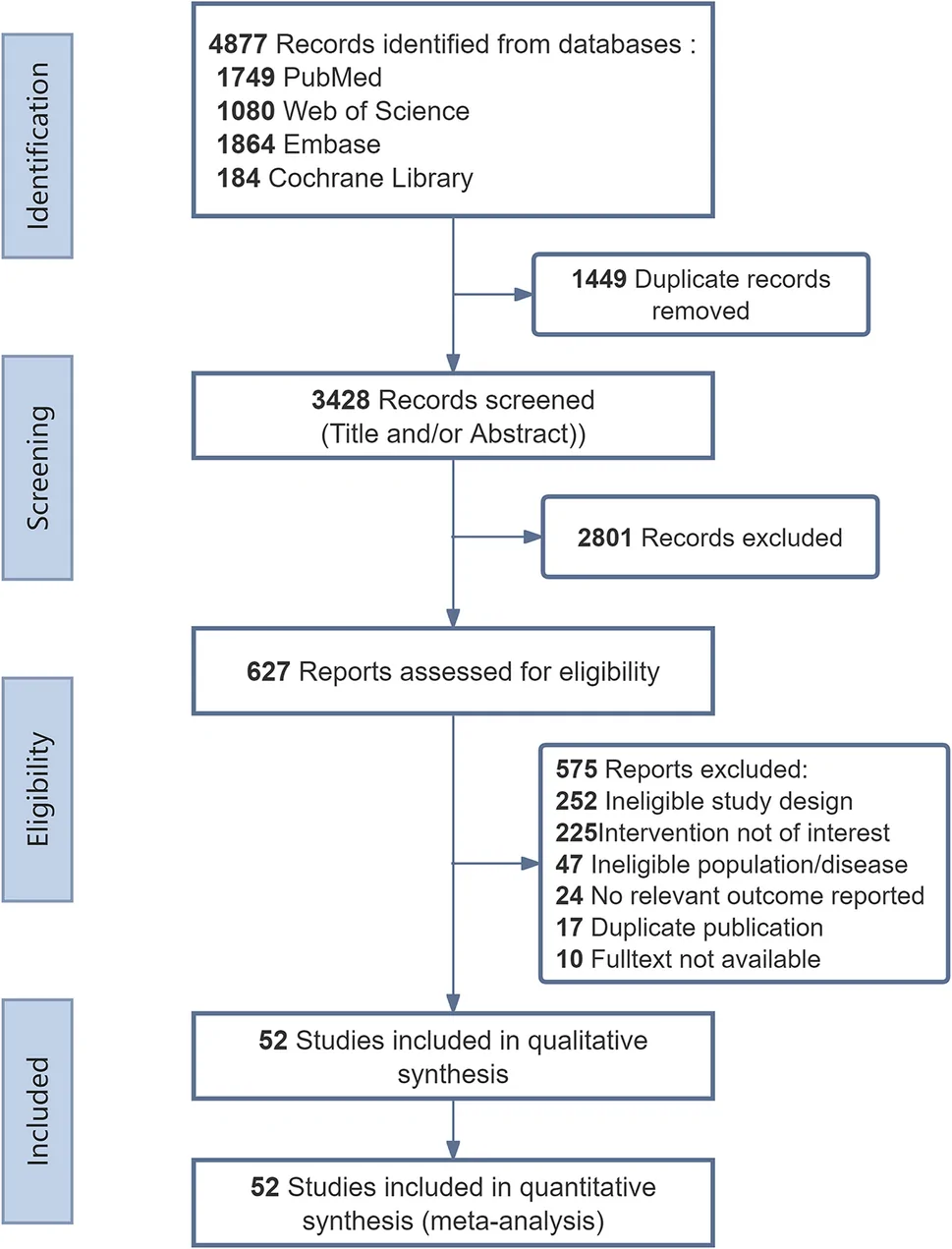
PRISMA 2020 flow diagram of study selection

A total of 4,877 records were identified through systematic searches of electronic databases. After the removal of 1,449 duplicate records, 3,428 unique records were screened based on titles and abstracts, of which 2,801 were excluded. Subsequently, 627 full-text articles were assessed for eligibility, and 427 studies were excluded for predefined reasons. Ultimately, 52 studies met the inclusion criteria, enrolling a total of 11,327 participants, and were included in both the qualitative synthesis and quantitative network meta-analysis (Fig. 1). Of the 52 included studies, 19 were randomized controlled trials (RCTs) [27–45] and 33 were retrospective cohort studies [46–78]. The included studies were conducted across multiple geographic regions, including Asia, Europe, Africa, and North America. Sample sizes ranged from 30 to 1,005 participants, with follow-up durations varying from 1 to 120 months.

Baseline characteristics of the included studies are summarized in Appendix 2 (Table S2). Participant age varied substantially across studies. Where reported, mean stone size ranged from approximately 0.40 cm to 2.83 cm, and mean common bile duct diameter ranged from approximately 0.63 cm to 1.87 cm. Several studies did not report specific baseline variables, such as stone size or bile duct diameter; these data were recorded as not available in Table S2. All eligible interventions were successfully mapped to the predefined treatment nodes according to the study protocol, resulting in connected treatment networks. The distribution of key baseline characteristics across intervention groups appeared broadly comparable; however, some variability in stone size, common bile duct diameter, and clinical selection patterns remained across studies.

### Risk of bias, consistency, and certainty of evidence

The risk of bias and methodological quality assessments are presented in the Supplementary Digital Content (Appendix 4). Among the 19 RCTs, most domains were judged as having a low risk of bias, with some concerns primarily related to insufficient reporting of blinding procedures and selective outcome reporting. No RCT was assessed as having a high risk of bias (Figure S4 and Table S4.1). For the 33 cohort studies, Newcastle-Ottawa Scale scores ranged from 6 to 9 stars, indicating moderate to high methodological quality (Table S4.2).

Assessment of heterogeneity and consistency across the network is summarized in Appendix 5. Between-study heterogeneity, assessed using the variance parameter τ², was low for most outcomes, although relatively higher heterogeneity was observed for operative time (τ² = 0.830) and intraoperative blood loss (τ² = 0.750). These findings suggest the presence of clinical heterogeneity across studies. No statistically significant global inconsistency was detected for any outcome, and no major disagreement between direct and indirect evidence was identified across the network (Tables S5.1a and S5.1b). The certainty of evidence assessed using the CINeMA framework ranged from low to moderate across most network estimates (Appendix 9).

Assessment of transitivity suggested that baseline characteristics across intervention groups were broadly comparable overall; however, clinically relevant variability remained across several treatment nodes. In particular, differences in mean age, stone size, and common bile duct diameter were observed between some intervention groups, with TTD-based strategies generally involving patients with relatively larger bile duct diameters and greater stone burden compared with strategies involving LTCBDE or ERCP. In addition, important effect modifiers, particularly stone size and common bile duct diameter, were incompletely reported across a substantial proportion of included studies, with several studies reporting these variables as unavailable. This extent of missingness limited the feasibility of robust network meta-regression analyses (Appendix 9, Tables S9.1–S9.2 and Figures S9.5–S9.6). Visual inspection of funnel plots did not suggest substantial small-study effects (Appendix 10).

### Primary outcomes

#### Single-session stone clearance rate

**Fig. 2:**
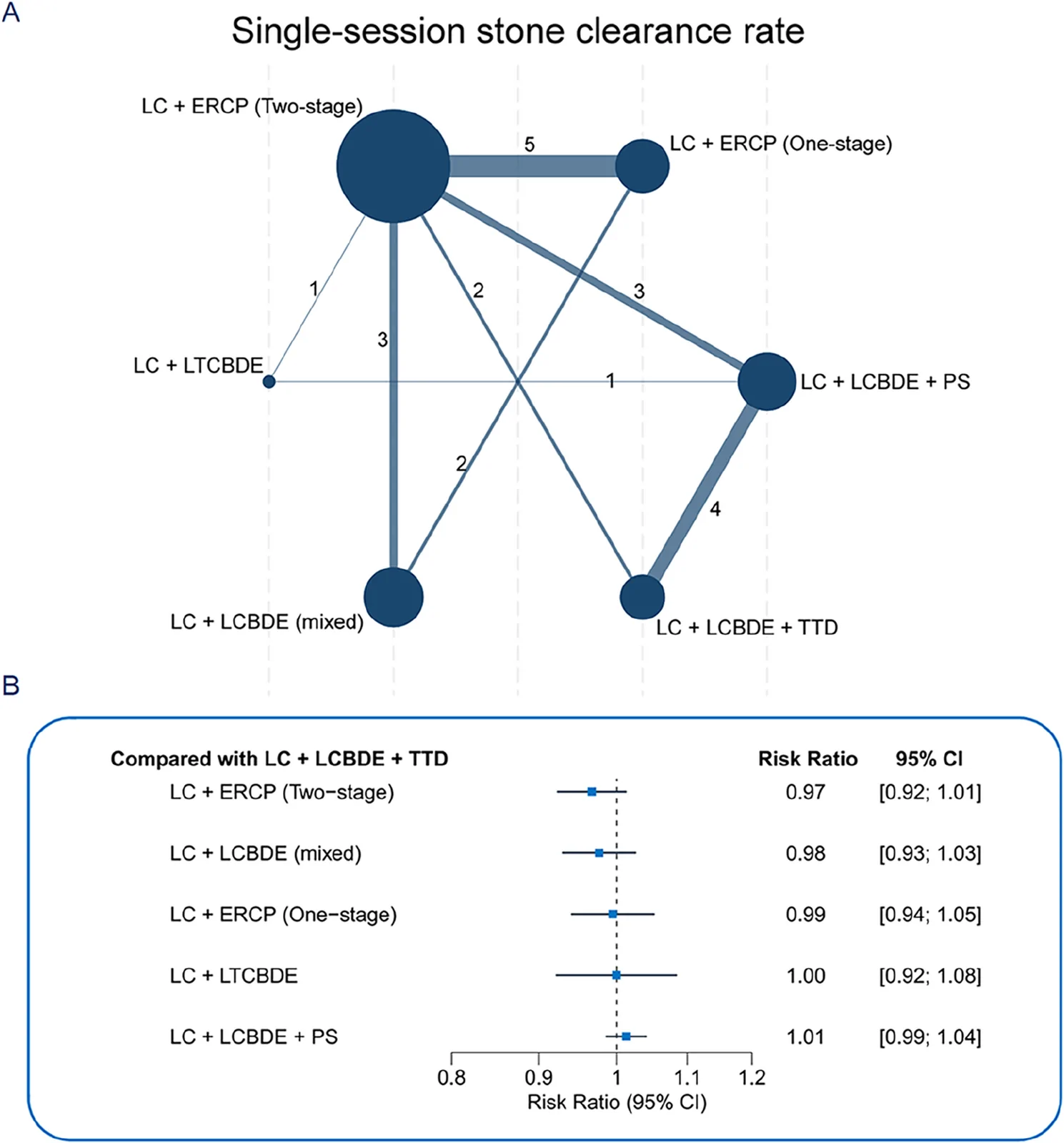
Network meta-analysis of single-session stone clearance rate. **A** Network geometry of minimally invasive strategies. Node size is proportional to the total number of participants included for each intervention, and line thickness reflects the number of direct comparisons between strategies. **B** Forest plot of RRs and 95% CIs for single-session stone clearance rate, using LC + LCBDE + TTD as the reference treatment. Abbreviations: LC, laparoscopic cholecystectomy; LCBDE, laparoscopic common bile duct exploration; LTCBDE, laparoscopic transcystic bile duct exploration; ERCP, endoscopic retrograde cholangiopancreatography; PS, primary suture; TTD, T-tube drainage

A total of 19 RCTs involving 2,723 patients reported single-session stone clearance rates. Overall, no statistically significant differences were observed between any T-tube-free strategies and the reference LC + LCBDE + TTD group (Fig. 2). SUCRA rankings suggested variation in ranking probabilities across interventions. LC + LCBDE + PS showed the highest SUCRA ranking probability for single-session stone clearance (82.4%), followed by LC + ERCP (single-stage) (59.6%), whereas LC + ERCP (two-stage) showed the lowest SUCRA ranking probability within the network (9.9%). Parallel network meta-analysis of cohort studies demonstrated generally consistent findings, with comparable clearance rates observed across interventions (Appendix Figure S6.1, Figure S7.1, Tables S7.1, Tables S8.1).

#### Severe postoperative complications

**Fig. 3:**
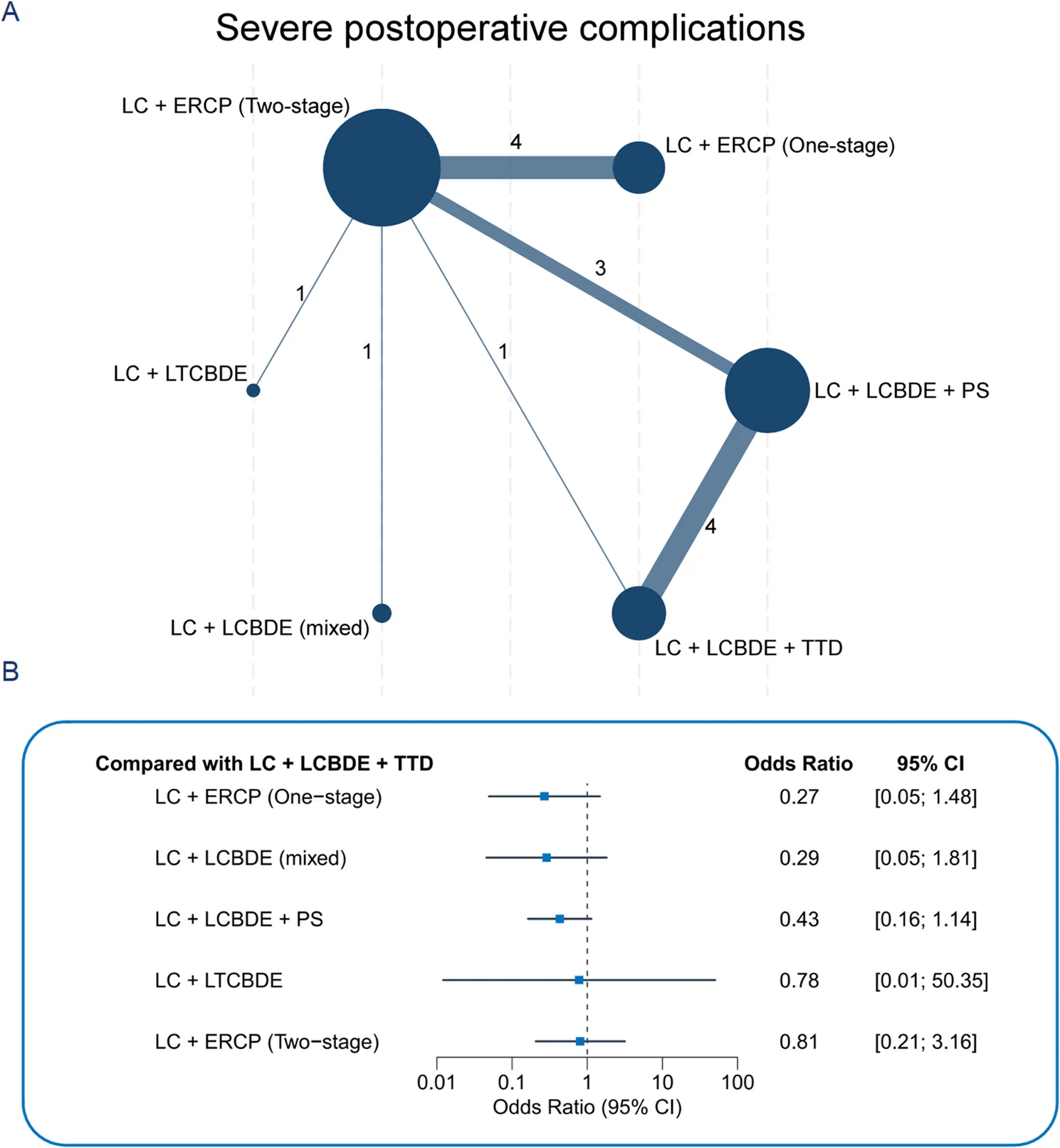
Network meta-analysis of severe postoperative complications. **A** Network geometry of treatment comparisons. Node size is proportional to the total number of participants included for each intervention, and line thickness reflects the number of direct comparisons between strategies. **B** Forest plot of ORs and 95% CIs for severe postoperative complications, using LC + LCBDE + TTD as the reference treatment. Abbreviations: LC, laparoscopic cholecystectomy; LCBDE, laparoscopic common bile duct exploration; LTCBDE, laparoscopic transcystic bile duct exploration; ERCP, endoscopic retrograde cholangiopancreatography; PS, primary suture; TTD, T-tube drainage

Data from 14 RCTs (2,022 patients) were analyzed for severe postoperative complications. Because of the low event rate, no statistically significant differences were detected across the network (Fig. 3). SUCRA rankings suggested variation in ranking probabilities across interventions. LC + ERCP (single-stage) showed the highest SUCRA ranking probability (77.3%), followed by LC + LCBDE + PS (60.5%) and LTCBDE (41.8%), whereas LC + LCBDE + TTD showed the lowest SUCRA ranking probability (20.9%). Parallel network meta-analysis of cohort studies demonstrated generally consistent findings, with no statistically significant differences observed across interventions (Appendix Figure S6.2, Figure S7.2, Tables S8.2).

### Secondary outcomes

#### Perioperative outcomes

##### Operative time

**Fig. 4:**
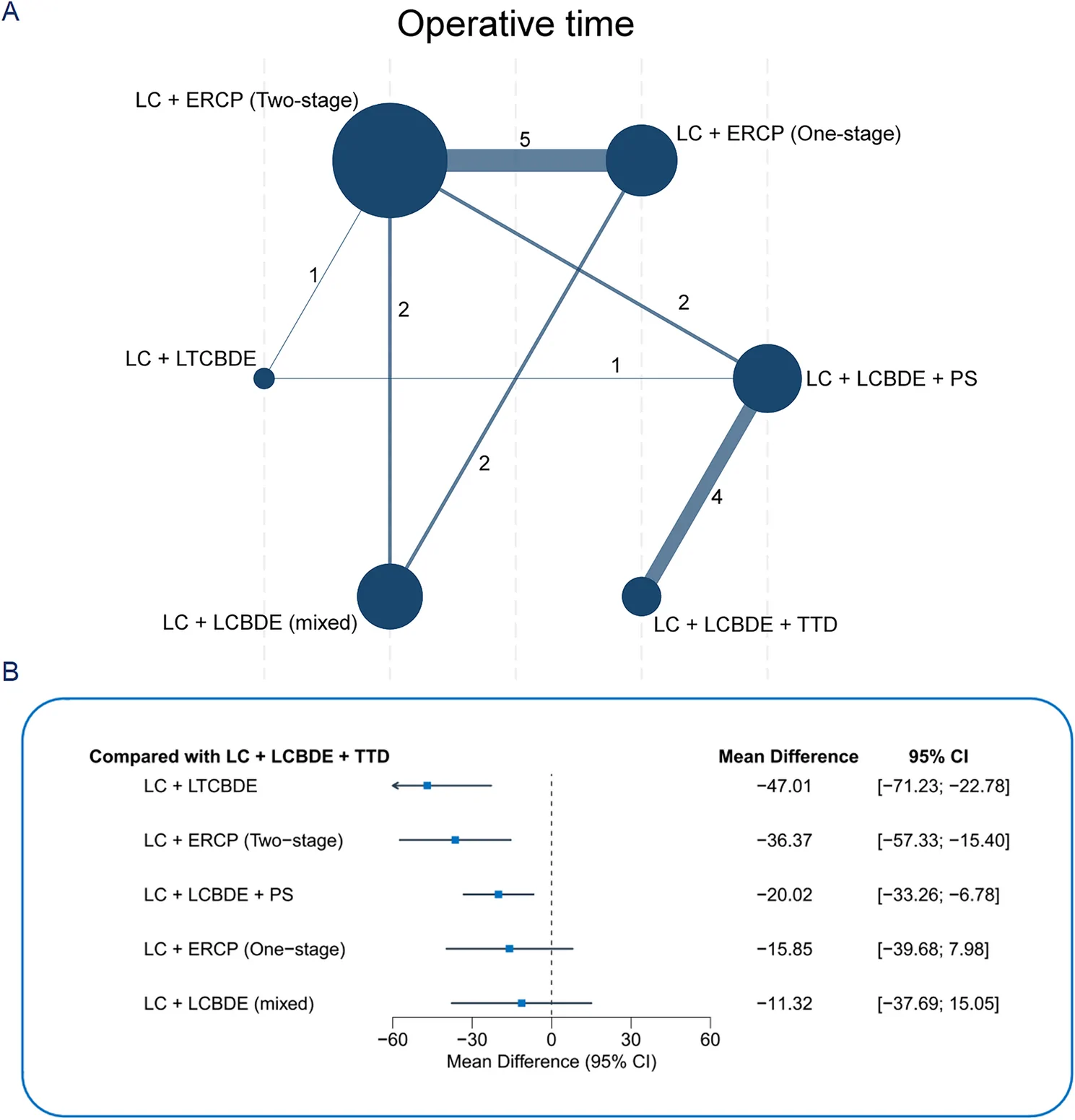
Network meta-analysis of operative time. **A** Network geometry of treatment comparisons. Node size is proportional to the total number of participants included for each intervention, and line thickness reflects the number of direct comparisons between strategies. **B** Forest plot of MDs and 95% CIs for operative time, using LC + LCBDE + TTD as the reference treatment. Negative values indicate shorter operative time compared with the reference. Abbreviations: LC, laparoscopic cholecystectomy; LCBDE, laparoscopic common bile duct exploration; LTCBDE, laparoscopic transcystic bile duct exploration; ERCP, endoscopic retrograde cholangiopancreatography; PS, primary suture; TTD, T-tube drainage

Nineteen RCTs reported operative time. Network meta-analysis showed that, compared with LC + LCBDE + TTD, multiple T-tube-free strategies were associated with significantly shorter operative times. Among these, LTCBDE showed the highest SUCRA ranking probability for operative time (MD − 47.01 min, 95% CI − 71.23 to − 22.78; SUCRA 96.4%). LC + ERCP (two-stage) (MD − 36.37 min, 95% CI − 57.33 to − 15.40; SUCRA 82.8%) and LC + LCBDE + PS (MD − 20.02 min, 95% CI − 33.26 to − 6.78; SUCRA 51.8%) were also associated with significantly shorter operative times compared with LC + LCBDE + TTD (Fig. 4). Parallel network meta-analysis of cohort studies demonstrated generally consistent findings, showing that T-tube-free strategies were also associated with shorter operative times compared with LC + LCBDE + TTD (LTCBDE, SUCRA 96.4%; PS, SUCRA 79.5%) (Appendix Figures S6.3, Tables S7.3, Tables S8.3).

##### Total length of hospital stay

**Fig. 5:**
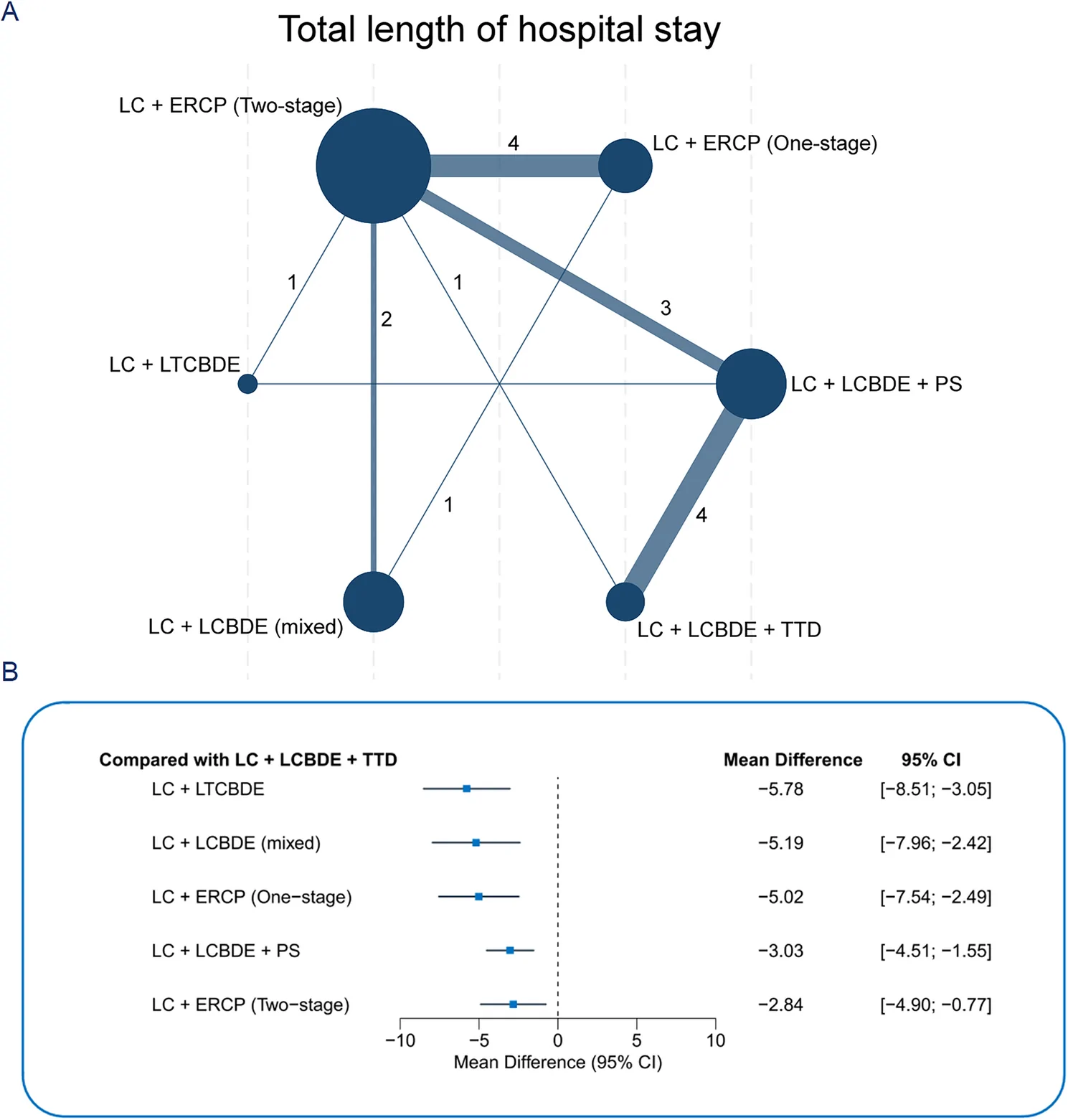
Network meta-analysis of total length of hospital stay. **A** Network geometry of treatment comparisons. Node size is proportional to the total number of participants included for each intervention, and line thickness reflects the number of direct comparisons between strategies. **B** Forest plot of MDs and 95% CIs for total length of hospital stay, using LC + LCBDE + TTD as the reference treatment. Negative values indicate shorter hospital stay compared with the reference. Abbreviations: LC, laparoscopic cholecystectomy; LCBDE, laparoscopic common bile duct exploration; LTCBDE, laparoscopic transcystic bile duct exploration; ERCP, endoscopic retrograde cholangiopancreatography; PS, primary suture; TTD, T-tube drainage

Eighteen RCTs were included in the network meta-analysis of total length of hospital stay. All T-tube-free strategies were associated with significantly shorter hospital stays compared with LC + LCBDE + TTD. LTCBDE showed the highest SUCRA ranking probability for shorter hospital stay (MD − 5.78 days, 95% CI − 8.51 to − 3.05; SUCRA 86.9%), followed by the mixed-intervention LC + LCBDE node (MD − 5.19 days, 95% CI − 7.96 to − 2.42; SUCRA 77.4%), LC + ERCP (single-stage) (MD − 5.02 days, 95% CI − 7.54 to − 2.49; SUCRA 73.8%), LC + LCBDE + PS (MD − 3.03 days, 95% CI − 4.51 to − 1.55; SUCRA 33.8%), and LC + ERCP (two-stage) (MD − 2.84 days, 95% CI − 4.90 to − 0.77; SUCRA 28.0%) (Fig. 5). Parallel network meta-analysis of cohort studies demonstrated generally consistent findings, showing that LTCBDE (MD − 3.78 days, 95% CI − 6.27 to − 1.29; SUCRA 86.9%) and LC + LCBDE + PS (MD − 2.36 days, 95% CI − 4.04 to − 0.68; SUCRA 62.6%) were associated with significantly shorter hospital stays compared with LC + LCBDE + TTD (Appendix Figures S6.4, Tables S7.4, Tables S8.4).

##### Other perioperative outcomes

Because only a limited number of RCTs reported intraoperative blood loss, only cohort-based network meta-analysis results are presented for this outcome. LTCBDE showed the highest SUCRA ranking probability for lower intraoperative blood loss (MD − 32.45 mL, 95% CI − 56.74 to − 8.17; SUCRA 94.8%). No statistically significant differences were observed among strategies with respect to conversion to open surgery or reintervention. SUCRA rankings from RCT data suggested that LTCBDE (SUCRA 79.2%) and LC + LCBDE + PS (SUCRA 62.8%) had relatively higher ranking probabilities for conversion to open surgery outcomes. Similar SUCRA ranking patterns were observed for reintervention, with LTCBDE (SUCRA 81.5%) and LC + LCBDE + PS (SUCRA 68.3%) showing relatively higher ranking probabilities within the network. Parallel cohort-based analyses demonstrated generally consistent findings (Appendix Figures S6.5-S6.7, Tables S7.5-S7.7, Tables S8.5-S8.7).

##### Postoperative complications

Outcomes evaluated included bile leakage, postoperative pancreatitis, biliary bleeding, surgical-site infection, intra-abdominal infection, and cholangitis. Network meta-analysis revealed no statistically significant differences in complication rates between any T-tube-free strategy and LC + LCBDE + TTD. SUCRA rankings varied across interventions. For bile leakage, LC + ERCP (two-stage) (SUCRA 91.8%) and LTCBDE (SUCRA 85.7%) showed relatively higher ranking probabilities within the network. For postoperative pancreatitis, LC + LCBDE + TTD (SUCRA 85.2%) and LC + LCBDE + PS (SUCRA 77.8%) showed relatively higher ranking probabilities, whereas LC + ERCP (two-stage) showed a lower SUCRA ranking probability (15.8%). For biliary bleeding, the mixed-intervention LC + LCBDE node (SUCRA 78.5%) and LC + LCBDE + PS (SUCRA 76.7%) showed relatively higher ranking probabilities. For postoperative cholangitis, LTCBDE (SUCRA 71.0%) and LC + LCBDE + PS (SUCRA 57.4%) also showed relatively higher ranking probabilities within the network. Regarding intra-abdominal infection, LC + ERCP (single-stage) (SUCRA 62.6%) and LC + LCBDE + PS (SUCRA 51.3%) showed relatively higher ranking probabilities, whereas for surgical-site infection, LC + LCBDE + PS (SUCRA 57.6%) and LTCBDE (SUCRA 50.6%) showed similar ranking patterns. Parallel cohort-based analyses demonstrated generally consistent findings with the RCT-based analyses (Figures S6.8-S6.13, Tables S7.8-S7.13, Tables S8.8-S8.13).

##### Long-term outcomes

Regarding long-term outcomes, including symptomatic biliary stricture and stone recurrence, no statistically significant differences were detected among strategies. SUCRA rankings based on RCT data varied across interventions. For symptomatic biliary stricture, LC + ERCP (two-stage) (SUCRA 69.7%) and LC + ERCP (single-stage) (SUCRA 57.0%) showed relatively higher ranking probabilities within the network. For stone recurrence, LC + LCBDE + TTD (SUCRA 69.9%) and LC + ERCP (single-stage) (SUCRA 66.3%) also showed relatively higher ranking probabilities. Parallel cohort-based analyses demonstrated generally consistent findings, with no statistically significant differences observed across intervention groups (Appendix Figures S6.14-S6.15, Tables S7.14-S7.15, Tables S8.14-S8.15).

### Sensitivity analyses

Sensitivity analyses excluding the mixed-intervention node (LC + LCBDE [mixed]) yielded findings generally consistent with the primary analyses for both single-session stone clearance and severe postoperative complications, suggesting that the overall results were robust to different assumptions regarding intervention classification. Detailed results are provided in the Supplementary Content (Tables S11.2a-S11.2b).

## Discussion

### Principal findings

This network meta-analysis integrated data from 52 studies involving 11,327 patients to systematically evaluate multiple T-tube-free minimally invasive strategies for gallbladder stones with concomitant CBDS. The findings demonstrated that T-tube-free approaches achieved stone clearance rates and overall perioperative safety profiles that were broadly comparable to those of conventional LC + LCBDE + TTD, while being associated with shorter operative time and hospital stay in some network comparisons. These findings further support the growing emphasis in contemporary biliary surgery on T-tube-free minimally invasive management strategies [79]. Among the evaluated strategies, LC + LTCBDE showed relatively shorter operative time and hospital stay across several comparisons within the network. Similarly, LC + LCBDE + PS demonstrated acceptable postoperative recovery profiles while avoiding routine external biliary drainage. In contrast, LC + ERCP (two-stage) showed relatively lower SUCRA ranking probabilities for some perioperative recovery and postoperative pancreatitis-related outcomes, although no significant differences in overall complication rates were observed across the network. Overall, the present findings suggest that no single minimally invasive strategy is universally optimal for all patients with concomitant gallbladder and common bile duct stones. Rather, different approaches may offer potential advantages in specific clinical settings and should be selected according to individual patient characteristics and local expertise.

### Interpretation and clinical implications

The present study showed that different T-tube-free minimally invasive strategies achieved broadly comparable single-session stone clearance rates, suggesting that differences in biliary clearance efficacy across treatment pathways may be gradually narrowing with ongoing advances in laparoscopic bile duct exploration techniques, endoscopic equipment, and perioperative management [80–81]. In experienced centers, both surgical exploration and endoscopic intervention can generally achieve reliable bile duct stone clearance, which may partly explain the absence of statistically significant differences observed across interventions within the network. Nevertheless, some perioperative recovery outcomes appeared to differ across strategies. In particular, LC + LTCBDE showed relatively shorter operative time and length of hospital stay in some comparisons within the network. Previous studies have similarly suggested that transcystic exploration may facilitate perioperative recovery in selected patients by avoiding choledochotomy and reducing the need for direct bile duct incision [82]. Similarly, LC + LCBDE + PS demonstrated relatively shorter postoperative recovery outcomes, and prior evidence also indicates that avoiding routine T-tube placement and external biliary drainage may help facilitate postoperative recovery without substantially increasing the risk of bile leakage [83, 84]. Therefore, these observed perioperative differences should be interpreted cautiously.

However, the selection of minimally invasive treatment pathways in clinical practice is often influenced not only by procedural characteristics, but also by factors such as biliary anatomy, stone burden, overall patient condition, and institutional expertise [85]. Consequently, baseline differences between patient populations undergoing different interventions may exist. For example, LTCBDE is generally more suitable for patients with favorable biliary anatomy and relatively smaller stones [86–87], whereas ERCP-based approaches are more commonly used in elderly, high-risk, or anatomically complex cases [88]. In several included studies, patients undergoing LTCBDE also tended to present with smaller stones and more favorable biliary anatomy, which may partly explain the observed perioperative recovery differences. This patient-selection pattern may also reflect the lack of uniformly accepted international criteria for LTCBDE indications. Current guidelines generally recognize transcystic exploration as a feasible option for selected patients with relatively small stones and favorable biliary anatomy but do not define universally accepted quantitative thresholds [1, 6]. More recently, a 2025 expert consensus proposed specific selection criteria for LTCBDE, including a stone diameter < 1.5 cm and a common bile duct diameter ≥ 4 mm when complete transcystic clearance is considered achievable [89]. Nevertheless, these criteria remain partly experience-dependent and have not yet been universally validated internationally. Therefore, some indirect comparisons within the network may partly reflect differences in patient selection (confounding by indication) rather than intrinsic procedural superiority. Although potential effect modifiers, including stone size, common bile duct diameter, and patient age, were descriptively evaluated, inconsistent reporting and missing baseline data across studies limited the feasibility of robust network meta-regression analyses. As a result, perioperative recovery outcomes may still have been influenced by residual confounding.

Regarding safety outcomes, LC + ERCP (two-stage) showed relatively lower SUCRA ranking probabilities for postoperative pancreatitis-related outcomes, although no statistically significant differences in overall complication rates were consistently observed across the network. These findings may partly reflect differences in procedural complexity and clinical context associated with staged endoscopic treatment pathways rather than the intrinsic risk of the procedures themselves [85]. In addition, most cases of post-ERCP pancreatitis are generally mild and self-limiting [90]. Some previous studies have also suggested that single-stage strategies may be associated with fewer reinterventions and shorter hospital stay [91], although these observations may likewise be influenced by differences in patient selection and perioperative management across centers. For long-term outcomes, no statistically significant differences in symptomatic biliary stricture were observed among strategies. Although LC + ERCP (two-stage) showed a relatively higher SUCRA ranking probability, the limited number of events and variability in follow-up duration preclude any firm conclusions regarding long-term advantages.

In addition, operative time (τ² = 0.830) and intraoperative blood loss (τ² = 0.750) demonstrated relatively high statistical heterogeneity. In addition to differences in institutional expertise and surgeon experience, these outcomes may also have been influenced by operative factors such as the feasibility of transcystic exploration, the need for choledochotomy, and the management complexity of bile duct stones [92]. Furthermore, SUCRA rankings should be interpreted alongside absolute effect estimates, confidence intervals, and clinical context, because most ranking differences observed in the present network were exploratory and were not consistently supported by statistically significant pairwise differences.

### Strengths and limitations

This study systematically evaluated multiple T-tube-free minimally invasive strategies using a network meta-analysis framework. One methodological strength was the refined classification of treatment nodes, particularly the inclusion of LTCBDE as an independent intervention, which allowed a more specific assessment of the potential role of the transcystic approach in contemporary biliary surgery. In addition, separate networks were constructed for RCTs and cohort studies, enabling parallel evaluation of randomized evidence and real-world clinical data. The generally consistent trends observed across the two study designs increased the overall robustness of the findings. Prespecified sensitivity analyses further assessed the influence of the mixed-intervention node on network stability, while application of the CINeMA framework improved the systematic evaluation of evidence certainty.

Several limitations should also be acknowledged. First, the certainty of evidence for some outcomes remained limited by statistical imprecision and clinical heterogeneity, including differences in surgeon experience, perioperative management, and follow-up duration across studies. Second, reporting of important baseline characteristics, such as stone size, common bile duct diameter, and biliary anatomy, was inconsistent among the original studies. As a result, potential effect modifiers could only be assessed descriptively, and indirect comparisons may still have been influenced by baseline differences between patient populations. In addition, a small number of studies used mixed LCBDE techniques that could not be fully separated into specific intervention categories. Although a separate mixed node and sensitivity analyses were used to reduce its impact, this classification may still have introduced additional clinical heterogeneity. Definitions and severity grading of postoperative complications also varied across studies, particularly for post-ERCP pancreatitis, which may have affected the stability of safety-related findings. Finally, most included studies originated from Asian countries, and Chinese-language databases were not searched. Given that the implementation of LCBDE is partly dependent on institutional expertise and multidisciplinary collaboration, regional differences in technical adoption and clinical practice may limit the generalizability and individualized applicability of the present findings.

## Conclusion

The present study suggests that multiple T-tube-free minimally invasive strategies can achieve stone clearance and overall perioperative safety broadly comparable to those of conventional LC + LCBDE + TTD in the management of gallbladder stones with concomitant common bile duct stones. LC + LTCBDE and LC + LCBDE + PS were associated with shorter operative time and hospital stay in some network comparisons. Overall, different minimally invasive strategies have distinct characteristics, and treatment selection should remain individualized according to biliary anatomy, stone burden, overall patient condition, and institutional expertise. Further high-quality multicenter randomized controlled studies with standardized designs and longer follow-up are needed to better define the role of different minimally invasive strategies in long-term outcomes and specific patient populations. 

## Supplementary Information

Supplementary Material 1.

Supplementary Material 2.

## Data Availability

All data generated or analysed during this study are included in this published article and its Supplementary Appendix files.

## Abbreviations

CBDS: common bile duct stones
CBD: common bile duct
LC: laparoscopic cholecystectomy
LCBDE: laparoscopic common bile duct exploration
LTCBDE: laparoscopic transcystic bile duct exploration
TTD: T-tube drainage
PS: primary suture
ERCP: endoscopic retrograde cholangiopancreatography
ERAS: enhanced recovery after surgery
NMA: network meta-analysis
RCT: randomized controlled trial
NOS: Newcastle-Ottawa Scale
RoB 2.0: Risk of Bias 2.0 tool
CINeMA: Confidence in Network Meta-Analysis
SUCRA: surface under the cumulative ranking curve
REML: restricted maximum likelihood
RR: risk ratio
OR: odds ratio
MD: mean difference
CI: confidence interval
ICU: intensive care unit
PRISMA: Preferred Reporting Items for Systematic Reviews and Meta-Analyses
PRISMA-NMA: Preferred Reporting Items for Systematic Reviews and Meta-Analyses extension statement for network meta-analysis
CENTRAL: Cochrane Central Register of Controlled Trials
MeSH: Medical Subject Headings
SD: standard deviation
