# Supplementary material for: Minimally invasive surgical strategies for gallbladder stones with concomitant common bile duct stones: a systematic review and network meta-analysis
Source: BMC Surg. 2026 Jul 3;26:639. doi: 10.1186/s12893-026-04003-x (PMC13602641; doi:10.1186/s12893-026-04003-x)
Supplement: Supplementary file 2 [file 12893_2026_4003_MOESM2_ESM.docx]

**Supplementary Appendix**

**Minimally invasive surgical strategies for gallbladder stones with concomitant common bile duct stones: a network meta-analysis**

**Table of contents**

[Appendix 1: Search strategy	2](#_Toc25824)

[Appendix 2: Characteristics of included studies	6](#_Toc14405)

[Appendix 3: List of data extracted from the included studies	10](#_Toc26138)

[Appendix 4: Risk of bias and quality assessment of included studies	11](#_Toc31867)

[Appendix 5: Evaluation of inconsistency and heterogeneity	14](#_Toc20967)

[Appendix 6: Network maps and forest plots of outcomes	44](#_Toc24423)

[Appendix 7: SUCRA and cumulative probability plots	69](#_Toc18254)

[Appendix 8: League table of summary effect estimates	98](#_Toc29011)

[Appendix 9: CINeMA Assessment and Transitivity Assessment	113](#_Toc21834)

[Appendix 10: Funnel plots	122](#_Toc11711)

[Appendix 11: Sensitivity analyses	137](#_Toc22535)

# Appendix 1: Search strategy

**Table S1.1:** Search strategy of PubMed

| **#** | **Searches** |
| --- | --- |
| 1 | (((((((((((((((((Gallstones[MeSH Terms]) OR (Biliary Calculi[Title/Abstract])) OR (Calculi, Biliary[Title/Abstract])) OR (Gall Stone[Title/Abstract])) OR (Gall Stones[Title/Abstract])) OR (Gallstone[Title/Abstract])) OR (Common Bile Duct Calculi[Title/Abstract])) OR (Biliary Calculi, Common Bile Duct[Title/Abstract])) OR (Common Bile Duct Gall Stones[Title/Abstract])) OR (Common Bile Duct Gall Stone[Title/Abstract])) OR (Common Bile Duct Gallstones[Title/Abstract])) OR (Gall Stones, Common Bile Duct[Title/Abstract])) OR (Common Bile Duct Gallstone[Title/Abstract])) OR (Gallstones, Common Bile Duct[Title/Abstract])) OR (Cholelithiasis[Title/Abstract])) OR (Cholelithiases[Title/Abstract])) OR (Cholelithiases[Title/Abstract])) OR (Gallstone Diseases[Title/Abstract]) |
| 2 | (((((((Cholecystectomy, Laparoscopic[MeSH Terms]) OR (Cholecystectomy, Celioscopic[Title/Abstract])) OR (Celioscopic Cholecystectomies[Title/Abstract])) OR (Celioscopic Cholecystectomy[Title/Abstract])) OR Cholecystectomies, Celioscopic[Title/Abstract])) OR (Laparoscopic Cholecystectomy[Title/Abstract])) OR (Cholecystectomies, Laparoscopic[Title/Abstract])) OR (Laparoscopic Cholecystectomies[Title/Abstract]) |
| 3 | ((((((Choledochostomy[MeSH Terms]) OR (Choledochostomies[Title/Abstract])) OR (Laparoscopic Common Bile Duct Exploration[Title/Abstract])) OR (Laparoscopic Bile Duct Exploration[Title/Abstract])) OR (Laparoscopic Choledochoscopy[Title/Abstract])) OR (LCBDE[Title/Abstract])) OR (Common Bile Duct Exploration[Title/Abstract]) |
| 4 | ((((primary suture[Title/Abstract]) OR (primary closure[Title/Abstract])) OR (primary sutur[Title/Abstract])) OR (Primary Suturing[Title/Abstract])) OR (Suture Techniques[Title/Abstract]) |
| 5 | (((((((((((((((((((((((Cholangiopancreatography, Endoscopic Retrograde[MeSH Terms]) OR (Sphincterotomy, Endoscopic[MeSH Terms])) OR (ERCP[Title/Abstract])) OR (Retrograde Cholangiopancreatography, Endoscopic[Title/Abstract])) OR (Cholangiopancreatographies, Endoscopic Retrograde[Title/Abstract])) OR (Endoscopic Retrograde Cholangiopancreatographies[Title/Abstract])) OR (Endoscopic Retrograde Cholangiopancreatographies[Title/Abstract])) OR (Endoscopic Retrograde Cholangiopancreatography[Title/Abstract])) OR (EST[Title/Abstract])) OR (Endoscopic Biliary Sphincterotomy[Title/Abstract])) OR (Biliary Sphincterotomies, Endoscopic[Title/Abstract])) OR (Biliary Sphincterotomies, Endoscopic[Title/Abstract])) OR (Endoscopic Biliary Sphincterotomies[Title/Abstract])) OR (Sphincterotomies, Endoscopic Biliary[Title/Abstract])) OR (Sphincterotomy, Endoscopic Biliary[Title/Abstract])) OR (Papillotomy, Endoscopic[Title/Abstract])) OR (Endoscopic Papillotomies[Title/Abstract])) OR (Papillotomies, Endoscopic[Title/Abstract])) OR (Endoscopic Papillotomy[Title/Abstract])) OR (Endoscopic Sphincterotomy[Title/Abstract])) OR (Endoscopic Sphincterotomies[Title/Abstract])) OR (Sphincterotomies, Endoscopic[Title/Abstract])) OR (endoscopic papillary balloon dilatation[Title/Abstract])) OR (EPBD[Title/Abstract]) |
| 6 | ((((((laparoscopic transcystic common bile duct exploration[Title/Abstract]) OR (Laparoscopic Common Bile Duct Exploration[Title/Abstract])) OR (Laparoscopic Bile Duct Exploration[Title/Abstract])) OR (LTCBDE[Title/Abstract])) OR (Laparoscopic Choledochoscopy[Title/Abstract])) OR (via cystic duct[Title/Abstract])) OR (transcystic[Title/Abstract]) |
| 7 | #3 OR #4 OR #5 OR # 6 |
| 8 | #1 And #2 And #7 |
| 9 | ((((Meta-Analysis[MeSH Terms]) OR (Meta-Analysis as topic[MeSH Terms])) OR (Meta-Analysis[Publication Type])) OR (Review[Publication Type])) OR (Meta-Analysis[Title/Abstract]) |
| 10 | #8 NOT #9 |
| 11 | ((((((((((randomized controlled trial[Publication Type]) OR (controlled clinical trial[Publication Type])) OR (clinical trial[Publication Type])) OR (cohort studies[MeSH Terms])) OR (prospective studies[MeSH Terms])) OR (retrospective studies[MeSH Terms])) OR (comparative study[Publication Type])) OR (observational study[Publication Type])) OR (cohort[Title/Abstract])) OR (prospective[Title/Abstract])) OR (retrospective[Title/Abstract]) |
| 12 | #10 AND #11 |

**Table S1.2:** Search strategy of Web of Science: Science Citation Index Expanded

| **#** | **Searches** |
| --- | --- |
| 1 | (((((((((((((((((TS=(Gallstones)) OR TS=(Biliary Calculi)) OR TS=(Calculi, Biliary)) OR TS=(Gall Stone)) OR TS=(Gall Stones)) OR TS=(Gallstone)) OR TS=(Common Bile Duct Calculi)) OR TS=(Biliary Calculi, Common Bile Duct)) OR TS=(Common Bile Duct Gall Stones)) OR TS=(Common Bile Duct Gall Stone)) OR TS=(Common Bile Duct Gallstones)) OR TS=(Gall Stones, Common Bile Duct)) OR TS=(Common Bile Duct Gallstone)) OR TS=(Gallstones, Common Bile Duct)) OR TS=(Cholelithiasis)) OR TS=(Cholelithiases)) OR TS=(Gallstone Disease)) OR TS=(Gallstone Diseases) |
| 2 | (((((((TS=(Cholecystectomy, Laparoscopic)) OR TS=(Cholecystectomy, Celioscopic)) OR TS=(Celioscopic Cholecystectomies)) OR TS=(Celioscopic Cholecystectomy)) OR TS=(Cholecystectomies, Celioscopic)) OR TS=(Laparoscopic Cholecystectomy)) OR TS=(Cholecystectomies, Laparoscopic)) OR TS=(Laparoscopic Cholecystectomies) |
| 3 | ((((((TS=(Choledochostomy)) OR TS=(Choledochostomies)) OR TS=(Laparoscopic Common Bile Duct Exploration)) OR TS=(Laparoscopic Bile Duct Exploration)) OR TS=(Laparoscopic Choledochoscopy)) OR TS=(LCBDE)) OR TS=(Common Bile Duct Exploration) |
| 4 | ((((TS=(primary suture)) OR TS=(primary closure)) OR TS=(primary sutur)) OR TS=(Primary Suturing)) OR TS=(Suture Techniques) |
| 5 | (((((((((((((((((((((((TS=(Cholangiopancreatography, Endoscopic Retrograde)) OR TS=(ERCP)) OR TS=(Retrograde Cholangiopancreatography, Endoscopic)) OR TS=(Cholangiopancreatographies, Endoscopic Retrograde)) OR TS=(Endoscopic Retrograde Cholangiopancreatographies)) OR TS=(Retrograde Cholangiopancreatographies, Endoscopic)) OR TS=(Endoscopic Retrograde Cholangiopancreatography)) OR TS=(Sphincterotomy, Endoscopic)) OR TS=(EST)) OR TS=(Endoscopic Biliary Sphincterotomy)) OR TS=(Endoscopic Biliary Sphincterotomy)) OR TS=(Biliary Sphincterotomy, Endoscopic)) OR TS=(Endoscopic Biliary Sphincterotomies)) OR TS=(Sphincterotomies, Endoscopic Biliary)) OR TS=(Sphincterotomy, Endoscopic Biliary)) OR TS=(Papillotomy, Endoscopic)) OR TS=(Papillotomy, Endoscopic)) OR TS=(Papillotomies, Endoscopic)) OR TS=(Endoscopic Papillotomy)) OR TS=(Endoscopic Sphincterotomy)) OR TS=(Endoscopic Sphincterotomies)) OR TS=(Sphincterotomies, Endoscopic)) OR TS=(endoscopic papillary balloon dilatation )) OR TS=(EPBD) |
| 6 | ((((((TS=(laparoscopic transcystic common bile duct exploration)) OR TS=(Laparoscopic Common Bile Duct Exploration)) OR TS=(Laparoscopic Bile Duct Exploration)) OR TS=(LTCBDE)) OR TS=(Laparoscopic Choledochoscopy)) OR TS=(via cystic duct)) OR TS=(transcystic) |
| 7 | #3 OR #4 OR #5 OR # 6 |
| 8 | #1 And #2 And #7 |
| 9 | (TS=(meta-analysis)) OR TS=(meta analysis) |
| 10 | #8 NOT #9 |
| 11 | (((((((TS=(randomized controlled trial)) OR TS=(controlled clinical trial)) OR TS=(clinical trial)) OR TS=(cohort)) OR TS=(prospective)) OR TS=(retrospective)) OR TS=(comparative study)) OR TS=(observational study) |
| 12 | #10 AND #11 |

**Table S1.3:** Search strategy of Cochrane Central Register of Controlled Trials

| **#** | **Searches** |
| --- | --- |
| 1 | 'gallstone'/exp OR 'biliary calculi':ab,ti OR 'calculi, biliary':ab,ti OR 'gall stone':ab,ti OR 'gall stones':ab,ti OR 'gallstone':ab,ti OR 'common bile duct calculi':ab,ti OR 'biliary calculi, common bile duct':ab,ti OR 'common bile duct gall stones':ab,ti OR 'common bile duct gall stone':ab,ti OR 'common bile duct gallstones':ab,ti OR 'gall stones, common bile duct':ab,ti OR 'common bile duct gallstone':ab,ti OR 'gallstones, common bile duct':ab,ti OR 'cholelithiasis':ab,ti OR 'cholelithiases':ab,ti OR 'gallstone diseases':ab,ti |
| 2 | 'laparoscopic cholecystectomy'/exp OR 'cholecystectomy, celioscopic':ab,ti OR 'celioscopic cholecystectomies':ab,ti OR 'celioscopic cholecystectomy':ab,ti OR 'laparoscopic cholecystectomy':ab,ti OR 'cholecystectomies, laparoscopic':ab,ti OR 'laparoscopic cholecystectomies':ab,ti |
| 3 | 'choledochostomy':ab,ti OR 'choledochostomies':ab,ti OR 'laparoscopic common bile duct exploration':ab,ti OR 'laparoscopic bile duct exploration':ab,ti OR 'laparoscopic choledochoscopy':ab,ti OR 'lcbde':ab,ti OR 'common bile duct exploration':ab,ti |
| 4 | 'primary suture':ab,ti OR 'primary closure':ab,ti OR 'primary sutur':ab,ti OR 'primary suturing':ab,ti OR 'suture techniques':ab,ti |
| 5 | 'cholangiopancreatography, endoscopic retrograde'/exp OR 'sphincterotomy, endoscopic'/exp OR 'ercp':ab,ti OR 'retrograde cholangiopancreatography, endoscopic':ab,ti OR 'cholangiopancreatographies, endoscopic retrograde':ab,ti OR 'endoscopic retrograde cholangiopancreatographies':ab,ti OR 'endoscopic retrograde cholangiopancreatography':ab,ti OR 'est':ab,ti OR 'endoscopic biliary sphincterotomy':ab,ti OR 'biliary sphincterotomies, endoscopic':ab,ti OR 'endoscopic sphincterotomies':ab,ti OR 'sphincterotomy, endoscopic biliary':ab,ti OR 'papillotomy, endoscopic':ab,ti OR 'endoscopic papillotomies':ab,ti OR 'papillotomies, endoscopic':ab,ti OR 'endoscopic papillotomy':ab,ti OR 'endoscopic papillary balloon dilatation':ab,ti OR 'epbd':ab,ti |
| 6 | 'laparoscopic transcystic common bile duct exploration':ab,ti OR 'laparoscopic common bile duct exploration':ab,ti OR 'laparoscopic bile duct exploration':ab,ti OR 'ltcbde':ab,ti OR 'laparoscopic choledochoscopy':ab,ti OR 'via cystic duct':ab,ti OR 'transcystic':ab,ti |
| 7 | #3 OR #4 OR #5 OR # 6 |
| 8 | #1 And #2 And #7 |
| 9 | 'meta analysis':ab,ti OR 'systematic review':ab,ti OR review:ti |
| 10 | #8 not #9 |
| 11 | 'randomized controlled trial':ab,ti OR 'controlled clinical trial':ab,ti OR 'clinical trial':ab,ti OR 'cohort study':ab,ti OR 'cohort studies':ab,ti OR 'prospective study':ab,ti OR 'prospective studies':ab,ti OR 'retrospective study':ab,ti OR 'retrospective studies':ab,ti OR 'comparative study':ab,ti OR 'observational study':ab,ti |
| 12 | #10 And #11 |

**Table S1.4:** Search strategy of Embase

| **#** | **Searches** |
| --- | --- |
| 1 | MeSH descriptor. [Gallstones] explode all trees |
| 2 | (Biliary Calculi):ti,ab,kw OR (Calculi, Biliary):ti,ab,kw OR (Gall Stone*):ti,ab,kw OR (Gallstone):ti,ab,kw OR (Common Bile Duct Calculi):ti,ab,kw OR (Biliary Calculi, Common Bile Duct):ti,ab,kw OR (Common Bile Duct Gall Stone*):ti,ab,kw OR (Gall Stones, Common Bile Duct):ti,ab,kw OR (Common Bile Duct Gallstone*):ti,ab,kw OR (Gallstones, Common Bile Duct):ti,ab,kw OR (Cholelithiasis):ti,ab,kw OR (Cholelithiases):ti,ab,kw OR (Gallstone Disease*):ti,ab,kw |
| 3 | #1 OR #2 |
| 4 | MeSH descriptor: [Cholecystectomy, Laparoscopic] explode all trees |
| 5 | (Cholecystectomy, Celioscopic):ti,ab,kw OR (Celioscopic Cholecystectomies):ti,ab,kw OR (Celioscopic Cholecystectomy):ti,ab,kw OR (Cholecystectomies, Celioscopic):ti,ab,kw OR (Laparoscopic Cholecystectomy):ti,ab,kw OR (Cholecystectomies, Laparoscopic):ti,ab,kw OR (Laparoscopic Cholecystectomies):ti,ab,kw |
| 6 | #4 OR #5 |
| 7 | MeSH descriptor: [Choledochostomy] explode all trees |
| 8 | (Choledochostomies):ti,ab,kw OR (Laparoscopic Common Bile Duct Exploration):ti,ab,kw OR (Laparoscopic Bile Duct Exploration):ti,ab,kw OR (Laparoscopic Choledochoscopy):ti,ab,kw OR (LCBDE):ti,ab,kw |
| 9 | (primary suture):ti,ab,kw OR (primary closure):ti,ab,kw OR (primary sutur):ti,ab,kw OR (Primary Suturing):ti,ab,kw OR (Suture Techniques):ti,ab,kw |
| 10 | (laparoscopic transcystic common bile duct exploration):ti,ab,kw OR (Laparoscopic Common Bile Duct Exploration):ti,ab,kw OR (Laparoscopic Bile Duct Exploration):ti,ab,kw OR (LTCBDE):ti,ab,kw OR (Laparoscopic Choledochoscopy):ti,ab,kw OR (via cystic duct):ti,ab,kw OR (transcystic):ti,ab,kw |
| 11 | MeSH descriptor: [Cholangiopancreatography, Endoscopic Retrograde] explode all trees |
| 12 | (ERCP):ti,ab,kw OR (Retrograde Cholangiopancreatography, Endoscopic):ti,ab,kw OR (Cholangiopancreatographies, Endoscopic Retrograde):ti,ab,kw OR (Endoscopic Retrograde Cholangiopancreatographies):ti,ab,kw OR (Retrograde Cholangiopancreatographies, Endoscopic):ti,ab,kw OR (Endoscopic Retrograde Cholangiopancreatography):ti,ab,kw |
| 13 | MeSH descriptor: [Sphincterotomy, Endoscopic] explode all trees |
| 14 | (EST):ti,ab,kw OR (Endoscopic Biliary Sphincterotomy*):ti,ab,kw OR (Biliary Sphincterotomy*, Endoscopic):ti,ab,kw OR (Sphincterotomy*, Endoscopic Biliary):ti,ab,kw OR (Papillotomy*, Endoscopic):ti,ab,kw OR (Endoscopic Papillotomy*):ti,ab,kw OR (Endoscopic Sphincterotomy*):ti,ab,kw OR (Sphincterotomies, Endoscopic):ti,ab,kw OR (endoscopic papillary balloon dilatation ):ti,ab,kw OR (EPBD):ti,ab,kw |
| 15 | #7 OR #8 OR #9 OR #10 OR #11 OR #12 OR #13 OR #14 |
| 16 | #3 And #6 And #15 |
| 17 | (meta analysis):ti,ab,kw OR (systematic review):ti,ab,kw OR (meta-analys*):ti,ab,kw OR (systematic review*):ti,ab,kw |
| 18 | #16 NOT #17 |
| 19 | (randomized controlled trial):ti,ab,kw OR (controlled clinical trial):ti,ab,kw OR (clinical trial):ti,ab,kw OR (cohort study):ti,ab,kw OR (cohort studies):ti,ab,kw OR (prospective study):ti,ab,kw OR (prospective studies):ti,ab,kw OR (retrospective study):ti,ab,kw OR (retrospective studies):ti,ab,kw OR (comparative study):ti,ab,kw OR (observational study):ti,ab,kw |
| 20 | #18 And #19 |

# Appendix 2: Characteristics of included studies

**Table S2.1:** Baseline of characteristics of included studies

| **Study** | **Country and region** | **Design** | **Follow-up duration** | **Number of participants** | **NMA node mapping** | **Age (mean ± SD),years** | **Male (%)** | **Stone size (mean ± SD),cm** | **CBD diameter (mean ± SD),cm** |
| --- | --- | --- | --- | --- | --- | --- | --- | --- | --- |
| **Farid 2024^1^** | Egypt | RCT | 1 month | 436 | M：218 | 39.56 ±12.31 | 57.34% | NA | NA |
|  |  |  |  |  | I2b：218 | 44.44 ±10.18 | 28.44% | NA | NA |
| **Li 2017^2^** | China | RCT | NA | 140 | I2b：70 | 53.20 ±11.30 | 47.14% | 0.60 ±0.40 | 1.20 ±0.30 |
|  |  |  |  |  | I1：70 | 54.20 ±10.60 | 44.29% | 0.70 ±0.30 | 1.10 ±0.30 |
| **Barreras González 2016^3^** | Cuba | RCT | 1 month | 134 | I2a：46 | 58.40 ±10.67 | NA | NA | 0.82 ±0.27 |
|  |  |  |  |  | I2b：45 | 57.70 ±10.67 | NA | NA | 0.84 ±0.12 |
|  |  |  |  |  | M：43 | 56.30 ±10.83 | NA | NA | 0.77 ±0.27 |
| **Dong 2014^4^** | China | RCT | 12 months | 194 | I1：101 | 52.30 ±13.60 | 48.51% | 1.12 ±0.43 | 1.26 ±0.31 |
|  |  |  |  |  | C：93 | 52.70 ±14.10 | 47.31% | 1.15 ±0.41 | 1.28 ±0.34 |
| **Ding 2014^5^** | China | RCT | 120 months | 221 | C：110 | 58.42 ±7.21 | 48.18% | 0.98 ±0.24 | NA |
|  |  |  |  |  | I2b：111 | 57.53 ±6.31 | 47.75% | 0.99 ±0.27 | NA |
| **Bansal 2014^6^** | India | RCT | 24 months | 168 | M：84 | 45.10 ±15.10 | 27.38% | NA | NA |
|  |  |  |  |  | I2b：84 | 43.00 ±13.70 | 40.48% | NA | NA |
| **Koc 2013^7^** | Turkey | RCT | NA | 111 | I1：57 | 52.30 ±15.20 | 43.86% | NA | 1.24 ±0.07 |
|  |  |  |  |  | I2b：54 | 50.80 ±17.60 | 50.00% | NA | 1.16 ±0.05 |
| **Grubnik 2012^8^** | Ukraine | RCT | NA | 138 | I3：76 | NA | NA | 1.00 ±1.00 | 0.85 ±0.07 |
|  |  |  |  |  | I1：62 | NA | NA | 2.50 ±6.50 | 1.05 ±0.23 |
| **Tzovaras 2012^9^** | Greece | RCT | 6 months | 99 | I2a：50 | 66.00 ±10.83 | 46.00% | NA | 0.90 ±0.27 |
|  |  |  |  |  | I2b：49 | 69.00 ±10.00 | 46.94% | NA | 0.90 ±0.28 |
| **ElGeidie 2011^10^** | Egypt | RCT | 3 months | 226 | M：115 | 32.50 ±7.50 | 25.22% | 0.69 ±0.43 | 0.92 ±0.23 |
|  |  |  |  |  | I2a：111 | 29.20 ±7.83 | 27.93% | 0.59 ±0.35 | 0.79 ±0.18 |
| **ElGeidie 2010^11^** | Egypt | RCT | 14 months | 198 | I2b：100 | 27.50 ±7.50 | 40.85% | 0.85 ±0.60 | 0.92 ±0.22 |
|  |  |  |  |  | I2a：98 | 31.20 ±7.83 | 34.25% | 0.77 ±0.52 | 0.96 ±0.17 |

| **Rogers 2010^12^** | USA | RCT | 24 months | 112 | I2b：55 | 44.60 ±1.90 | 28.07% | NA | NA |
| --- | --- | --- | --- | --- | --- | --- | --- | --- | --- |
|  |  |  |  |  | I3：57 | 39.90 ±1.90 | 30.91% | NA | NA |
| **Bansal 2010^13^** | India | RCT | NA | 30 | C：15 | 47.10 ±9.50 | 26.67% | NA | NA |
|  |  |  |  |  | I2b：15 | 39.10 ±10.30 | 33.33% | NA | NA |
| **ElGeidie 2009^14^** | Egypt | RCT | 2 months | 122 | I1：61 | 43.00 ±7.17 | 36.07% | NA | 1.12 ±0.83 |
|  |  |  |  |  | C：61 | 39.00 ±8.50 | 26.23% | NA | 1.16 ±0.92 |
| **Zhang 2009^15^** | China | RCT | 24 months | 93 | I1：47 | 52.30 ±16.60 | 46.81% | 1.08 ±0.37 | 1.22 ±0.35 |
|  |  |  |  |  | C：46 | 52.00 ±15.90 | 41.30% | 1.12 ±0.38 | 1.19 ±0.39 |
| **Noble 2009^16^** | UK | RCT | 22.6 months | 91 | I2b：47 | 74.30 ±1.48 | 46.81% | NA | 1.00 ±1.08 |
|  |  |  |  |  | I1：44 | 75.90 ±1.80 | 36.36% | NA | 0.70 ±1.33 |
| **Zhang 2008^17^** | China | RCT | 54 months | 80 | I1：40 | 52.00 ±14.00 | 42.50% | NA | 1.50 ±0.40 |
|  |  |  |  |  | C：40 | 45.00 ±12.00 | 45.00% | NA | 1.60 ±0.60 |
| **Morino 2006^18^** | Italy | RCT | 20 months | 91 | I2b：45 | 63.10 ±9.67 | 44.44% | NA | NA |
|  |  |  |  |  | I2a：46 | 56.60 ±10.00 | 32.61% | NA | NA |
| **Rábago 2006^19^** | Spain | RCT | 24 months | 123 | I2b：64 | NA | NA | NA | NA |
|  |  |  |  |  | I2a：59 | NA | NA | NA | NA |
| **Hajong 2025^20^** | India | Prospective cohort | NA | 72 | I1：36 | 39.27 ±11.20 | 19.44% | 1.75 ±0.92 | 1.22 ±0.27 |
|  |  |  |  |  | I2b：36 | 38.73 ±10.90 | 22.22% | 1.62 ±0.87 | 1.16 ±0.31 |
| **Mohamed 2015^21^** | Egypt | Prospective cohort | NA | 150 | M：75 | 47.10 ±6.33 | 34.67% | NA | NA |
|  |  |  |  |  | I2b：75 | 39.00 ±6.83 | 36.00% | NA | NA |
| **Zhang 2009^22^** | China | Prospective cohort | 24 months | 330 | I3：237 | 54.70 ±13.30 | 41.35% | 0.53 ±0.21 | NA |
|  |  |  |  |  | I1：47 | 52.30 ±16.60 | 46.81% | 1.19 ±0.27 | NA |
|  |  |  |  |  | C：46 | 52.00 ±15.90 | 41.30% | 1.23 ±0.34 | NA |
| **Hong 2006^23^** | China | Prospective cohort | 27 months | 234 | I2a：93 | NA | 30.11% | NA | NA |
|  |  |  |  |  | M：141 | NA | 46.10% | NA | NA |
| **Wright 2002^24^** | USA | Prospective cohort | NA | 67 | I2b：53 | 42.70 ±19.10 | 18.87% | NA | NA |
|  |  |  |  |  | I2a：14 | 49.00 ±18.70 | 21.43% | NA | NA |
| **Zhou 2025^25^** | China | Retrospective cohort | 60 months | 180 | I2b：120 | 61.30 ±12.10 | 50.83% | NA | 1.20 ±0.24 |
|  |  |  |  |  | C：60 | 61.72 ±14.06 | 53.33% | NA | 1.20 ±0.29 |

| **Liu 2025^26^** | China | Retrospective cohort | 3 months | 318 | M：152 | 61.63 ±12.78 | 53.29% | 1.01 ±0.32 | NA |
| --- | --- | --- | --- | --- | --- | --- | --- | --- | --- |
|  |  |  |  |  | I2b：166 | 59.46 ±11.06 | 45.78% | 0.97 ±0.20 | NA |
| **Li 2025^27^** | China | Retrospective cohort | 6 months | 86 | C：43 | 67.34 ±3.20 | 44.19% | 0.79 ±0.21 | NA |
|  |  |  |  |  | I2b：43 | 67.45 ±3.53 | 46.51% | 0.91 ±0.21 | NA |
| **Fang 2025^28^** | China | Retrospective cohort | 12 months | 206 | I1：38 | 53.70 ±9.82 | 47.37% | 1.34 ±0.67 | 1.56 ±0.55 |
|  |  |  |  |  | I2b：96 | 61.70 ±17.03 | 53.13% | 0.91 ±0.59 | 1.14 ±0.40 |
|  |  |  |  |  | C：72 | 58.10 ±15.41 | 41.67% | 1.27 ±0.82 | 1.36 ±0.46 |
| **Abdelkader 2025^29^** | UK | Retrospective cohort | 1.5 months | 38 | I3：21 | 49.78 ±18.44 | 33.33% | NA | NA |
|  |  |  |  |  | I2b：17 | 50.31 ±15.14 | 47.06% | NA | NA |
| **Anwar 2025^30^** | Egypt | Retrospective cohort | 12 months | 100 | I2b：50 | 43.60 ±16.02 | 28.00% | NA | NA |
|  |  |  |  |  | M：50 | 53.76 ±15.45 | 46.00% | NA | NA |
| **Volkovetskii 2025^31^** | Ukraine | Retrospective cohort | NA | 136 | I1：71 | 61.60 ±16.60 | 35.21% | NA | NA |
|  |  |  |  |  | I2b：65 | 69.20 ±11.00 | 36.92% | NA | NA |
| **Yang 2025^32^** | China | Retrospective cohort | NA | 102 | I3：51 | 56.49 ±14.67 | 47.06% | NA | NA |
|  |  |  |  |  | M：51 | 52.23 ±17.10 | 56.86% | NA | NA |
| **Xu 2025^33^** | China | Retrospective cohort | 6 months | 127 | I1：67 | 71.17 ±6.26 | 38.81% | 2.83 ±1.03 | 1.05 ±0.15 |
|  |  |  |  |  | C：60 | 70.35 ±5.17 | 36.67% | 2.79 ±1.08 | 1.07 ±0.15 |
| **Wu 2024^34^** | Taiwan | Retrospective cohort | 36 months | 160 | M：80 | 80.60 ±6.90 | 42.50% | NA | 1.51 ±0.50 |
|  |  |  |  |  | I2b：80 | 77.60 ±6.00 | 62.50% | NA | 0.82 ±0.32 |
| **DeJesus 2024^35^** | UK | Retrospective cohort | 1 month | 154 | I3：53 | 44.60 ±19.60 | 35.85% | NA | 0.78 ±0.30 |
|  |  |  |  |  | I2b：101 | 47.16 ±20.42 | 30.69% | NA | 0.75 ±0.29 |
| **Zhao 2023^36^** | China | Retrospective cohort | 6 months | 88 | I1：50 | 58.88 ±13.75 | 38.00% | NA | NA |
|  |  |  |  |  | C：38 | 61.76 ±13.13 | 55.26% | NA | NA |
| **Xie 2023^37^** | China | Retrospective cohort | 48.55 months | 526 | M：364 | 63.67 ±11.85 | 46.70% | NA | 1.10 ±0.35 |
|  |  |  |  |  | I2b：162 | 63.33 ±10.74 | 61.73% | NA | 1.00 ±0.30 |
| **Wang 2023^38^** | China | Retrospective cohort | 6 months | 975 | I1：690 | 61.70 ± 14.10 | 48.60% | 0.64 ±0.02 | 1.13 ±0.25 |
|  |  |  |  |  | I2b：285 | 61.10 ± 13.20 | 62.50% | 0.55 ±0.03 | 1.02 ±0.33 |
| **Tian 2023^39^** | China | Retrospective cohort | 120 months | 1005 | M：431 | 55.81 ±16.74 | 50.58% | NA | 1.15 ±0.41 |
|  |  |  |  |  | I2a：574 | 56.16 ±13.86 | 50.52% | NA | 1.16 ±0.25 |
| **Wang 2022^40^** | China | Retrospective cohort | 18 months | 255 | I1：193 | 64.00 ±15.00 | 40.93% | NA | 1.23 ±0.28 |
|  |  |  |  |  | C：62 | 66.00 ±13.00 | 36.36% | NA | 1.24 ±0.25 |

| **Lee 2022^41^** | South Korea | Retrospective cohort | 6 months | 137 | I1：46 | 84.10 ±3.80 | 50.00% | 1.19 ±0.79 | 1.39 ±0.52 |
| --- | --- | --- | --- | --- | --- | --- | --- | --- | --- |
|  |  |  |  |  | I2b：91 | 83.50 ±2.90 | 49.45% | 0.60 ±0.41 | 1.06 ±0.35 |
| **Lee 2022^42^** | South Korea | Retrospective cohort | 17 months | 358 | I1：92 | 64.70 ±15.00 | 51.09% | 1.29 ±0.41 | 1.28 ±0.34 |
|  |  |  |  |  | I2b：266 | 66.00 ±14.70 | 48.12% | 1.11 ±0.37 | 1.15 ±0.41 |
| **Guo 2022^43^** | China | Retrospective cohort | 72 months | 1034 | I2b：275 | 51.79 ±15.68 | 53.45% | 0.82 ±0.46 | 0.81 ±0.27 |
|  |  |  |  |  | I3：280 | 52.11 ±15.06 | 49.29% | 0.82 ±0.38 | 0.93 ±0.28 |
|  |  |  |  |  | I1：479 | 51.65 ±16.07 | 55.95% | 0.93 ±0.45 | 1.11 ±0.32 |
| **De Silva 2022^44^** | UK | Retrospective cohort | 1 month | 671 | M：578 | 61.80 ±16.30 | 39.97% | 0.40 ±0.06 | NA |
|  |  |  |  |  | I2a：13 | 71.20 ±10.10 | 38.46% | 0.53 ±0.08 | NA |
|  |  |  |  |  | I2b：80 | 65.40 ±17.00 | 42.50% | 0.51 ±0.21 | NA |
| **Di Lascia 2021^45^** | Italy | Retrospective cohort | NA | 40 | I2b：20 | 62.52 ±11.60 | 35.00% | NA | NA |
|  |  |  |  |  | I2a：20 | 56.62 ±11.40 | 30.00% | NA | NA |
| **Liu 2020^46^** | China | Retrospective cohort | 76 months | 207 | I2a：103 | 57.10 ±10.50 | 40.78% | 1.20 ±0.30 | 1.30 ±0.30 |
|  |  |  |  |  | I1 ：104 | 56.90 ±9.83 | 39.42% | 1.10 ±0.40 | 1.20 ±0.40 |
| **Zhen 2020^47^** | China | Retrospective cohort | 32.4 months | 80 | I1：51 | 56.40 ±9.50 | 54.90% | NA | 1.87 ±0.46 |
|  |  |  |  |  | C：29 | 59.20 ±8.00 | 51.72% | NA | 1.79 ±0.55 |
| **Jiang 2019^48^** | China | Retrospective cohort | 6 months | 51 | I2b：29 | 62.20 ±10.40 | 55.17% | 1.08 ±0.64 | 0.85 ±0.31 |
|  |  |  |  |  | I2a：22 | 56.50 ±9.80 | 59.09% | 1.15 ±0.71 | 0.63 ±0.14 |
| **Zhou 2019^49^** | China | Retrospective cohort | 28.4 months | 100 | M：54 | 83.39 ±2.86 | 48.15% | NA | NA |
|  |  |  |  |  | I2b：46 | 84.24 ±2.89 | 63.04% | NA | NA |
| **Passi 2018^50^** | USA | Retrospective cohort | 1 month | 214 | I2a：37 | 41.10 ±26.50 | 48.65% | 0.61 ±0.47 | 0.77 ±0.35 |
|  |  |  |  |  | I2b：177 | 51.30 ±21.60 | 24.86% | 0.86 ±0.53 | 0.91 ±0.40 |
| **Mattila 2017^51^** | Finland | Retrospective cohort | NA | 217 | M：97 | 59.10 ±19.00 | 19.59% | NA | NA |
|  |  |  |  |  | I2b：120 | 64.80 ±15.30 | 46.67% | NA | NA |
| **Wei 2003^52^** | China | Retrospective cohort | NA | 102 | M：45 | NA | 37.78% | NA | NA |
|  |  |  |  |  | I2a：57 | NA | 35.09% | NA | NA |

Abbreviations: C, LC + LCBDE + T-tube drainage; I1, LC + LCBDE + PS; I2a, LC + ERCP (one-stage); I2b, LC + ERCP (two-stage); I3, LC + LTCBDE; M, LC + LCBDE (mixed interventions); CBD, common bile duct; LC, laparoscopic cholecystectomy; LCBDE, laparoscopic common bile duct exploration; LTCBDE, laparoscopic transcystic bile duct exploration; ERCP, endoscopic retrograde cholangiopancreatography; PS, primary suture; SSI, surgical site infection; NA, Not Available.

# Appendix 3: List of data extracted from the included studies

| **Data category** | **List of variables** |
| --- | --- |
| Study | First author, year of publication, country and region, study design (Randomized controlled trial or Cohort study), follow-up duration, total number of participants and sample size per treatment arm |
| Patients | Age (years), sex (%), stone size (cm), CBD diameter (cm) |
| Interventions | LC + LCBDE + TTD; LC + LCBDE + PS; LC + ERCP (one-stage); LC + ERCP (two-stage); LC + LTCBDE; LC + LCBDE (mixed) |
| Primary outcomes | Single-session stone clearance rate; Rate of severe postoperative complications |
| Secondary outcomes – perioperative | Operative time (min); Intraoperative blood loss (mL); Total length of hospital stay (days); Conversion to open surgery rate; Reintervention rate |
| Secondary outcomes – postoperative complications | Postoperative bile leakage rate; Postoperative pancreatitis rate; Postoperative biliary bleeding rate; Postoperative SSI rate; Postoperative intra-abdominal infection rate |
| Long-term outcomes | Rate of symptomatic biliary stricture; Stone recurrence rate |
| Health economic outcome | Total hospital cost |

Abbreviations: CBD,common bile duct;LC, laparoscopic cholecystectomy; LCBDE, laparoscopic common bile duct exploration;LTCBDE, laparoscopic transcystic bile duct exploration; ERCP, endoscopic retrograde cholangiopancreatography; PS,primary suture ;TTD,T-tube drainage;SSI, surgical site infection.

# Appendix 4: Risk of bias and quality assessment of included studies

**Figure S4:** Overall risk of bias presented as percentage of each risk of bias item across all included studies. Green = Low risk, Red = High risk, Yellow = Some concerns.

**Table S4.1:** Study level risk of bias assessment using Cochrane risk of bias tool 2.0 for assessing risk of bias of randomized clinical trials.

| **Unique ID** | **Study ID** | **Randomization process** | **Deviations from intended interventions** | **Missing outcome data** | **Measurement of the outcome** | **Selection of the reported result** | **Overall** |
| --- | --- | --- | --- | --- | --- | --- | --- |
| **Farid 2024** | NCT05734144 | Low | Low | Low | Low | Low | Low risk |
| **Li 2017** | NA | Low | Low | Low | Low | Some concerns | Some concerns |
| **Barreras González 2016** | RPCEC00000013 | Low | Low | Low | Low | Low | Low risk |
| **Dong 2014** | NA | Low | Low | Low | Low | Some concerns | Some concerns |
| **Ding 2014** | NA | Low | Low | Low | Low | Some concerns | Some concerns |
| **Bansal 2014** | CTRI/2012/09/002979 | Low | Low | Low | Low | Low | Low risk |
| **Koc 2013** | NA | Low | Some concerns | Some concerns | Low | Some concerns | Some concerns |
| **Grubnik 2012** | NA | Low | Low | Low | Low | Some concerns | Some concerns |
| **Tzovaras 2012** | NCT00416234 | Low | Low | Low | Low | Low | Low risk |
| **ElGeidie 2011** | NA | Low | Low | Low | Low | Some concerns | Some concerns |
| **Rogers 2010** | NCT00807729 | Low | Some concerns | Low | Low | Some concerns | Some concerns |
| **ElGeidie 2011** | NA | Low | Low | Low | Low | Some concerns | Some concerns |
| **Bansal 2010** | NA | Low | Some concerns | Some concerns | Low | Some concerns | Some concerns |
| **Zhang 2009** | NA | Low | Low | Low | Low | Some concerns | Some concerns |
| **ElGeidie 2010** | NA | Low | Low | Low | Low | Some concerns | Some concerns |
| **Noble 2009** | NA | Low | Low | Low | Low | Some concerns | Some concerns |
| **Zhang 2008** | NA | Low | Low | Low | Low | Some concerns | Some concerns |
| **Morino 2006** | NA | Low | Low | Low | Low | Some concerns | Some concerns |
| **Rábago 2006** | NA | Low | Low | Low | Low | Some concerns | Some concerns |

Each domain was judged as “low risk,” “some concerns,” or “high risk.” The overall risk of bias was derived according to the Cochrane RoB 2.0 guidance. Abbreviations: NA, Not Available.

**Table S4.2:** Quality assessment of cohort studies using the Newcastle–Ottawa Scale(NOS).

| **Study** | **Selection of study groups** | **Comparability between groups** | **Outcome assessment** | **Total score** |
| --- | --- | --- | --- | --- |
| **Zhou 2025** | 4 | 2 | 3 | 9 |
| **Liu 2025** | 4 | 1 | 1 | 6 |
| **Li 2025** | 4 | 1 | 2 | 7 |
| **Hajong 2025** | 4 | 1 | 2 | 7 |
| **Fang 2025** | 4 | 1 | 3 | 8 |
| **Abdelkader 2025** | 4 | 1 | 2 | 7 |
| **Anwar 2025** | 4 | 1 | 3 | 8 |
| **Volkovetskii 2025** | 4 | 1 | 2 | 7 |
| **Yang 2025** | 4 | 1 | 2 | 7 |
| **Xu 2025** | 4 | 1 | 2 | 7 |
| **Wu 2024** | 4 | 1 | 3 | 8 |
| **DeJesus 2024** | 4 | 2 | 3 | 9 |
| **Zhao 2023** | 4 | 1 | 2 | 7 |
| **Xie 2023** | 4 | 2 | 3 | 9 |
| **Wang 2023** | 4 | 2 | 3 | 9 |
| **Tian 2023** | 4 | 2 | 3 | 9 |
| **Wang 2022** | 4 | 1 | 3 | 8 |
| **Lee 2022** | 4 | 1 | 2 | 7 |
| **Lee 2022** | 4 | 1 | 3 | 8 |
| **Guo 2022** | 4 | 1 | 2 | 7 |
| **De Silva 2022** | 4 | 1 | 2 | 7 |
| **Di Lascia 2021** | 4 | 1 | 2 | 7 |
| **Liu 2020** | 4 | 1 | 3 | 8 |
| **Zhen 2020** | 4 | 1 | 3 | 8 |
| **Jiang 2019** | 4 | 1 | 2 | 7 |
| **Zhou 2019** | 4 | 1 | 3 | 8 |
| **Passi 2018** | 4 | 1 | 2 | 7 |
| **Mattila 2017** | 4 | 1 | 2 | 7 |
| **Mohamed 2015** | 4 | 1 | 2 | 7 |
| **Zhang 2009** | 4 | 1 | 3 | 8 |
| **Hong 2006** | 4 | 1 | 2 | 7 |
| **Wei 2003** | 4 | 1 | 1 | 6 |
| **Wright 2002** | 4 | 1 | 1 | 6 |

Methodological quality was assessed using the Newcastle–Ottawa Scale (NOS), which evaluates selection of cohorts, comparability of study groups, and outcome assessment. A maximum of nine stars can be awarded. Studies scoring ≥7 were considered high quality.

# Appendix 5: Evaluation of inconsistency and heterogeneity

**5.1 Global consistency**

**Table S5.1a:** Global consistency of randomized controlled trials (RCTs)

| **Clinical outcome** | **Chi square** | **P value** | **τ²** |
| --- | --- | --- | --- |
| Single-session stone clearance rate | 3.54 | 0.618 | 0.001 |
| Severe postoperative complications | 4.11 | 0.662 | 2.59 × 10⁻²⁰ |
| Operative time | 8.56 | 0.073 | 0.830 |
| Total length of hospital stay | 3.57 | 0.312 | 0.750 |
| Conversion to open surgery | 0.95 | 0.966 | 9.44 × 10⁻¹⁶ |
| Reintervention | 0.93 | 0.817 | 1.58 × 10⁻¹⁸ |
| Postoperative bile leakage | 0.92 | 0.921 | 7.99 × 10⁻¹⁹ |
| Postoperative pancreatitis | 1.98 | 0.739 | 4.41 × 10⁻¹⁰ |
| Postoperative biliary bleeding | 0.24 | 0.885 | 5.88 × 10⁻²⁰ |
| Postoperative surgical site infection | 0.22 | 0.897 | 1.36×10^−19^ |
| Postoperative intra-abdominal infection | 1.07 | 0.586 | 3.37×10^−16^ |
| Postoperative cholangitis | 0.6 | 0.742 | 6.57×10^−19^ |
| symptomatic biliary stricture | 1.07 | 0.301 | 2.02×10^−11^ |
| Stone recurrence rate | 2.93 | 0.232 | 0.4119 |

**Table S5.1b:** Global consistency of cohort studies

| **Clinical outcome** | **Chi square** | **P value** | **τ²** |
| --- | --- | --- | --- |
| Single-session stone clearance rate | 8.17 | 0.612 | 0.002 |
| Severe postoperative complications | 4.64 | 0.591 | 1.5 × 10⁻¹⁷ |
| Operative time | 12.88 | 0.168 | 4.166 |
| Intraoperative blood loss | 5.04 | 0.169 | 3.046 |
| Total length of hospital stay | 10.55 | 0.568 | 1.503 |
| Conversion to open surgery | 4.40 | 0.927 | 1.15 × 10⁻¹⁶ |
| Reintervention | 2.47 | 0.988 | 0.339 |
| Postoperative bile leakage | 11.27 | 0.337 | 3.75 × 10⁻¹³ |
| Postoperative pancreatitis | 37.27 | 0.058 | 0.857 |
| Postoperative biliary bleeding | 4.47 | 0.484 | 1.57×10^−19^ |
| Postoperative surgical site infection | 1.64 | 0.801 | 1.60×10^−16^ |
| Postoperative intra-abdominal infection | 0.46 | 0.795 | 4.20×10^−9^ |
| Postoperative cholangitis | 7.83 | 0.348 | 3.04 × 10⁻¹⁹ |
| symptomatic biliary stricture | 0.36 | 0.550 | 2.12×10^−20^ |
| Stone recurrence rate | 8.17 | 0.417 | 0.147 |

**5.2 Single-session stone clearance rate**

**Table S5.2a:** Side-splitting of single-session stone clearance rate in RCTs. Inconsistency test between direct and indirect treatment comparisons in mixed treatment comparison.

| Comparison | Direct | | Indirect | | Difference | | | tau |
| --- | --- | --- | --- | --- | --- | --- | --- | --- |
|  | Coef. | Std. Err. | Coef. | Std. Err. | Coef. | Std. Err. | P>\|z\| |  |
| LC + LCBDE + PS VS LC + ERCP (two-stage) | -0.0918045 | 0.0314495 | 0.0063312 | 0.0339544 | -0.0981357 | 0.0462814 | 0.034 | 4.31E-07 |
| LC + LCBDE + PS VS LC + LTCBDE | 0.0126242 | 0.0584595 | -0.1691715 | 0.1116678 | 0.1817957 | 0.1260445 | 0.149 | 0.0396759 |
| LC + LCBDE + PS VS LC + LCBDE + TTD | -0.0023878 | 0.0245004 | -0.0778419 | 0.0554777 | 0.0754541 | 0.0619433 | 0.223 | 0.0284734 |
| LC + ERCP (one-stage) VS LC + ERCP (two-stage) | -0.0253406 | 0.0317322 | -0.0819892 | 0.0731026 | 0.0566485 | 0.0788091 | 0.472 | 0.0486641 |
| LC + ERCP (one-stage) VS LC + LCBDE (mixed) | -0.0284669 | 0.042222 | 0.0139373 | 0.0570722 | -0.0424042 | 0.0709705 | 0.55 | 0.0502221 |
| LC + ERCP (two-stage) VS LC + LTCBDE | -0.0923733 | 0.1024333 | 0.0894224 | 0.0734357 | -0.1817958 | 0.1260372 | 0.149 | 0.0396759 |
| LC + ERCP (two-stage) VS LC + LCBDE (mixed) | 0.0340878 | 0.0353245 | -0.0324247 | 0.0602882 | 0.0665124 | 0.0688913 | 0.334 | 0.0412808 |
| LC + ERCP (two-stage) VS LC + LCBDE + TTD | 0.0051984 | 0.053052 | 0.0806537 | 0.064802 | -0.0754553 | 0.0619262 | 0.223 | 0.0284744 |

Abbreviations: LC, laparoscopic cholecystectomy; LCBDE, laparoscopic common bile duct exploration; LTCBDE, laparoscopic transcystic bile duct exploration; ERCP, endoscopic retrograde cholangiopancreatography; PS, primary suture; TTD, T-tube drainage.

**Table S5.2b:** Side-splitting of single-session stone clearance rate in Cohort studies. Inconsistency test between direct and indirect treatment comparisons in mixed treatment comparison.

| Comparison | Direct | | Indirect | | Difference | | | tau |
| --- | --- | --- | --- | --- | --- | --- | --- | --- |
|  | Coef. | Std. Err. | Coef. | Std. Err. | Coef. | Std. Err. | P>\|z\| |  |
| LC + LCBDE + PS VS LC + ERCP (one-stage) | 0.1417216 | 0.0664352 | -0.017083 | 0.034942 | 0.1588046 | 0.0750639 | 0.034 | 0.0407895 |
| LC + LCBDE + PS VS LC + ERCP (two-stage) | -0.0662117 | 0.0234937 | 0.003989 | 0.035409 | -0.0702006 | 0.042556 | 0.099 | 0.0433349 |
| LC + LCBDE + PS VS LC + LTCBDE | 0.0111263 | 0.0361404 | -0.0345797 | 0.0438323 | 0.0457059 | 0.0578591 | 0.43 | 0.04497 |
| LC + LCBDE + PS VS LC + LCBDE + TTD | -0.0013892 | 0.0240438 | -0.0337298 | 0.0471966 | 0.0323406 | 0.0532715 | 0.544 | 0.0459876 |
| LC + ERCP (one-stage) VS LC + ERCP (two-stage) | -0.0363302 | 0.0366015 | -0.0977999 | 0.0420299 | 0.0614697 | 0.0549645 | 0.263 | 0.0441478 |
| LC + ERCP (one-stage) VS LC + LCBDE (mixed) | -0.0040315 | 0.0431444 | -0.0256428 | 0.0408307 | 0.0216112 | 0.0585313 | 0.712 | 0.04603 |
| LC + ERCP (two-stage) VS LC + LTCBDE | 0.0307604 | 0.0377087 | 0.0444514 | 0.0386912 | -0.013691 | 0.0541364 | 0.8 | 0.0464789 |
| LC + ERCP (two-stage) VS LC + LCBDE (mixed) | 0.0460059 | 0.0221296 | 0.0535181 | 0.0448929 | -0.0075121 | 0.0501194 | 0.881 | 0.0463851 |
| LC + ERCP (two-stage) VS LC + LCBDE + TTD | 0.0161052 | 0.0436472 | 0.0469843 | 0.0307648 | -0.0308791 | 0.0533847 | 0.563 | 0.0459491 |
| LC + LTCBDE VS  LC + LCBDE (mixed) | 0.0202027 | 0.0580178 | 0.006114 | 0.0362894 | 0.0140887 | 0.0684324 | 0.837 | 0.0466218 |
| LC + LTCBDE VS  LC + LCBDE + TTD | 0.0146994 | 0.0532679 | -0.008745 | 0.0384883 | 0.0234444 | 0.0659219 | 0.722 | 0.0468429 |

Abbreviations: LC, laparoscopic cholecystectomy; LCBDE, laparoscopic common bile duct exploration; LTCBDE, laparoscopic transcystic bile duct exploration; ERCP, endoscopic retrograde cholangiopancreatography; PS, primary suture; TTD, T-tube drainage.

**5.3 Severe postoperative complications**

**Table S5.3a:** Side-splitting of severe postoperative complications in RCTs. Inconsistency test between direct and indirect treatment comparisons in mixed treatment comparison.

| Comparison | Direct | | Indirect | | Difference | | | tau |
| --- | --- | --- | --- | --- | --- | --- | --- | --- |
|  | Coef. | Std. Err. | Coef. | Std. Err. | Coef. | Std. Err. | P>\|z\| |  |
| LC + LCBDE + PS VS LC + ERCP (one-stage) | 0.9566335 | 1.211362 | -1.329376 | 0.9374569 | 2.28601 | 1.548702 | 0.14 | 6.42E-10 |
| LC + LCBDE + PS VS LC + ERCP (two-stage) | 0.4859201 | 0.5401381 | 2.104526 | 1.541572 | -1.618606 | 1.594142 | 0.31 | 1.94E-09 |
| LC + LCBDE + PS VS LC + LCBDE + TTD | 0.8566769 | 0.5103341 | 0.6256936 | 2.07548 | 0.2309834 | 2.137302 | 0.914 | 8.03E-10 |
| LC + ERCP (one-stage) VS LC + ERCP (two-stage) | 1.053437 | 0.5970823 | 1.481379 | 1.655768 | -0.4279421 | 1.736597 | 0.805 | 1.22E-13 |
| LC + ERCP (one-stage) VS LC + LCBDE (mixed) | 1.336497 | 1.071335 | -1.993971 | 1.425168 | 3.330468 | 1.93422 | 0.085 | 4.81E-09 |
| LC + ERCP (two-stage) VS LC + LTCBDE | -0.0354019 | 2.008833 | -1.298094 | 200.01 | 1.262693 | 199.9999 | 0.995 | 7.73E-09 |
| LC + ERCP (two-stage) VS LC + LCBDE (mixed) | -1.360279 | 0.6859885 | 0.9530326 | 1.635642 | -2.313311 | 1.752717 | 0.187 | 6.50E-10 |

Abbreviations: LC, laparoscopic cholecystectomy; LCBDE, laparoscopic common bile duct exploration; LTCBDE, laparoscopic transcystic bile duct exploration; ERCP, endoscopic retrograde cholangiopancreatography; PS, primary suture; TTD, T-tube drainage.

**Table S5.3b:** Side-splitting of severe postoperative complications in Cohort studies. Inconsistency test between direct and indirect treatment comparisons in mixed treatment comparison.

| Comparison | Direct | | Indirect | | Difference | | | tau |
| --- | --- | --- | --- | --- | --- | --- | --- | --- |
|  | Coef. | Std. Err. | Coef. | Std. Err. | Coef. | Std. Err. | P>\|z\| |  |
| LC + LCBDE + PS VS LC + ERCP (two-stage) | 0.0010532 | 0.5085498 | -0.6703823 | 1.240786 | 0.6714355 | 1.340914 | 0.617 | 3.55E-09 |
| LC + LCBDE + PS VS LC + LCBDE + TTD | -0.8885387 | 0.9671712 | -0.1651231 | 0.8077008 | -0.7234156 | 1.260097 | 0.566 | 3.99E-06 |
| LC + ERCP (one-stage) VS LC + ERCP (two-stage) | 0.4574902 | 0.716992 | -1.34801 | 1.109161 | 1.8055 | 1.096598 | 0.1 | 7.69E-10 |
| LC + ERCP (one-stage) VS LC + LCBDE (mixed) | -0.5761036 | 0.7041755 | 0.5711236 | 1.104949 | -1.147227 | 1.08003 | 0.288 | 1.63E-08 |
| LC + ERCP (two-stage) VS LC + LTCBDE | -0.2149077 | 0.6716497 | -0.0239024 | 141.3935 | -0.1910053 | 141.3919 | 0.999 | 9.13E-07 |
| LC + ERCP (two-stage) VS LC + LCBDE (mixed) | -0.4438381 | 0.2939827 | -0.8288619 | 2.086106 | 0.3850238 | 2.107112 | 0.855 | 2.82E-09 |
| LC + ERCP (two-stage) VS LC + LCBDE + TTD | -0.196008 | 0.5870775 | -1.074998 | 1.195196 | 0.8789902 | 1.331578 | 0.509 | 2.08E-06 |

Abbreviations: LC, laparoscopic cholecystectomy; LCBDE, laparoscopic common bile duct exploration; LTCBDE, laparoscopic transcystic bile duct exploration; ERCP, endoscopic retrograde cholangiopancreatography; PS, primary suture; TTD, T-tube drainage.

**5.4 Operative time**

**Table S5.4a:** Side-splitting of operative time in RCTs. Inconsistency test between direct and indirect treatment comparisons in mixed treatment comparison.

| Comparison | Direct | | Indirect | | Difference | | | tau |
| --- | --- | --- | --- | --- | --- | --- | --- | --- |
|  | Coef. | Std. Err. | Coef. | Std. Err. | Coef. | Std. Err. | P>\|z\| |  |
| LC + LCBDE + PS VS LC + ERCP (two-stage) | -15.86798 | 9.409489 | -18.2659 | 18.10233 | 2.397925 | 20.40282 | 0.906 | 12.64825 |
| LC + LCBDE + PS VS LC + LTCBDE | -28 | 13.08039 | -24.7414 | 18.74215 | -3.258599 | 22.8553 | 0.887 | 12.76054 |
| LC + ERCP (one-stage) VS LC + ERCP (two-stage) | -19.31957 | 6.283006 | -30.07482 | 17.59859 | 10.75525 | 18.70884 | 0.565 | 12.52771 |
| LC + ERCP (one-stage) VS LC + LCBDE (mixed) | 1.094502 | 10.34069 | 11.1601 | 13.71246 | -10.0656 | 17.0116 | 0.554 | 12.43943 |
| LC + ERCP (two-stage) VS LC + LTCBDE | -9.091171 | 16.4051 | -11.92441 | 16.0002 | 2.833244 | 22.90596 | 0.902 | 12.76697 |
| LC + ERCP (two-stage) VS LC + LCBDE (mixed) | 33.07512 | 8.943103 | 5.907385 | 12.29521 | 27.16774 | 14.83039 | 0.067 | 10.62557 |

Abbreviations: LC, laparoscopic cholecystectomy; LCBDE, laparoscopic common bile duct exploration; LTCBDE, laparoscopic transcystic bile duct exploration; ERCP, endoscopic retrograde cholangiopancreatography; PS, primary suture; TTD, T-tube drainage.

**Table S5.4b:** Side-splitting of operative time in cohort studies. Inconsistency test between direct and indirect treatment comparisons in mixed treatment comparison.

| Comparison | Direct | | Indirect | | Difference | | | tau |
| --- | --- | --- | --- | --- | --- | --- | --- | --- |
|  | Coef. | Std. Err. | Coef. | Std. Err. | Coef. | Std. Err. | P>\|z\| |  |
| LC + LCBDE + PS VS LC + ERCP (one-stage) | -20 | 28.64299 | 22.77454 | 15.83478 | -42.77454 | 32.7286 | 0.191 | 28.03668 |
| LC + LCBDE + PS VS LC + ERCP (two-stage) | -14.79641 | 16.91924 | -3.272082 | 15.90287 | -11.52433 | 23.21403 | 0.62 | 28.96931 |
| LC + LCBDE + PS VS LC + LTCBDE | -18.35799 | 20.57935 | -38.44353 | 27.42992 | 20.08554 | 34.31768 | 0.558 | 28.99165 |
| LC + LCBDE + PS VS LC + LCBDE + TTD | 16.17091 | 12.07886 | -38.47295 | 21.55738 | 54.64386 | 24.70536 | 0.027 | 26.21387 |
| LC + ERCP (one-stage) VS LC + ERCP (two-stage) | -31.63297 | 13.30472 | -0.2977662 | 19.06356 | -31.33521 | 23.26541 | 0.178 | 27.93889 |
| LC + ERCP (one-stage) VS LC + LCBDE (mixed) | 4.694892 | 17.03895 | -4.963651 | 19.65927 | 9.658543 | 26.01721 | 0.71 | 29.08958 |
| LC + ERCP (two-stage) VS LC + LTCBDE | -33.64476 | 29.10669 | -8.568689 | 20.5846 | -25.07607 | 35.6649 | 0.482 | 28.98579 |
| LC + ERCP (two-stage) VS LC + LCBDE (mixed) | 20.54008 | 13.84941 | 25.11916 | 20.19378 | -4.579079 | 24.45531 | 0.851 | 29.20828 |
| LC + ERCP (two-stage) VS LC + LCBDE + TTD | -24.2594 | 18.20913 | 38.6976 | 15.87677 | -62.957 | 24.15906 | 0.009 | 25.30586 |
| LC + LTCBDE VS LC + LCBDE (mixed) | 23.99864 | 29.0358 | 47.93481 | 22.61073 | -23.93617 | 36.80121 | 0.515 | 29.00943 |

Abbreviations: LC, laparoscopic cholecystectomy; LCBDE, laparoscopic common bile duct exploration; LTCBDE, laparoscopic transcystic bile duct exploration; ERCP, endoscopic retrograde cholangiopancreatography; PS, primary suture; TTD, T-tube drainage.

**5.5 Intraoperative blood loss**

**Table S5.5:** Side-splitting of intraoperative blood loss in Cohort studies. Inconsistency test between direct and indirect treatment comparisons in mixed treatment comparison.

| Comparison | Direct | | Indirect | | Difference | | | tau |
| --- | --- | --- | --- | --- | --- | --- | --- | --- |
|  | Coef. | Std. Err. | Coef. | Std. Err. | Coef. | Std. Err. | P>\|z\| |  |
| LC + LCBDE + PS VS LC + ERCP (two-stage) | -4.464879 | 3.851914 | 4.790915 | 5.961802 | -9.255795 | 7.130635 | 0.194 | 4.782515 |
| LC + LCBDE + PS VS LC + LTCBDE | -16.70467 | 6.727315 | -16.82577 | 13.17455 | 0.1211015 | 14.61175 | 0.993 | 6.235899 |
| LC + LCBDE + PS VS LC + LCBDE + TTD | -1.377718 | 3.433415 | -11.87102 | 6.048929 | 10.4933 | 6.955492 | 0.131 | 4.650984 |
| LC + ERCP (one-stage) VS LC + ERCP (two-stage) | 5.1 | 6.243778 | -8.759653 | 200.3809 | 13.85965 | 200.5205 | 0.945 | 5.522914 |
| LC + ERCP (two-stage) VS LC + LTCBDE | -18.97652 | 7.36302 | -6.002149 | 11.2603 | -12.97437 | 13.82403 | 0.348 | 5.855947 |
| LC + ERCP (two-stage) VS LC + LCBDE (mixed) | 3.511096 | 16.53011 | -11.55001 | 8.327136 | 15.06111 | 18.49217 | 0.415 | 5.608558 |
| LC + ERCP (two-stage) VS LC + LCBDE + TTD | -7.189968 | 4.768361 | 3.314969 | 5.072938 | -10.50494 | 6.96219 | 0.131 | 4.652764 |
| LC + LTCBDE VS LC + LCBDE (mixed) | 4.99976 | 5.652577 | 20.40924 | 17.62976 | -15.40948 | 18.51362 | 0.405 | 5.611428 |

Abbreviations: LC, laparoscopic cholecystectomy; LCBDE, laparoscopic common bile duct exploration; LTCBDE, laparoscopic transcystic bile duct exploration; ERCP, endoscopic retrograde cholangiopancreatography; PS, primary suture; TTD, T-tube drainage.

**5.6 Total length of hospital**

**Table S5.6a:** Side-splitting of total length of hospital stay in RCTs. Inconsistency test between direct and indirect treatment comparisons in mixed treatment comparison.

| Comparison | Direct | | Indirect | | Difference | | | tau |
| --- | --- | --- | --- | --- | --- | --- | --- | --- |
|  | Coef. | Std. Err. | Coef. | Std. Err. | Coef. | Std. Err. | P>\|z\| |  |
| LC + LCBDE + PS VS LC + ERCP (two-stage) | 0.3570827 | 0.9241947 | -0.375845 | 1.783507 | 0.7329276 | 2.018164 | 0.716 | 1.528486 |
| LC + LCBDE + PS VS LC + LTCBDE | -4.2 | 1.453008 | -0.6626551 | 1.760933 | -3.537345 | 2.283006 | 0.121 | 1.404422 |
| LC + LCBDE + PS VS LC + LCBDE + TTD | 3.280773 | 0.7833315 | 0.110318 | 2.687725 | 3.170455 | 2.799526 | 0.257 | 1.495732 |
| LC + ERCP (one-stage) VS LC + ERCP (two-stage) | 2.395583 | 0.8431827 | 1.050148 | 1.957486 | 1.345435 | 2.132087 | 0.528 | 1.558675 |
| LC + ERCP (one-stage) VS LC + LCBDE (mixed) | -0.9000041 | 1.576267 | 0.4437738 | 1.436395 | -1.343778 | 2.132568 | 0.529 | 1.558706 |
| LC + ERCP (two-stage) VS LC + LTCBDE | -1.300026 | 1.562999 | -4.837367 | 1.66415 | 3.537341 | 2.283052 | 0.121 | 1.404426 |
| LC + ERCP (two-stage) VS LC + LCBDE (mixed) | -1.95152 | 1.161645 | -3.294685 | 1.787332 | 1.343166 | 2.132337 | 0.529 | 1.558688 |
| LC + ERCP (two-stage) VS LC + LCBDE + TTD | 0.2003198 | 2.552832 | 3.370479 | 1.149048 | -3.170159 | 2.799515 | 0.257 | 1.495741 |

Abbreviations: LC, laparoscopic cholecystectomy; LCBDE, laparoscopic common bile duct exploration; LTCBDE, laparoscopic transcystic bile duct exploration; ERCP, endoscopic retrograde cholangiopancreatography; PS, primary suture; TTD, T-tube drainage.

**Table S5.6b:** Side-splitting of total length of hospital stay in Cohort studies. Inconsistency test between direct and indirect treatment comparisons in mixed treatment comparison.

| Comparison | Direct | | Indirect | | Difference | | | tau |
| --- | --- | --- | --- | --- | --- | --- | --- | --- |
|  | Coef. | Std. Err. | Coef. | Std. Err. | Coef. | Std. Err. | P>\|z\| |  |
| LC + LCBDE + PS VS LC + ERCP (one-stage) | 2 | 2.22615 | -1.229416 | 1.256664 | 3.229416 | 2.556354 | 0.206 | 2.2202 |
| LC + LCBDE + PS VS LC + ERCP (two-stage) | 2.157173 | 1.058938 | 3.976746 | 1.359554 | -1.819574 | 1.723078 | 0.291 | 2.250778 |
| LC + LCBDE + PS VS LC + LTCBDE | -2.519522 | 1.593798 | -0.1178403 | 1.746603 | -2.401681 | 2.366399 | 0.31 | 2.238715 |
| LC + LCBDE + PS VS LC + LCBDE + TTD | 2.771497 | 0.9749513 | 0.8167254 | 1.887081 | 1.954772 | 2.121681 | 0.357 | 2.258882 |
| LC + ERCP (one-stage) VS LC + ERCP (two-stage) | 4.315616 | 1.070518 | 1.555279 | 1.39458 | 2.760337 | 1.768002 | 0.118 | 2.187475 |
| LC + ERCP (one-stage) VS LC + LCBDE (mixed) | -0.1940673 | 1.300703 | 2.149916 | 1.351702 | -2.343983 | 1.875627 | 0.211 | 2.231512 |
| LC + ERCP (two-stage) VS LC + LTCBDE | -4.272754 | 1.647194 | -4.269677 | 1.585755 | -0.0030763 | 2.287306 | 0.999 | 2.296113 |
| LC + ERCP (two-stage) VS LC + LCBDE (mixed) | -1.957384 | 0.8405998 | -3.72793 | 1.551587 | 1.770547 | 1.769194 | 0.317 | 2.253262 |
| LC + ERCP (two-stage) VS LC + LCBDE + TTD | -1.555355 | 1.366172 | 0.6019718 | 1.375991 | -2.157327 | 1.939089 | 0.266 | 2.241505 |
| LC + LTCBDE VS LC + LCBDE (mixed) | 1 | 2.283282 | 2.297039 | 1.491071 | -1.29704 | 2.727026 | 0.634 | 2.283034 |
| LC + LTCBDE VS LC + LCBDE + TTD | 4.489945 | 2.327363 | 3.465111 | 1.582975 | 1.024834 | 2.818121 | 0.716 | 2.287741 |

Abbreviations: LC, laparoscopic cholecystectomy; LCBDE, laparoscopic common bile duct exploration; LTCBDE, laparoscopic transcystic bile duct exploration; ERCP, endoscopic retrograde cholangiopancreatography; PS, primary suture; TTD, T-tube drainage.

**5.7 Conversion to open surgery**

**Table S5.7a:** Side-splitting of conversion to open surgery in RCTs. Inconsistency test between direct and indirect treatment comparisons in mixed treatment comparison.

| Comparison | Direct | | Indirect | | Difference | | | tau |
| --- | --- | --- | --- | --- | --- | --- | --- | --- |
|  | Coef. | Std. Err. | Coef. | Std. Err. | Coef. | Std. Err. | P>\|z\| |  |
| LC + LCBDE + PS VS LC + ERCP (two-stage) | 0.562882 | 0.6302981 | -0.404091 | 1.003168 | 0.966973 | 1.184745 | 0.414 | 6.45E-06 |
| LC + LCBDE + PS VS LC + LTCBDE | -0.2066143 | 1.424685 | 0.3507716 | 2.081893 | -0.5573858 | 2.522698 | 0.825 | 1.75E-06 |
| LC + LCBDE + PS VS LC + LCBDE + TTD | -0.1637642 | 0.6822322 | 0.7862719 | 1.056125 | -0.9500361 | 1.257315 | 0.45 | 0.0000246 |
| LC + ERCP (one-stage) VS LC + ERCP (two-stage) | 0.0846201 | 0.4931322 | -0.662471 | 0.9662083 | 0.7470911 | 1.084802 | 0.491 | 4.80E-08 |
| LC + ERCP (one-stage) VS LC + LCBDE (mixed) | -0.2815199 | 0.723667 | 0.4188947 | 0.7718269 | -0.7004146 | 1.058019 | 0.508 | 7.13E-09 |
| LC + ERCP (two-stage) VS LC + LTCBDE | 0.0354093 | 2.008732 | -0.5219965 | 1.525952 | 0.5574058 | 2.522535 | 0.825 | 2.12E-06 |
| LC + ERCP (two-stage) VS LC + LCBDE (mixed) | 0.3095521 | 0.558112 | -0.41319 | 0.9226209 | 0.7227422 | 1.07828 | 0.503 | 0.0000119 |
| LC + ERCP (two-stage) VS LC + LCBDE + TTD | 0.2731005 | 0.8617848 | -0.676978 | 0.9155155 | 0.9500785 | 1.257313 | 0.45 | 4.90E-06 |

Abbreviations: LC, laparoscopic cholecystectomy; LCBDE, laparoscopic common bile duct exploration; LTCBDE, laparoscopic transcystic bile duct exploration; ERCP, endoscopic retrograde cholangiopancreatography; PS, primary suture; TTD, T-tube drainage.

**Table S5.7b:** Side-splitting of conversion to open surgery in Cohort studies. Inconsistency test between direct and indirect treatment comparisons in mixed treatment comparison.

| Comparison | Direct | | Indirect | | Difference | | | tau |
| --- | --- | --- | --- | --- | --- | --- | --- | --- |
|  | Coef. | Std. Err. | Coef. | Std. Err. | Coef. | Std. Err. | P>\|z\| |  |
| LC + LCBDE + PS VS LC + ERCP (one-stage) | 0.0096155 | 2.004802 | -0.2383023 | 0.5690175 | 0.2479178 | 2.083989 | 0.905 | 6.46E-08 |
| LC + LCBDE + PS VS LC + ERCP (two-stage) | 0.0666822 | 0.424356 | 0.4762766 | 0.9442296 | -0.4095945 | 1.035212 | 0.692 | 1.22E-07 |
| LC + LCBDE + PS VS LC + LTCBDE | 0.1386006 | 0.7254449 | -0.7791294 | 1.711601 | 0.91773 | 2.01017 | 0.648 | 3.71E-07 |
| LC + LCBDE + PS VS LC + LCBDE + TTD | 0.2235738 | 0.6311865 | 0.0062984 | 0.9212301 | 0.2172754 | 1.120112 | 0.846 | 5.09E-07 |
| LC + ERCP (one-stage) VS LC + ERCP (two-stage) | 0.4569608 | 0.6454838 | 0.2940164 | 0.5046169 | 0.1629444 | 0.8092092 | 0.84 | 4.89E-07 |
| LC + ERCP (one-stage) VS LC + LCBDE (mixed) | -0.1953163 | 0.3740978 | 0.0126276 | 0.7600042 | -0.2079439 | 0.8167331 | 0.799 | 2.13E-06 |
| LC + ERCP (two-stage) VS LC + LTCBDE | -0.0179806 | 2.001798 | -0.2027605 | 0.6954467 | 0.1847799 | 2.119161 | 0.931 | 7.43E-07 |
| LC + ERCP (two-stage) VS LC + LCBDE (mixed) | -0.6092555 | 0.29529 | 0.2390527 | 0.8305189 | -0.8483083 | 0.8803281 | 0.335 | 5.98E-07 |
| LC + ERCP (two-stage) VS LC + LCBDE + TTD | -0.1525846 | 0.7842102 | 0.1804123 | 0.7633024 | -0.3329969 | 1.094344 | 0.761 | 8.88E-07 |
| LC + LTCBDE VS LC + LCBDE + TTD | 0.3500236 | 0.6638354 | -0.4212105 | 1.485464 | 0.7712341 | 1.695559 | 0.649 | 3.70E-08 |

Abbreviations: LC, laparoscopic cholecystectomy; LCBDE, laparoscopic common bile duct exploration; LTCBDE, laparoscopic transcystic bile duct exploration; ERCP, endoscopic retrograde cholangiopancreatography; PS, primary suture; TTD, T-tube drainage.

**5.8 Reintervention**

**Table S5.8a:** Side-splitting of reintervention in RCTs. Inconsistency test between direct and indirect treatment comparisons in mixed treatment comparison.

| Comparison | Direct | | Indirect | | Difference | | | tau |
| --- | --- | --- | --- | --- | --- | --- | --- | --- |
|  | Coef. | Std. Err. | Coef. | Std. Err. | Coef. | Std. Err. | P>\|z\| |  |
| LC + LCBDE + PS VS LC + ERCP (two-stage) | 1.200738 | 0.5802187 | 0.6967799 | 35.35523 | 0.5039582 | 35.35999 | 0.989 | 1.39E-09 |
| LC + ERCP (one-stage) VS LC + ERCP (two-stage) | 0.8824815 | 0.5296384 | 1.178019 | 1.195262 | -0.2955379 | 1.295179 | 0.82 | 1.16E-07 |
| LC + ERCP (one-stage) VS LC + LCBDE (mixed) | -0.0079119 | 0.8246384 | -0.7273721 | 0.9097674 | 0.7194602 | 1.227874 | 0.558 | 1.39E-09 |
| LC + ERCP (two-stage) VS LC + LTCBDE | 0.748207 | 1.239647 | -1.65308 | 199.9519 | 2.401287 | 199.9481 | 0.99 | 9.78E-09 |
| LC + ERCP (two-stage) VS LC + LCBDE (mixed) | -1.439851 | 0.5731299 | -0.5046896 | 1.150218 | -0.935161 | 1.26793 | 0.461 | 1.03E-09 |

Abbreviations: LC, laparoscopic cholecystectomy; LCBDE, laparoscopic common bile duct exploration; LTCBDE, laparoscopic transcystic bile duct exploration; ERCP, endoscopic retrograde cholangiopancreatography; PS, primary suture; TTD, T-tube drainage.

**Table S5.8b:** Side-splitting of reintervention in Cohort studies. Inconsistency test between direct and indirect treatment comparisons in mixed treatment comparison.

| Comparison | Direct | | Indirect | | Difference | | | tau |
| --- | --- | --- | --- | --- | --- | --- | --- | --- |
|  | Coef. | Std. Err. | Coef. | Std. Err. | Coef. | Std. Err. | P>\|z\| |  |
| LC + LCBDE + PS VS LC + ERCP (two-stage) | 0.7489234 | 0.4361569 | 0.9085484 | 1.059353 | -0.159625 | 1.14793 | 0.889 | 0.6411792 |
| LC + LCBDE + PS VS LC + LTCBDE | -0.5048424 | 0.6979808 | -0.0403643 | 1.054453 | -0.4644781 | 1.251739 | 0.711 | 0.6556592 |
| LC + LCBDE + PS VS LC + LCBDE + TTD | -0.0584551 | 0.7253607 | -0.2970643 | 1.207092 | 0.2386092 | 1.377111 | 0.862 | 0.640156 |
| LC + ERCP (one-stage) VS LC + ERCP (two-stage) | 0.1882595 | 0.5589165 | 1.117828 | 1.329605 | -0.9295688 | 1.397446 | 0.506 | 0.6279997 |
| LC + ERCP (one-stage) VS LC + LCBDE (mixed) | -0.8528072 | 0.8339213 | -1.159701 | 0.9892059 | 0.3068943 | 1.22571 | 0.802 | 0.6477355 |
| LC + ERCP (two-stage) VS LC + LTCBDE | -1.424806 | 0.8817491 | -0.9043395 | 0.7996177 | -0.5204662 | 1.170557 | 0.657 | 0.6606584 |
| LC + ERCP (two-stage) VS LC + LCBDE (mixed) | -1.465482 | 0.5188161 | -0.7710504 | 1.018626 | -0.694432 | 1.175079 | 0.555 | 0.5586706 |
| LC + ERCP (two-stage) VS LC + LCBDE + TTD | -0.941994 | 1.276456 | -0.8683797 | 0.8085237 | -0.0736143 | 1.51097 | 0.961 | 0.6354006 |
| LC + LTCBDE VS LC + LCBDE (mixed) | -0.7133567 | 1.399468 | 0.0189919 | 0.8393721 | -0.7323487 | 1.631886 | 0.654 | 0.646655 |
| LC + LTCBDE VS LC + LCBDE + TTD | -0.3125364 | 1.165482 | 0.6889617 | 1.02178 | -1.001498 | 1.552531 | 0.519 | 0.616215 |

Abbreviations: LC, laparoscopic cholecystectomy; LCBDE, laparoscopic common bile duct exploration; LTCBDE, laparoscopic transcystic bile duct exploration; ERCP, endoscopic retrograde cholangiopancreatography; PS, primary suture; TTD, T-tube drainage.

**5.9 Postoperative bile leakage**

**Table S5.9a:** Side-splitting of postoperative bile leakage in RCTs. Inconsistency test between direct and indirect treatment comparisons in mixed treatment comparison.

| Comparison | Direct | | Indirect | | Difference | | | tau |
| --- | --- | --- | --- | --- | --- | --- | --- | --- |
|  | Coef. | Std. Err. | Coef. | Std. Err. | Coef. | Std. Err. | P>\|z\| |  |
| LC + LCBDE + PS VS LC + ERCP (two-stage) | -0.6516739 | 0.6807203 | -1.868492 | 1.184169 | 1.216819 | 1.365883 | 0.373 | 1.83E-09 |
| LC + LCBDE + PS VS LC + LCBDE + TTD | -0.1802063 | 0.4071386 | 1.039397 | 1.304465 | -1.219603 | 1.366525 | 0.372 | 7.27E-10 |
| LC + ERCP (one-stage) VS LC + ERCP (two-stage) | -0.0101489 | 0.7135131 | -0.5274304 | 1.328524 | 0.5172815 | 1.508082 | 0.732 | 8.07E-10 |
| LC + ERCP (one-stage) VS LC + LCBDE (mixed) | 1.622352 | 0.8875553 | 2.088731 | 1.086383 | -0.4663796 | 1.403382 | 0.74 | 9.55E-10 |
| LC + ERCP (two-stage) VS LC + LTCBDE | 0.0354019 | 2.008833 | 1.943275 | 200.0281 | -1.907873 | 200.018 | 0.992 | 6.94E-10 |
| LC + ERCP (two-stage) VS LC + LCBDE (mixed) | 2.023604 | 0.6869424 | 1.612267 | 1.328806 | 0.4113367 | 1.507426 | 0.785 | 1.25E-09 |
| LC + ERCP (two-stage) VS LC + LCBDE + TTD | 1.690647 | 1.112828 | 0.4712236 | 0.7930737 | 1.219424 | 1.366524 | 0.372 | 7.14E-11 |

Abbreviations: LC, laparoscopic cholecystectomy; LCBDE, laparoscopic common bile duct exploration; LTCBDE, laparoscopic transcystic bile duct exploration; ERCP, endoscopic retrograde cholangiopancreatography; PS, primary suture; TTD, T-tube drainage.

**Table S5.9b:** Side-splitting of postoperative bile leakage in Cohort studies. Inconsistency test between direct and indirect treatment comparisons in mixed treatment comparison.

| Comparison | Direct | | Indirect | | Difference | | | tau |
| --- | --- | --- | --- | --- | --- | --- | --- | --- |
|  | Coef. | Std. Err. | Coef. | Std. Err. | Coef. | Std. Err. | P>\|z\| |  |
| LC + LCBDE + PS VS LC + ERCP (one-stage) | 1.638566 | 1.555449 | -0.248979 | 0.6324006 | 1.887545 | 1.679093 | 0.261 | 2.50E-06 |
| LC + LCBDE + PS VS LC + ERCP (two-stage) | -1.211566 | 0.5415676 | -0.9307819 | 0.7639011 | -0.2807846 | 0.9440919 | 0.766 | 0.1210917 |
| LC + LCBDE + PS VS LC + LTCBDE | -0.429275 | 0.5507241 | -2.201396 | 0.8465749 | 1.772121 | 1.024185 | 0.084 | 5.47E-10 |
| LC + LCBDE + PS VS LC + LCBDE + TTD | 0.3904159 | 0.4212623 | 1.659323 | 1.033654 | -1.268908 | 1.13405 | 0.263 | 1.09E-09 |
| LC + ERCP (one-stage) VS LC + ERCP (two-stage) | -0.7583023 | 0.4918514 | -2.479247 | 0.8986543 | 1.720944 | 1.003948 | 0.086 | 1.78E-06 |
| LC + ERCP (one-stage) VS LC + LCBDE (mixed) | -0.5454457 | 0.8031267 | 0.794027 | 0.6735273 | -1.339473 | 1.043241 | 0.199 | 1.63E-07 |
| LC + ERCP (two-stage) VS LC + LTCBDE | 0.9031068 | 0.8736769 | -0.3370247 | 0.6921454 | 1.240131 | 1.15511 | 0.283 | 3.48E-07 |
| LC + ERCP (two-stage) VS LC + LCBDE (mixed) | 1.26113 | 0.4319347 | 1.815105 | 0.8462954 | -0.553975 | 0.9569464 | 0.563 | 1.64E-06 |
| LC + ERCP (two-stage) VS LC + LCBDE + TTD | 2.185654 | 1.090222 | 1.561577 | 0.5885517 | 0.6240772 | 1.238945 | 0.614 | 0.0000152 |
| LC + LTCBDE VS LC + LCBDE (mixed) | 2.073658 | 1.088829 | 0.8725036 | 0.7045061 | 1.201155 | 1.29687 | 0.354 | 1.11E-09 |
| LC + LTCBDE VS LC + LCBDE + TTD | 2.254739 | 0.746985 | 0.9402722 | 0.6967493 | 1.314467 | 1.017505 | 0.196 | 8.57E-08 |

Abbreviations: LC, laparoscopic cholecystectomy; LCBDE, laparoscopic common bile duct exploration; LTCBDE, laparoscopic transcystic bile duct exploration; ERCP, endoscopic retrograde cholangiopancreatography; PS, primary suture; TTD, T-tube drainage.

**5.10 Postoperative pancreatitis**

**Table S5.10a:** Side-splitting of postoperative pancreatitis in RCTs. Inconsistency test between direct and indirect treatment comparisons in mixed treatment comparison.

| Comparison | Direct | | Indirect | | Difference | | | tau |
| --- | --- | --- | --- | --- | --- | --- | --- | --- |
|  | Coef. | Std. Err. | Coef. | Std. Err. | Coef. | Std. Err. | P>\|z\| |  |
| LC + LCBDE + PS VS LC + ERCP (two-stage) | 2.075193 | 0.8875377 | 0.6188835 | 1.512261 | 1.456309 | 1.75347 | 0.406 | 1.17E-10 |
| LC + LCBDE + PS VS LC + LCBDE + TTD | -0.6408485 | 0.9021587 | 0.8128905 | 1.505275 | -1.453739 | 1.75492 | 0.407 | 2.50E-08 |
| LC + ERCP (one-stage) VS LC + ERCP (two-stage) | 0.7622268 | 0.6505258 | -0.6152685 | 1.125977 | 1.377495 | 1.284327 | 0.283 | 1.47E-07 |
| LC + ERCP (one-stage) VS LC + LCBDE (mixed) | -1.096103 | 0.9827104 | 0.0092737 | 0.7746129 | -1.105376 | 1.251326 | 0.377 | 1.17E-06 |
| LC + ERCP (two-stage) VS LC + LTCBDE | 0.0354019 | 2.008833 | -3.368971 | 200.031 | 3.404373 | 200.0209 | 0.986 | 0.000025 |
| LC + ERCP (two-stage) VS LC + LCBDE (mixed) | -0.7668988 | 0.3304942 | -2.03939 | 1.314013 | 1.272491 | 1.352394 | 0.347 | 1.78E-08 |
| LC + ERCP (two-stage) VS LC + LCBDE + TTD | -1.260508 | 1.216019 | -2.714776 | 1.265328 | 1.454268 | 1.754923 | 0.407 | 1.33E-08 |

Abbreviations: LC, laparoscopic cholecystectomy; LCBDE, laparoscopic common bile duct exploration; LTCBDE, laparoscopic transcystic bile duct exploration; ERCP, endoscopic retrograde cholangiopancreatography; PS, primary suture; TTD, T-tube drainage.

**Table S5.10b:** Side-splitting of postoperative pancreatitis in Cohort studies. Inconsistency test between direct and indirect treatment comparisons in mixed treatment comparison.

| Comparison | Direct | | Indirect | | Difference | | | tau |
| --- | --- | --- | --- | --- | --- | --- | --- | --- |
|  | Coef. | Std. Err. | Coef. | Std. Err. | Coef. | Std. Err. | P>\|z\| |  |
| LC + LCBDE + PS VS LC + ERCP (one-stage) | -2.547655 | 0.8656456 | 1.596666 | 0.5275336 | -4.144321 | 1.013723 | 0 | 0.4239977 |
| LC + LCBDE + PS VS LC + ERCP (two-stage) | 1.730132 | 0.4526086 | -0.8860494 | 0.8219534 | 2.616182 | 0.9297675 | 0.005 | 0.6182982 |
| LC + LCBDE + PS VS LC + LTCBDE | -1.135626 | 1.164352 | -0.9291591 | 1.127379 | -0.2064672 | 1.588349 | 0.897 | 0.957111 |
| LC + LCBDE + PS VS LC + LCBDE + TTD | -0.4497937 | 1.056559 | -0.6006441 | 1.263056 | 0.1508504 | 1.623242 | 0.926 | 0.9478947 |
| LC + ERCP (one-stage) VS LC + ERCP (two-stage) | -0.0294721 | 0.5220036 | 1.969461 | 0.7467575 | -1.998933 | 0.9027145 | 0.027 | 0.7196417 |
| LC + ERCP (one-stage) VS LC + LCBDE (mixed) | -1.908117 | 0.7995786 | -0.990894 | 0.8362027 | -0.9172227 | 1.158747 | 0.429 | 0.9629768 |
| LC + ERCP (two-stage) VS LC + LTCBDE | -1.582445 | 1.075385 | -2.674775 | 1.035392 | 1.09233 | 1.445427 | 0.45 | 0.9495232 |
| LC + ERCP (two-stage) VS LC + LCBDE (mixed) | -2.232029 | 0.5266913 | -1.571553 | 0.948777 | -0.6604764 | 1.083949 | 0.542 | 0.9348555 |
| LC + ERCP (two-stage) VS LC + LCBDE + TTD | -1.767563 | 1.0226 | -1.432092 | 1.223141 | -0.335471 | 1.568838 | 0.831 | 0.945564 |
| LC + LTCBDE VS LC + LCBDE (mixed) | 1.139332 | 1.497067 | -0.3924812 | 0.9883213 | 1.531814 | 1.793882 | 0.393 | 0.9311765 |
| LC + LTCBDE VS LC + LCBDE + TTD | 0.728267 | 1.846687 | 0.4245436 | 1.241451 | 0.3037234 | 2.233639 | 0.892 | 0.946427 |

Abbreviations: LC, laparoscopic cholecystectomy; LCBDE, laparoscopic common bile duct exploration; LTCBDE, laparoscopic transcystic bile duct exploration; ERCP, endoscopic retrograde cholangiopancreatography; PS, primary suture; TTD, T-tube drainage.

**5.11 Postoperative biliary bleeding**

**Table S5.11a:** Side-splitting of postoperative biliary bleeding in RCTs. Inconsistency test between direct and indirect treatment comparisons in mixed treatment comparison.

| Comparison | Direct | | Indirect | | Difference | | | tau |
| --- | --- | --- | --- | --- | --- | --- | --- | --- |
|  | Coef. | Std. Err. | Coef. | Std. Err. | Coef. | Std. Err. | P>\|z\| |  |
| LC + LCBDE + PS VS LC + ERCP (two-stage) | 1.112898 | 1.641718 | 2.032522 | 1.529227 | -0.9196237 | 2.243608 | 0.682 | 1.18E-09 |
| LC + LCBDE + PS VS LC + LCBDE + TTD | 0.9055016 | 0.9863295 | -0.0209934 | 2.01478 | 0.9264951 | 2.243253 | 0.68 | 3.99E-10 |
| LC + ERCP (one-stage) VS LC + ERCP (two-stage) | -0.0250353 | 0.7132573 | 0.5223218 | 1.891026 | -0.5473571 | 2.021158 | 0.787 | 1.32E-09 |
| LC + ERCP (one-stage) VS LC + LCBDE (mixed) | -1.461009 | 1.126356 | -2.007023 | 1.678808 | 0.546013 | 2.021623 | 0.787 | 5.82E-10 |
| LC + ERCP (two-stage) VS LC + LTCBDE | 0.0354019 | 2.008833 | -3.175587 | 200.0205 | 3.210989 | 200.0104 | 0.987 | 2.47E-09 |
| LC + ERCP (two-stage) VS LC + LCBDE (mixed) | -1.981842 | 1.519765 | -1.436504 | 1.333143 | -0.5453381 | 2.021571 | 0.787 | 1.02E-10 |
| LC + ERCP (two-stage) VS LC + LCBDE + TTD | -1.131041 | 1.170695 | -0.2050886 | 1.913861 | -0.9259523 | 2.243575 | 0.68 | 9.61E-10 |

Abbreviations: LC, laparoscopic cholecystectomy; LCBDE, laparoscopic common bile duct exploration; LTCBDE, laparoscopic transcystic bile duct exploration; ERCP, endoscopic retrograde cholangiopancreatography; PS, primary suture; TTD, T-tube drainage.

**Table S5.11b:** Side-splitting of postoperative biliary bleeding in Cohort studies. Inconsistency test between direct and indirect treatment comparisons in mixed treatment comparison.

| Comparison | Direct | | Indirect | | Difference | | | tau |
| --- | --- | --- | --- | --- | --- | --- | --- | --- |
|  | Coef. | Std. Err. | Coef. | Std. Err. | Coef. | Std. Err. | P>\|z\| |  |
| LC + LCBDE + PS VS LC + ERCP (one-stage) | -2.226638 | 1.497276 | 0.9199469 | 1.069368 | -3.146585 | 1.839941 | 0.087 | 2.55E-09 |
| LC + LCBDE + PS VS LC + ERCP (two-stage) | 0.7007723 | 0.5946841 | -0.2215589 | 1.191922 | 0.9223312 | 1.33208 | 0.489 | 1.27E-08 |
| LC + LCBDE + PS VS LC + LTCBDE | -1.04154 | 1.388844 | -0.4220889 | 1.770101 | -0.6194513 | 1.889016 | 0.743 | 2.71E-10 |
| LC + LCBDE + PS VS LC + LCBDE + TTD | 0.8621501 | 0.8058658 | -0.9373146 | 1.619829 | 1.799465 | 1.809217 | 0.32 | 8.04E-09 |
| LC + ERCP (one-stage) VS LC + ERCP (two-stage) | 0.1676204 | 1.074271 | 1.246486 | 1.172759 | -1.078865 | 1.590453 | 0.498 | 6.21E-09 |
| LC + ERCP (one-stage) VS LC + LCBDE (mixed) | -0.8820223 | 1.645041 | 1.017185 | 1.034431 | -1.899208 | 1.943258 | 0.328 | 1.62E-09 |
| LC + ERCP (two-stage) VS LC + LTCBDE | -1.340717 | 1.212851 | -1.493915 | 2.293213 | 0.153198 | 2.128469 | 0.943 | 1.76E-11 |
| LC + ERCP (two-stage) VS LC + LCBDE (mixed) | -0.0424331 | 0.5246467 | -1.941408 | 1.870827 | 1.898975 | 1.942953 | 0.328 | 2.18E-10 |
| LC + ERCP (two-stage) VS LC + LCBDE + TTD | -1.281971 | 1.520072 | 0.5173178 | 0.9810804 | -1.799289 | 1.809171 | 0.32 | 8.69E-09 |

Abbreviations: LC, laparoscopic cholecystectomy; LCBDE, laparoscopic common bile duct exploration; LTCBDE, laparoscopic transcystic bile duct exploration; ERCP, endoscopic retrograde cholangiopancreatography; PS, primary suture; TTD, T-tube drainage.

**5.12 Postoperative surgical site infection**

**Table S5.12a:** Side-splitting of postoperative surgical site infection in RCTs. Inconsistency test between direct and indirect treatment comparisons in mixed treatment comparison.

| Comparison | Direct | | Indirect | | Difference | | | tau |
| --- | --- | --- | --- | --- | --- | --- | --- | --- |
|  | Coef. | Std. Err. | Coef. | Std. Err. | Coef. | Std. Err. | P>\|z\| |  |
| LC + LCBDE + PS VS LC + ERCP (two-stage) | 0.4090832 | 0.9367585 | 0.0305323 | 1.104224 | 0.3785508 | 1.448042 | 0.794 | 3.01E-09 |
| LC + LCBDE + PS VS LC + LCBDE + TTD | 0.0353452 | 0.8243425 | 0.4137727 | 1.19074 | -0.3784275 | 1.448241 | 0.794 | 2.57E-10 |
| LC + ERCP (one-stage) VS LC + ERCP (two-stage) | 0.0013832 | 1.005663 | 0.6560078 | 1.369994 | -0.6546247 | 1.699566 | 0.7 | 5.47E-10 |
| LC + ERCP (one-stage) VS LC + LCBDE (mixed) | 0.6561 | 1.232261 | 0.0014681 | 1.170509 | 0.6546319 | 1.699591 | 0.7 | 1.36E-10 |
| LC + ERCP (two-stage) VS LC + LTCBDE | 0.0354019 | 2.008833 | -0.4659444 | 200.0254 | 0.5013463 | 200.0153 | 0.998 | 1.83E-10 |
| LC + ERCP (two-stage) VS LC + LCBDE (mixed) | 0.0000104 | 0.599142 | 0.654537 | 1.590406 | -0.6545267 | 1.699505 | 0.7 | 5.70E-10 |
| LC + ERCP (two-stage) VS LC + LCBDE + TTD | 0.0049075 | 0.7354896 | -0.3735993 | 1.247635 | 0.3785068 | 1.448292 | 0.794 | 1.52E-09 |

Abbreviations: LC, laparoscopic cholecystectomy; LCBDE, laparoscopic common bile duct exploration; LTCBDE, laparoscopic transcystic bile duct exploration; ERCP, endoscopic retrograde cholangiopancreatography; PS, primary suture; TTD, T-tube drainage.

**Table S5.12b:** Side-splitting of postoperative surgical site infection in Cohort studies. Inconsistency test between direct and indirect treatment comparisons in mixed treatment comparison.

| Comparison | Direct | | Indirect | | Difference | | | tau |
| --- | --- | --- | --- | --- | --- | --- | --- | --- |
|  | Coef. | Std. Err. | Coef. | Std. Err. | Coef. | Std. Err. | P>\|z\| |  |
| LC + LCBDE + PS VS LC + ERCP (two-stage) | -1.857539 | 1.554605 | -0.1108347 | 1.16971 | -1.746704 | 1.890927 | 0.356 | 6.16E-10 |
| LC + LCBDE + PS VS LC + LTCBDE | -0.0758677 | 1.055043 | -2.269662 | 1.46881 | 2.193794 | 1.964269 | 0.264 | 2.47E-09 |
| LC + LCBDE + PS VS LC + LCBDE + TTD | 0.2380283 | 0.6012353 | -0.5283397 | 3.51424 | 0.766368 | 3.527322 | 0.828 | 7.17E-11 |
| LC + ERCP (one-stage) VS LC + ERCP (two-stage) | 0.0138759 | 0.9558093 | -1.470288 | 141.4512 | 1.484164 | 141.4609 | 0.992 | 5.95E-10 |
| LC + ERCP (two-stage) VS LC + LTCBDE | -0.697889 | 1.23516 | 0.4077097 | 1.241261 | -1.105599 | 1.751128 | 0.528 | 1.83E-09 |
| LC + ERCP (two-stage) VS LC + LCBDE (mixed) | 0.1075602 | 1.005501 | 1.563968 | 141.4296 | -1.456408 | 141.426 | 0.992 | 2.08E-11 |
| LC + ERCP (two-stage) VS LC + LCBDE + TTD | 1.05376 | 1.019541 | 0.779596 | 1.287291 | 0.2741643 | 1.545452 | 0.859 | 3.90E-09 |
| LC + LTCBDE VS LC + LCBDE + TTD | 1.116672 | 0.7471805 | 1.044762 | 1.392046 | 0.0719102 | 1.60625 | 0.964 | 5.55E-10 |

Abbreviations: LC, laparoscopic cholecystectomy; LCBDE, laparoscopic common bile duct exploration; LTCBDE, laparoscopic transcystic bile duct exploration; ERCP, endoscopic retrograde cholangiopancreatography; PS, primary suture; TTD, T-tube drainage.

**5.13 Postoperative intra-abdominal infection**

**Table S5.13a:** Side-splitting of postoperative intra-abdominal infection in RCTs. Inconsistency test between direct and indirect treatment comparisons in mixed treatment comparison.

| Comparison | Direct | | Indirect | | Difference | | | tau |
| --- | --- | --- | --- | --- | --- | --- | --- | --- |
|  | Coef. | Std. Err. | Coef. | Std. Err. | Coef. | Std. Err. | P>\|z\| |  |
| LC + LCBDE + PS VS LC + ERCP (two-stage) | -0.0674413 | 1.430033 | 0.4167592 | 2.13851 | -0.4842004 | 2.572591 | 0.851 | 1.04E-09 |
| LC + LCBDE + PS VS LC + LCBDE + TTD | 0.4171292 | 0.6774067 | -0.0669138 | 2.484171 | 0.484043 | 2.574876 | 0.851 | 5.06E-08 |
| LC + ERCP (one-stage) VS LC + ERCP (two-stage) | 0.258832 | 0.7961987 | 2.693069 | 2.259277 | -2.434237 | 2.395569 | 0.31 | 7.47E-10 |
| LC + ERCP (one-stage) VS LC + LCBDE (mixed) | 1.061891 | 1.638523 | -1.374336 | 1.748633 | 2.436227 | 2.396296 | 0.309 | 3.39E-09 |
| LC + ERCP (two-stage) VS LC + LTCBDE | 0.0354019 | 2.008833 | -0.1289688 | 200.0131 | 0.1643707 | 200.003 | 0.999 | 2.03E-08 |
| LC + ERCP (two-stage) VS LC + LCBDE (mixed) | -1.633234 | 1.556859 | 0.8029434 | 1.821665 | -2.436177 | 2.39622 | 0.309 | 6.62E-09 |
| LC + ERCP (two-stage) VS LC + LCBDE + TTD | 0.0000361 | 2.031897 | 0.4840705 | 1.581552 | -0.4840343 | 2.574925 | 0.851 | 3.92E-08 |

Abbreviations: LC, laparoscopic cholecystectomy; LCBDE, laparoscopic common bile duct exploration; LTCBDE, laparoscopic transcystic bile duct exploration; ERCP, endoscopic retrograde cholangiopancreatography; PS, primary suture; TTD, T-tube drainage.

**Table S5.13b:** Side-splitting of postoperative intra-abdominal infection in Cohort studies. Inconsistency test between direct and indirect treatment comparisons in mixed treatment comparison.

| Comparison | Direct | | Indirect | | Difference | | | tau |
| --- | --- | --- | --- | --- | --- | --- | --- | --- |
|  | Coef. | Std. Err. | Coef. | Std. Err. | Coef. | Std. Err. | P>\|z\| |  |
| LC + LCBDE + PS VS LC + LTCBDE | -1.609465 | 2.008284 | -4.093262 | 3.108256 | 2.483796 | 3.705125 | 0.503 | 0.0891107 |
| LC + LCBDE + PS VS LC + LCBDE + TTD | 0.7843789 | 0.8390499 | 2.748311 | 81.80082 | -1.963932 | 81.81076 | 0.981 | 0.0000868 |
| LC + ERCP (one-stage) VS LC + ERCP (two-stage) | -1.305572 | 2.025852 | -1.013829 | 1.721055 | -0.291743 | 2.658331 | 0.913 | 0.1297528 |
| LC + ERCP (one-stage) VS LC + LCBDE (mixed) | -0.8821098 | 1.650151 | -1.173966 | 2.083848 | 0.2918565 | 2.658183 | 0.913 | 0.1297559 |
| LC + ERCP (two-stage) VS LC + LTCBDE | -0.9890663 | 1.558397 | -1.983164 | 43.76397 | 0.9940973 | 43.79436 | 0.982 | 0.0001901 |
| LC + ERCP (two-stage) VS LC + LCBDE (mixed) | 0.1315723 | 0.4897414 | 0.4247188 | 2.611661 | -0.2931465 | 2.657137 | 0.912 | 0.1296368 |
| LC + LTCBDE VS LC + LCBDE + TTD | 3.285063 | 1.559833 | 0.7919041 | 3.797246 | 2.493159 | 3.703007 | 0.501 | 0.0887956 |

Abbreviations: LC, laparoscopic cholecystectomy; LCBDE, laparoscopic common bile duct exploration; LTCBDE, laparoscopic transcystic bile duct exploration; ERCP, endoscopic retrograde cholangiopancreatography; PS, primary suture; TTD, T-tube drainage.

**5.14 Postoperative cholangitis**

**Table S5.14a:** Side-splitting of postoperative cholangitis in RCTs. Inconsistency test between direct and indirect treatment comparisons in mixed treatment comparison.

| Comparison | Direct | | Indirect | | Difference | | | tau |
| --- | --- | --- | --- | --- | --- | --- | --- | --- |
|  | Coef. | Std. Err. | Coef. | Std. Err. | Coef. | Std. Err. | P>\|z\| |  |
| LC + LCBDE + PS VS LC + ERCP (two-stage) | 1.638217 | 1.558455 | 0.5388141 | 1.310697 | 1.099403 | 2.036347 | 0.589 | 6.78E-08 |
| LC + LCBDE + PS VS LC + LTCBDE | -0.2886958 | 0.3476858 | 1.450806 | 2.315821 | -1.739502 | 2.341776 | 0.458 | 8.36E-10 |
| LC + LCBDE + PS VS LC + LCBDE + TTD | -8.45E-10 | 1.421431 | -0.235558 | 1.563497 | 0.235558 | 2.113052 | 0.911 | 2.51E-06 |
| LC + ERCP (one-stage) VS LC + ERCP (two-stage) | -0.429881 | 0.9363785 | 2.419019 | 141.4229 | -2.8489 | 141.4322 | 0.984 | 2.44E-09 |
| LC + ERCP (two-stage) VS LC + LTCBDE | 0.035512 | 2.008732 | -1.704255 | 1.203597 | 1.739767 | 2.341771 | 0.458 | 3.95E-10 |
| LC + ERCP (two-stage) VS LC + LCBDE (mixed) | -1.110517 | 1.640267 | -3.099281 | 200.0205 | 1.988764 | 200.0138 | 0.992 | 9.13E-08 |
| LC + ERCP (two-stage) VS LC + LCBDE + TTD | -1.148946 | 0.9554674 | -0.913812 | 1.884804 | -0.2351337 | 2.11314 | 0.911 | 3.28E-06 |

Abbreviations: LC, laparoscopic cholecystectomy; LCBDE, laparoscopic common bile duct exploration; LTCBDE, laparoscopic transcystic bile duct exploration; ERCP, endoscopic retrograde cholangiopancreatography; PS, primary suture; TTD, T-tube drainage.

**Table S5.14b:** Side-splitting of postoperative cholangitis in Cohort studies. Inconsistency test between direct and indirect treatment comparisons in mixed treatment comparison.

| Comparison | Direct | | Indirect | | Difference | | | tau |
| --- | --- | --- | --- | --- | --- | --- | --- | --- |
|  | Coef. | Std. Err. | Coef. | Std. Err. | Coef. | Std. Err. | P>\|z\| |  |
| LC + LCBDE + PS VS LC + ERCP (one-stage) | -2.437309 | 1.483755 | 1.569638 | 1.117369 | -4.006947 | 1.857429 | 0.031 | 4.15E-10 |
| LC + LCBDE + PS VS LC + ERCP (two-stage) | 0.9388525 | 0.4203201 | -0.3406567 | 1.400374 | 1.279509 | 1.462093 | 0.382 | 1.91E-10 |
| LC + LCBDE + PS VS LC + LTCBDE | -1.416016 | 1.51373 | -0.6532957 | 2.085732 | -0.7627204 | 1.791689 | 0.67 | 2.90E-07 |
| LC + LCBDE + PS VS LC + LCBDE + TTD | 1.459573 | 1.119753 | -0.0771582 | 1.491776 | 1.536732 | 1.924117 | 0.424 | 1.29E-09 |
| LC + ERCP (one-stage) VS LC + ERCP (two-stage) | 0.0371083 | 1.013636 | 1.765532 | 1.197313 | -1.728423 | 1.367028 | 0.206 | 1.92E-09 |
| LC + ERCP (one-stage) VS LC + LCBDE (mixed) | -1.16446 | 1.283371 | -0.1815523 | 1.077568 | -0.9829079 | 1.478571 | 0.506 | 2.38E-10 |
| LC + ERCP (two-stage) VS LC + LTCBDE | -1.974845 | 1.514249 | -2.737566 | 2.084602 | 0.7627204 | 1.791689 | 0.67 | 2.92E-07 |
| LC + ERCP (two-stage) VS LC + LCBDE (mixed) | -1.233261 | 0.3993845 | -4.114879 | 3.64108 | 2.881617 | 3.675716 | 0.433 | 1.47E-10 |
| LC + ERCP (two-stage) VS LC + LCBDE + TTD | -0.3173955 | 1.118438 | 0.7073625 | 1.49232 | -1.024758 | 1.923005 | 0.594 | 6.71E-09 |

Abbreviations: LC, laparoscopic cholecystectomy; LCBDE, laparoscopic common bile duct exploration; LTCBDE, laparoscopic transcystic bile duct exploration; ERCP, endoscopic retrograde cholangiopancreatography; PS, primary suture; TTD, T-tube drainage.

**5.15 Symptomatic biliary stricture**

**Table S5.15a:** Side-splitting of symptomatic biliary stricture in RCTs. Inconsistency test between direct and indirect treatment comparisons in mixed treatment comparison.

| Comparison | Direct | | Indirect | | Difference | | | tau |
| --- | --- | --- | --- | --- | --- | --- | --- | --- |
|  | Coef. | Std. Err. | Coef. | Std. Err. | Coef. | Std. Err. | P>\|z\| |  |
| LC + LCBDE + PS VS LC + LCBDE + TTD | 0.0106477 | 1.422316 | 1.609334 | 70.72683 | -1.598686 | 70.74113 | 0.982 | 9.91E-07 |
| LC + ERCP (two-stage) VS LC + LCBDE (mixed) | -1.74E-08 | 2.005908 | 3.196238 | 199.996 | -3.196238 | 199.9859 | 0.987 | 1.80E-06 |
| LC + ERCP (two-stage) VS LC + LCBDE + TTD | 1.60936 | 1.5559 | -0.3086828 | 89.46078 | 1.918043 | 89.47971 | 0.983 | 4.61E-06 |

Abbreviations: LC, laparoscopic cholecystectomy; LCBDE, laparoscopic common bile duct exploration; LTCBDE, laparoscopic transcystic bile duct exploration; ERCP, endoscopic retrograde cholangiopancreatography; PS, primary suture; TTD, T-tube drainage.

**Table S5.15b:** Side-splitting of symptomatic biliary stricture in Cohort studies. Inconsistency test between direct and indirect treatment comparisons in mixed treatment comparison.

| Comparison | Direct | | Indirect | | Difference | | | tau |
| --- | --- | --- | --- | --- | --- | --- | --- | --- |
|  | Coef. | Std. Err. | Coef. | Std. Err. | Coef. | Std. Err. | P>\|z\| |  |
| LC + LCBDE + PS VS LC + ERCP (two-stage) | -0.2168877 | 1.634509 | -1.730753 | 1.940673 | 1.513865 | 2.537288 | 0.551 | 5.04E-06 |
| LC + LCBDE + PS VS LC + LCBDE + TTD | 0.5994529 | 1.159775 | 2.115275 | 2.257182 | -1.515822 | 2.537706 | 0.55 | 2.75E-07 |
| LC + ERCP (one-stage) VS LC + ERCP (two-stage) | -0.270875 | 2.019491 | -1.41955 | 200.0166 | 1.148675 | 200.0471 | 0.995 | 5.36E-10 |
| LC + ERCP (two-stage) VS LC + LCBDE (mixed) | 0.293511 | 1.635717 | 1.984021 | 200.0591 | -1.69051 | 200.0524 | 0.993 | 8.94E-10 |
| LC + ERCP (two-stage) VS LC + LCBDE + TTD | 2.331943 | 1.55732 | 0.8162326 | 2.003803 | 1.51571 | 2.53784 | 0.55 | 7.64E-07 |

Abbreviations: LC, laparoscopic cholecystectomy; LCBDE, laparoscopic common bile duct exploration; LTCBDE, laparoscopic transcystic bile duct exploration; ERCP, endoscopic retrograde cholangiopancreatography; PS, primary suture; TTD, T-tube drainage.

**5.16 Stone recurrence**

**Table S5.16a:** Side-splitting of stone recurrence in RCTs. Inconsistency test between direct and indirect treatment comparisons in mixed treatment comparison.

| Comparison | Direct | | Indirect | | Difference | | | tau |
| --- | --- | --- | --- | --- | --- | --- | --- | --- |
|  | Coef. | Std. Err. | Coef. | Std. Err. | Coef. | Std. Err. | P>\|z\| |  |
| LC + LCBDE + PS VS LC + ERCP (two-stage) | -1.186581 | 1.646423 | 2.136076 | 1.322825 | -3.322658 | 2.112007 | 0.116 | 8.00E-10 |
| LC + LCBDE + PS VS LC + LCBDE + TTD | 0.5339928 | 1.057418 | -2.788046 | 1.82765 | 3.322039 | 2.111501 | 0.116 | 5.75E-10 |
| LC + ERCP (one-stage) VS LC + ERCP (two-stage) | -0.0201645 | 2.221156 | 1.769281 | 2.219094 | -1.789446 | 3.139888 | 0.569 | 0.9559475 |
| LC + ERCP (one-stage) VS LC + LCBDE (mixed) | 2.187574 | 1.776443 | 0.3978177 | 2.589314 | 1.789756 | 3.140167 | 0.569 | 0.9562538 |
| LC + ERCP (two-stage) VS LC + LTCBDE | 0.0354019 | 2.108922 | -1.322633 | 200.0153 | 1.358035 | 200.0062 | 0.995 | 0.6419849 |
| LC + ERCP (two-stage) VS LC + LCBDE (mixed) | 0.4177976 | 1.331228 | 2.206552 | 2.843175 | -1.788755 | 3.139341 | 0.569 | 0.9561966 |
| LC + ERCP (two-stage) VS LC + LCBDE + TTD | -1.603592 | 0.7957734 | 1.71879 | 1.955976 | -3.322382 | 2.111669 | 0.116 | 1.95E-09 |

Abbreviations: LC, laparoscopic cholecystectomy; LCBDE, laparoscopic common bile duct exploration; LTCBDE, laparoscopic transcystic bile duct exploration; ERCP, endoscopic retrograde cholangiopancreatography; PS, primary suture; TTD, T-tube drainage.

**Table S5.16b:** Side-splitting of stone recurrence in Cohort studies. Inconsistency test between direct and indirect treatment comparisons in mixed treatment comparison.

| Comparison | Direct | | Indirect | | Difference | | | tau |
| --- | --- | --- | --- | --- | --- | --- | --- | --- |
|  | Coef. | Std. Err. | Coef. | Std. Err. | Coef. | Std. Err. | P>\|z\| |  |
| LC + LCBDE + PS VS LC + ERCP (one-stage) | -1.437067 | 0.8033167 | 1.020012 | 0.5038455 | -2.457079 | 0.9482499 | 0.01 | 4.41E-08 |
| LC + LCBDE + PS VS LC + ERCP (two-stage) | 0.8573792 | 0.3729321 | 0.1262124 | 0.7053977 | 0.7311668 | 0.7979907 | 0.36 | 0.3495703 |
| LC + LCBDE + PS VS LC + LTCBDE | -1.376138 | 0.8194343 | -0.3884295 | 1.484579 | -0.9877086 | 1.493443 | 0.508 | 0.4222993 |
| LC + LCBDE + PS VS LC + LCBDE + TTD | 0.064617 | 0.4529312 | -0.7556034 | 0.8378045 | 0.8202204 | 0.9630477 | 0.394 | 0.3758172 |
| LC + ERCP (one-stage) VS LC + ERCP (two-stage) | -0.2707683 | 2.060512 | 0.6397234 | 0.6025421 | -0.9104916 | 2.146816 | 0.671 | 0.4096128 |
| LC + ERCP (one-stage) VS LC + LCBDE (mixed) | -0.6687501 | 0.2869921 | 1.381355 | 0.8435228 | -2.050105 | 0.8910075 | 0.021 | 0.0165895 |
| LC + ERCP (two-stage) VS LC + LTCBDE | -1.734038 | 1.310197 | -1.999615 | 0.9162234 | 0.2655765 | 1.412754 | 0.851 | 0.4226047 |
| LC + ERCP (two-stage) VS LC + LCBDE (mixed) | -0.5588496 | 0.2915269 | -2.608898 | 0.8421302 | 2.050049 | 0.8909787 | 0.021 | 0.0168547 |
| LC + ERCP (two-stage) VS LC + LCBDE + TTD | -0.9818208 | 0.5590176 | -0.6189009 | 0.6576575 | -0.3629199 | 0.8525305 | 0.67 | 0.4120716 |
| LC + LTCBDE VS LC + LCBDE + TTD | 0.5678611 | 1.199208 | 1.705856 | 1.303563 | -1.137995 | 1.89272 | 0.548 | 0.3897201 |

Abbreviations: LC, laparoscopic cholecystectomy; LCBDE, laparoscopic common bile duct exploration; LTCBDE, laparoscopic transcystic bile duct exploration; ERCP, endoscopic retrograde cholangiopancreatography; PS, primary suture; TTD, T-tube drainage.

# Appendix 6: Network maps and forest plots of outcomes

**6.1 Single-session stone clearance rate**

Network map of single-session stone clearance rate and forest plot of network effect estimates compared with the reference treatment. The size of the nodes is proportional to the number of participants included in the trials, and the thickness of the lines between interventions reflects the number of studies for each comparison.

**Figure S6.1:** Network map and Forest plot of Cohort studies

Abbreviations: LC, laparoscopic cholecystectomy; LCBDE, laparoscopic common bile duct exploration; LTCBDE, laparoscopic transcystic bile duct exploration; ERCP, endoscopic retrograde cholangiopancreatography; PS,primary suture; TTD,T-tube drainage .

**6.2 Severe postoperative complications**

Network map of operative time and forest plot of network effect estimates compared with the reference treatment. The size of the nodes is proportional to the number of participants included in the trials, and the thickness of the lines between interventions reflects the number of studies for each comparison.

**Figure S6.2:** Network map and Forest plot of Cohort studies

Abbreviations: LC, laparoscopic cholecystectomy; LCBDE, laparoscopic common bile duct exploration; LTCBDE, laparoscopic transcystic bile duct exploration; ERCP, endoscopic retrograde cholangiopancreatography; PS,primary suture; TTD,T-tube drainage .

**6.3 Operative time**

Network map of operative time and forest plot of network effect estimates compared with the reference treatment. The size of the nodes is proportional to the number of participants included in the trials, and the thickness of the lines between interventions reflects the number of studies for each comparison.

**Figure S6.3:** Network map and Forest plot of Cohort studies

Abbreviations: LC, laparoscopic cholecystectomy; LCBDE, laparoscopic common bile duct exploration; LTCBDE, laparoscopic transcystic bile duct exploration; ERCP, endoscopic retrograde cholangiopancreatography; PS,primary suture; TTD,T-tube drainage .

**6.4 Total length of hospital stay**

Network map of conversion to total length of hospital stay and forest plot of network effect estimates compared with the reference treatment. The size of the nodes is proportional to the number of participants included in the trials, and the thickness of the lines between interventions reflects the number of studies for each comparison.

**Figure S6.5:** Network map and Forest plot of Cohort studies

Abbreviations: LC, laparoscopic cholecystectomy; LCBDE, laparoscopic common bile duct exploration;LTCBDE, laparoscopic transcystic bile duct exploration; ERCP, endoscopic retrograde cholangiopancreatography; PS,primary suture ;TTD,T-tube drainage .

**6.5: Intraoperative blood loss**

Network map of intraoperative blood loss and forest plot of network effect estimates compared with the reference treatment. The size of the nodes is proportional to the number of participants included in the trials, and the thickness of the lines between interventions reflects the number of studies for each comparison.

**Figure S6.4:** Network map and Forest plot of Cohort studies

Abbreviations: LC, laparoscopic cholecystectomy; LCBDE, laparoscopic common bile duct exploration;LTCBDE, laparoscopic transcystic bile duct exploration; ERCP, endoscopic retrograde cholangiopancreatography; PS,primary suture ;TTD,T-tube drainage .

**6.6 Conversion to open surgery**

Network map of conversion to open surgery and forest plot of network effect estimates compared with the reference treatment. The size of the nodes is proportional to the number of participants included in the trials, and the thickness of the lines between interventions reflects the number of studies for each comparison.

**Figure S6.5a:** Network map and Forest plot of RCTs

Abbreviations: LC, laparoscopic cholecystectomy; LCBDE, laparoscopic common bile duct exploration;LTCBDE, laparoscopic transcystic bile duct exploration; ERCP, endoscopic retrograde cholangiopancreatography; PS,primary suture ;TTD,T-tube drainage .

**Figure S6.6b:** Network map and Forest plot of Cohort studies

Abbreviations: LC, laparoscopic cholecystectomy; LCBDE, laparoscopic common bile duct exploration;LTCBDE, laparoscopic transcystic bile duct exploration; ERCP, endoscopic retrograde cholangiopancreatography; PS,primary suture ;TTD,T-tube drainage .

**6.7 Reintervention**

Network map of reintervention and forest plot of network effect estimates compared with the reference treatment. The size of the nodes is proportional to the number of participants included in the trials, and the thickness of the lines between interventions reflects the number of studies for each comparison.

**Figure S6.6a:** Network map and Forest plot of RCTs

Abbreviations: LC, laparoscopic cholecystectomy; LCBDE, laparoscopic common bile duct exploration;LTCBDE, laparoscopic transcystic bile duct exploration; ERCP, endoscopic retrograde cholangiopancreatography; PS,primary suture ;TTD,T-tube drainage .

**Figure S6.7b:** Network map and Forest plot of Cohort studies

Abbreviations: LC, laparoscopic cholecystectomy; LCBDE, laparoscopic common bile duct exploration;LTCBDE, laparoscopic transcystic bile duct exploration; ERCP, endoscopic retrograde cholangiopancreatography; PS,primary suture ;TTD,T-tube drainage .

**6.8: Postoperative bile leakage**

Network map of Postoperative bile leakage and forest plot of network effect estimates compared with the reference treatment. The size of the nodes is proportional to the number of participants included in the trials, and the thickness of the lines between interventions reflects the number of studies for each comparison.

**Figure S6.8a:** Network map and Forest plot of RCTs

Abbreviations: LC, laparoscopic cholecystectomy; LCBDE, laparoscopic common bile duct exploration;LTCBDE, laparoscopic transcystic bile duct exploration; ERCP, endoscopic retrograde cholangiopancreatography; PS,primary suture ;TTD,T-tube drainage .

**Figure S6.8b:** Network map and Forest plot of Cohort studies

Abbreviations: LC, laparoscopic cholecystectomy; LCBDE, laparoscopic common bile duct exploration;LTCBDE, laparoscopic transcystic bile duct exploration; ERCP, endoscopic retrograde cholangiopancreatography; PS,primary suture ;TTD,T-tube drainage .

**6.9 Postoperative pancreatitis**

Network map of Postoperative pancreatitis and forest plot of network effect estimates compared with the reference treatment. The size of the nodes is proportional to the number of participants included in the trials, and the thickness of the lines between interventions reflects the number of studies for each comparison.

**Figure S6.9a:** Network map and Forest plot of RCTs

Abbreviations: LC, laparoscopic cholecystectomy; LCBDE, laparoscopic common bile duct exploration;LTCBDE, laparoscopic transcystic bile duct exploration; ERCP, endoscopic retrograde cholangiopancreatography; PS,primary suture ;TTD,T-tube drainage .

**Figure S6.9b:** Network map and Forest plot of Cohort Studies

Abbreviations: LC, laparoscopic cholecystectomy; LCBDE, laparoscopic common bile duct exploration;LTCBDE, laparoscopic transcystic bile duct exploration; ERCP, endoscopic retrograde cholangiopancreatography; PS,primary suture ;TTD,T-tube drainage .

**6.10** Postoperative biliary bleeding

Network map of postoperative biliary bleeding and forest plot of network effect estimates compared with the reference treatment. The size of the nodes is proportional to the number of participants included in the trials, and the thickness of the lines between interventions reflects the number of studies for each comparison.

**Figure S6.10a:** Network map and Forest plot of RCTs

Abbreviations:LC, laparoscopic cholecystectomy; LCBDE, laparoscopic common bile duct exploration;LTCBDE, laparoscopic transcystic bile duct exploration; ERCP, endoscopic retrograde cholangiopancreatography; PS,primary suture ;TTD,T-tube drainage .

**Figure S6.10b:** Network map and Forest plot of Cohort Studies

Abbreviations:LC, laparoscopic cholecystectomy; LCBDE, laparoscopic common bile duct exploration;LTCBDE, laparoscopic transcystic bile duct exploration; ERCP, endoscopic retrograde cholangiopancreatography; PS,primary suture ;TTD,T-tube drainage .

**6.11 Postoperative surgical site infection**

Network map of postoperative surgical site infection and forest plot of network effect estimates compared with the reference treatment. The size of the nodes is proportional to the number of participants included in the trials, and the thickness of the lines between interventions reflects the number of studies for each comparison.

**Figure S6.11a:** Network map and Forest plot of RCTs

Abbreviations: LC, laparoscopic cholecystectomy; LCBDE, laparoscopic common bile duct exploration;LTCBDE, laparoscopic transcystic bile duct exploration; ERCP, endoscopic retrograde cholangiopancreatography; PS,primary suture.

**Figure S6.11b:** Network map and Forest plot of Cohort Studies

Abbreviations: LC, laparoscopic cholecystectomy; LCBDE, laparoscopic common bile duct exploration;LTCBDE, laparoscopic transcystic bile duct exploration; ERCP, endoscopic retrograde cholangiopancreatography; PS,primary suture.

**6.12** Postoperative intra-abdominal infection

Network map of postoperative intra-abdominal infection and forest plot of network effect estimates compared with the reference treatment. The size of the nodes is proportional to the number of participants included in the trials, and the thickness of the lines between interventions reflects the number of studies for each comparison.

**Figure S6.12a:** Network map and Forest plot of RCTs

Abbreviations: LC, laparoscopic cholecystectomy; LCBDE, laparoscopic common bile duct exploration;LTCBDE, laparoscopic transcystic bile duct exploration; ERCP, endoscopic retrograde cholangiopancreatography; PS,primary suture ;TTD,T-tube drainage .

**Figure S6.12b:** Network map and Forest plot of Cohort Studies

Abbreviations: LC, laparoscopic cholecystectomy; LCBDE, laparoscopic common bile duct exploration;LTCBDE, laparoscopic transcystic bile duct exploration; ERCP, endoscopic retrograde cholangiopancreatography; PS,primary suture ;TTD,T-tube drainage.

**6.13** Postoperative cholangitis

Network map of postoperative cholangitis and forest plot of network effect estimates compared with the reference treatment. The size of the nodes is proportional to the number of participants included in the trials, and the thickness of the lines between interventions reflects the number of studies for each comparison.

**Figure S6.13a:** Network map and Forest plot of RCTs

Abbreviations: LC, laparoscopic cholecystectomy; LCBDE, laparoscopic common bile duct exploration;LTCBDE, laparoscopic transcystic bile duct exploration; ERCP, endoscopic retrograde cholangiopancreatography; PS,primary suture ;TTD,T-tube drainage.

**Figure S6.13b:** Network map and Forest plot of Cohort Studies

Abbreviations: LC, laparoscopic cholecystectomy; LCBDE, laparoscopic common bile duct exploration;LTCBDE, laparoscopic transcystic bile duct exploration; ERCP, endoscopic retrograde cholangiopancreatography; PS,primary suture ;TTD,T-tube drainage.

**6.14 Symptomatic biliary stricture**

Network map of symptomatic biliary stricture and forest plot of network effect estimates compared with the reference treatment. The size of the nodes is proportional to the number of participants included in the trials, and the thickness of the lines between interventions reflects the number of studies for each comparison.

**Figure S6.14a:** Network map and Forest plot of RCTs

Abbreviations: LC, laparoscopic cholecystectomy; LCBDE, laparoscopic common bile duct exploration;LTCBDE, laparoscopic transcystic bile duct exploration; ERCP, endoscopic retrograde cholangiopancreatography; PS,primary suture ;TTD,T-tube drainage.

**Figure S6.14b:** Network map and Forest plot of Cohort Studies

Abbreviations: LC, laparoscopic cholecystectomy; LCBDE, laparoscopic common bile duct exploration;LTCBDE, laparoscopic transcystic bile duct exploration; ERCP, endoscopic retrograde cholangiopancreatography; PS,primary suture ;TTD,T-tube drainage.

**6.15 Stone recurrence**

Network map of stone recurrence and forest plot of network effect estimates compared with the reference treatment. The size of the nodes is proportional to the number of participants included in the trials, and the thickness of the lines between interventions reflects the number of studies for each comparison.

**Figure S6.15a:** Network map and Forest plot of RCTs

Abbreviations: LC, laparoscopic cholecystectomy; LCBDE, laparoscopic common bile duct exploration;LTCBDE, laparoscopic transcystic bile duct exploration; ERCP, endoscopic retrograde cholangiopancreatography; PS,primary suture ;TTD,T-tube drainage.

**Figure S6.15b:** Network map and Forest plot of Cohort Studies

# Appendix 7: SUCRA and cumulative probability plots

**7.1 Single-session stone clearance rate**

**Figure S7.1a:** Cumulative ranking curve plots of different minimally invasive strategies for single-session stone clearance rate in RCTs. A higher surface under the cumulative ranking curve indicates a higher probability of better single-session stone clearance rate.

Abbreviations: LC, laparoscopic cholecystectomy; LCBDE, laparoscopic common bile duct exploration; LTCBDE, laparoscopic transcystic bile duct exploration; ERCP, endoscopic retrograde cholangiopancreatography; PS, primary suture; TTD,T-tube drainage.

**Table S7.1a:** SUCRA of the effects of different interventions for single-session stone clearance rate in RCTs.

| **Intervention** | **SUCRA (%)** | **PrBest (%)** | **Mean rank** |
| --- | --- | --- | --- |
| LC + LCBDE + PS | 82.4 | 41.5 | 1.9 |
| LC + LCBDE + TTD | 60.1 | 8.6 | 3 |
| LC + ERCP (one-stage) | 59.6 | 17.6 | 3 |
| LC + LTCBDE | 57.1 | 31.6 | 3.1 |
| LC + LCBDE (mixed) | 30.9 | 0.7 | 4.5 |
| LC + ERCP (two-stage) | 9.9 | 0 | 5.5 |

Abbreviations: SUCRA, surface under the cumulative ranking curve; PrBest, probability of best; LC, laparoscopic cholecystectomy; LCBDE, laparoscopic common bile duct exploration; LTCBDE, laparoscopic transcystic bile duct exploration; ERCP, endoscopic retrograde cholangiopancreatography; PS, primary suture; TTD,T-tube drainage.

**Figure S7.1b:** Cumulative ranking curve plots of different minimally invasive strategies for single-session stone clearance rate in Cohort studies. A higher surface under the cumulative ranking curve indicates a higher probability of better single-session stone clearance rate.

Abbreviations: LC, laparoscopic cholecystectomy; LCBDE, laparoscopic common bile duct exploration; LTCBDE, laparoscopic transcystic bile duct exploration; ERCP, endoscopic retrograde cholangiopancreatography; PS, primary suture; TTD,T-tube drainage.

**Table S7.1b:** SUCRA of the effects of different interventions for single-session stone clearance rate in Cohort studies.

| **Intervention** | **SUCRA (%)** | **PrBest (%)** | **Mean rank** |
| --- | --- | --- | --- |
| **LC + ERCP (one-stage)** | **78.4** | **51.0** | **2.1** |
| **LC + LCBDE (mixed)** | **62.4** | **16.5** | **2.9** |
| **LC + LCBDE + PS** | **59.9** | **11.7** | **3.0** |
| **LC + LTCBDE** | **48.8** | **11.8** | **3.6** |
| **LC + LCBDE + TTD** | **46.7** | **9.1** | **3.7** |
| **LC + ERCP (two-stage)** | **3.7** | **0.0** | **5.8** |

Abbreviations: SUCRA, surface under the cumulative ranking curve; PrBest, probability of best; LC, laparoscopic cholecystectomy; LCBDE, laparoscopic common bile duct exploration; LTCBDE, laparoscopic transcystic bile duct exploration; ERCP, endoscopic retrograde cholangiopancreatography; PS, primary suture; TTD,T-tube drainage.

**7.2 Severe postoperative complications**

**Figure S7.2a:** Cumulative ranking curve plots of different minimally invasive strategies for severe postoperative complications in RCTs. A higher surface under the cumulative ranking curve indicates a higher probability of fewer severe postoperative complications.

Abbreviations: LC, laparoscopic cholecystectomy; LCBDE, laparoscopic common bile duct exploration; LTCBDE, laparoscopic transcystic bile duct exploration; ERCP, endoscopic retrograde cholangiopancreatography; PS, primary suture;TTD,T-tube drainage.

**Table S7.2a:** SUCRA of the effects of different minimally invasive strategies for severe postoperative complications in RCTs.

| **Intervention** | **SUCRA (%)** | **PrBest (%)** | **Mean rank** |
| --- | --- | --- | --- |
| **LC + ERCP (one-stage)** | **77.3** | **33.4** | **2.1** |
| **LC + LCBDE (mixed)** | **73.8** | **30.3** | **2.3** |
| **LC + LCBDE + PS** | **60.5** | **10.0** | **3.0** |
| **LC + LTCBDE** | **41.8** | **25.5** | **3.9** |
| **LC + ERCP (two-stage)** | **25.8** | **0.1** | **4.7** |
| **LC + LCBDE + TTD** | **20.9** | **0.6** | **5.0** |

Abbreviations: SUCRA, surface under the cumulative ranking curve; PrBest, probability of best; LC, laparoscopic cholecystectomy; LCBDE, laparoscopic common bile duct exploration; LTCBDE, laparoscopic transcystic bile duct exploration; ERCP, endoscopic retrograde cholangiopancreatography; PS, primary suture; TTD,T-tube drainage.rograde cholangiopancreatography; PS, primary suture; TTD,T-tube drainage.

**Figure S7.2b:** Cumulative ranking curve plots of different minimally invasive strategies for severe postoperative complications in Cohort studies. A higher surface under the cumulative ranking curve indicates a higher probability of fewer severe postoperative complications.

Abbreviations: LC, laparoscopic cholecystectomy; LCBDE, laparoscopic common bile duct exploration; LTCBDE, laparoscopic transcystic bile duct exploration; ERCP, endoscopic retrograde cholangiopancreatography; PS, primary suture;TTD,T-tube drainage.

**Table S7.2b:** SUCRA of the effects of different minimally invasive strategies for severe postoperative complications in Cohort studies.

| **Intervention** | **SUCRA (%)** | **PrBest (%)** | **Mean rank** |
| --- | --- | --- | --- |
| **LC + LCBDE (mixed)** | **73.7** | **26.6** | **2.3** |
| **LC + LCBDE + TTD** | **62.6** | **26.1** | **2.9** |
| **LC + LTCBDE** | **52.5** | **24.0** | **3.4** |
| **LC + ERCP (one-stage)** | **45.3** | **17.8** | **3.7** |
| **LC + ERCP (two-stage)** | **34.6** | **0.2** | **4.3** |
| **LC + LCBDE + PS** | **31.2** | **5.2** | **4.4** |

Abbreviations: SUCRA, surface under the cumulative ranking curve; PrBest, probability of best; LC, laparoscopic cholecystectomy; LCBDE, laparoscopic common bile duct exploration; LTCBDE, laparoscopic transcystic bile duct exploration; ERCP, endoscopic retrograde cholangiopancreatography; PS, primary suture; TTD,T-tube drainage.rograde cholangiopancreatography; PS, primary suture; TTD,T-tube drainage.

**7.3 Operative time**

**Figure S7.3a:** Cumulative ranking curve plots of different minimally invasive strategies for operative time in RCTs. A higher surface under the cumulative ranking curve indicates a higher probability of shorter operative time.

Abbreviations: LC, laparoscopic cholecystectomy; LCBDE, laparoscopic common bile duct exploration; LTCBDE, laparoscopic transcystic bile duct exploration; ERCP, endoscopic retrograde cholangiopancreatography; PS, primary suture;TTD,T-tube drainage.

**Table S7.3a:** SUCRA of the effects of different minimally invasive strategies for operative time in RCTs.

| **Intervention** | **SUCRA (%)** | **PrBest (%)** | **Mean rank** |
| --- | --- | --- | --- |
| LC + LTCBDE | 96.4 | 83.3 | 1.2 |
| LC + ERCP (two-stage) | 82.8 | 16.7 | 1.9 |
| LC + LCBDE + PS | 49.5 | 0 | 3.5 |
| LC + ERCP (one-stage) | 39.2 | 0 | 4 |
| LC + LCBDE (mixed) | 26.2 | 0 | 4.7 |
| LC + LCBDE + TTD | 6 | 0 | 5.7 |

Abbreviations: SUCRA, surface under the cumulative ranking curve; PrBest, probability of best; LC, laparoscopic cholecystectomy; LCBDE, laparoscopic common bile duct exploration; LTCBDE, laparoscopic transcystic bile duct exploration; ERCP, endoscopic retrograde cholangiopancreatography; PS, primary suture; TTD,T-tube drainage.

**Figure S7.3b:** Cumulative ranking curve plots of different minimally invasive strategies for operative time in Cohort studies. A higher surface under the cumulative ranking curve indicates a higher probability of shorter operative time.

Abbreviations: LC, laparoscopic cholecystectomy; LCBDE, laparoscopic common bile duct exploration; LTCBDE, laparoscopic transcystic bile duct exploration; ERCP, endoscopic retrograde cholangiopancreatography; PS, primary suture;TTD,T-tube drainage.

**Table S7.3b:** SUCRA of the effects of different minimally invasive strategies for operative time in Cohort studies.

| **Intervention** | **SUCRA (%)** | **PrBest (%)** | **Mean rank** |
| --- | --- | --- | --- |
| **LC + LTCBDE** | **94.0** | **81.5** | **1.3** |
| **LC + ERCP (two-stage)** | **72.2** | **12.5** | **2.4** |
| **LC + LCBDE + PS** | **51.8** | **2.9** | **3.4** |
| **LC + LCBDE + TTD** | **42.7** | **2.7** | **3.9** |
| **LC + LCBDE (mixed)** | **19.9** | **0.2** | **5.0** |
| **LC + ERCP (one-stage)** | **19.5** | **0.3** | **5.0** |

Abbreviations: SUCRA, surface under the cumulative ranking curve; PrBest, probability of best; LC, laparoscopic cholecystectomy; LCBDE, laparoscopic common bile duct exploration; LTCBDE, laparoscopic transcystic bile duct exploration; ERCP, endoscopic retrograde cholangiopancreatography; PS, primary suture; TTD,T-tube drainage.

**7.4 Total length of hospital stay**

**Figure S7.4a:** Cumulative ranking curve plots of different minimally invasive strategies for total length of hospital stay in RCTs. A higher surface under the cumulative ranking curve indicates a higher probability of shorter total length of hospital stay.

Abbreviations: LC, laparoscopic cholecystectomy; LCBDE, laparoscopic common bile duct exploration; LTCBDE, laparoscopic transcystic bile duct exploration; ERCP, endoscopic retrograde cholangiopancreatography; PS, primary suture;TTD,T-tube drainage.

**Table S7.4a:** SUCRA of the effects of different minimally invasive strategies for total length of hospital stay in RCTs.

| **Intervention** | **SUCRA (%)** | **PrBest (%)** | **Mean rank** |
| --- | --- | --- | --- |
| **LC + LTCBDE** | **86.9** | **58.1** | **1.7** |
| **LC + LCBDE (mixed)** | **77.4** | **25.7** | **2.1** |
| **LC + ERCP (one-stage)** | **73.8** | **16.2** | **2.3** |
| **LC + LCBDE + PS** | **33.8** | **0.1** | **4.3** |
| **LC + ERCP (two-stage)** | **28.0** | **0.0** | **4.6** |
| **LC + LCBDE + TTD** | **0.1** | **0.0** | **6.0** |

Abbreviations: SUCRA, surface under the cumulative ranking curve; PrBest, probability of best; LC, laparoscopic cholecystectomy; LCBDE, laparoscopic common bile duct exploration; LTCBDE, laparoscopic transcystic bile duct exploration; ERCP, endoscopic retrograde cholangiopancreatography; PS, primary suture; TTD,T-tube drainage.

**Figure S7.4b:** Cumulative ranking curve plots of different minimally invasive strategies for total length of hospital stay in Cohort studies. A higher surface under the cumulative ranking curve indicates a higher probability of shorter total length of hospital stay.

Abbreviations: LC, laparoscopic cholecystectomy; LCBDE, laparoscopic common bile duct exploration; LTCBDE, laparoscopic transcystic bile duct exploration; ERCP, endoscopic retrograde cholangiopancreatography; PS, primary suture;TTD,T-tube drainage.

**Table S7.4b:** SUCRA of the effects of different minimally invasive strategies for total length of hospital stay in Cohort studies.

| **Intervention** | **SUCRA (%)** | **PrBest (%)** | **Mean rank** |
| --- | --- | --- | --- |
| **LC + LTCBDE** | **91.7** | **71.5** | **1.4** |
| **LC + LCBDE (mixed)** | **74.6** | **21.4** | **2.3** |
| **LC + LCBDE + PS** | **62.6** | **5.7** | **2.9** |
| **LC + ERCP (one-stage)** | **49.5** | **1.4** | **3.5** |
| **LC + ERCP (two-stage)** | **15.6** | **0.0** | **5.2** |
| **LC + LCBDE + TTD** | **5.9** | **0.0** | **5.7** |

Abbreviations: SUCRA, surface under the cumulative ranking curve; PrBest, probability of best; LC, laparoscopic cholecystectomy; LCBDE, laparoscopic common bile duct exploration; LTCBDE, laparoscopic transcystic bile duct exploration; ERCP, endoscopic retrograde cholangiopancreatography; PS, primary suture; TTD,T-tube drainage.

**7.5** Intraoperative blood loss

**Figure S7.5:** Cumulative ranking curve plots of different minimally invasive strategies for intraoperative blood loss in Cohort studies.A higher surface under the cumulative ranking curve indicates a higher probability of lower intraoperative blood loss.

Abbreviations: LC, laparoscopic cholecystectomy; LCBDE, laparoscopic common bile duct exploration; LTCBDE, laparoscopic transcystic bile duct exploration; ERCP, endoscopic retrograde cholangiopancreatography; PS, primary suture;TTD,T-tube drainage.

**Table S7.5:** SUCRA of the effects of different minimally invasive strategies for intraoperative blood loss in Cohort studies.

| **Intervention** | **SUCRA (%)** | **PrBest (%)** | **Mean rank** |
| --- | --- | --- | --- |
| **LC + LTCBDE** | **94.8** | **77.1** | **1.3** |
| **LC + LCBDE (mixed)** | **67.0** | **10.5** | **2.7** |
| **LC + ERCP (one-stage)** | **55.2** | **11.4** | **3.2** |
| **LC + LCBDE + TTD** | **42.4** | **0.8** | **3.9** |
| **LC + ERCP (two-stage)** | **26.8** | **0.0** | **4.7** |
| **LC + LCBDE + PS** | **13.9** | **0.0** | **5.3** |

Abbreviations: SUCRA, surface under the cumulative ranking curve; PrBest, probability of best; LC, laparoscopic cholecystectomy; LCBDE, laparoscopic common bile duct exploration; LTCBDE, laparoscopic transcystic bile duct exploration; ERCP, endoscopic retrograde cholangiopancreatography; PS, primary suture; TTD,T-tube drainage.

**7.6 Conversion to open surgery**

**Figure S7.6a:** Cumulative ranking curve plots of different minimally invasive strategies for conversion to open surgery in RCTs. A higher surface under the cumulative ranking curve indicates a greater likelihood of lower conversion to open surgery.

Abbreviations: LC, laparoscopic cholecystectomy; LCBDE, laparoscopic common bile duct exploration; LTCBDE, laparoscopic transcystic bile duct exploration; ERCP, endoscopic retrograde cholangiopancreatography; PS, primary suture;TTD,T-tube drainage.

**Table S7.6a:** SUCRA of the effects of different minimally invasive strategies for conversion to open surgery in RCTs.

| **Intervention** | **SUCRA (%)** | **PrBest (%)** | **Mean rank** |
| --- | --- | --- | --- |
| **LC + LCBDE + PS** | **62.8** | **18.6** | **2.9** |
| **LC + LCBDE + TTD** | **54.9** | **19.3** | **3.3** |
| **LC + LTCBDE** | **57.3** | **40.1** | **3.1** |
| **LC + ERCP (one-stage)** | **45.0** | **3.7** | **3.7** |
| **LC + ERCP (two-stage)** | **41.8** | **9.8** | **3.9** |
| **LC + LCBDE (mixed)** | **38.3** | **8.6** | **4.1** |

Abbreviations: SUCRA, surface under the cumulative ranking curve; PrBest, probability of best; LC, laparoscopic cholecystectomy; LCBDE, laparoscopic common bile duct exploration; LTCBDE, laparoscopic transcystic bile duct exploration; ERCP, endoscopic retrograde cholangiopancreatography; PS, primary suture; TTD,T-tube drainage.

**Figure S7.6b:** Cumulative ranking curve plots of different minimally invasive strategies for conversion to open surgery in Cohort studies. A higher surface under the cumulative ranking curve indicates a greater likelihood of lower conversion to open surgery.

Abbreviations: LC, laparoscopic cholecystectomy; LCBDE, laparoscopic common bile duct exploration; LTCBDE, laparoscopic transcystic bile duct exploration; ERCP, endoscopic retrograde cholangiopancreatography; PS, primary suture;TTD,T-tube drainage.

**Table S7.6b:** SUCRA of the effects of different minimally invasive strategies for conversion to open surgery in Cohort studies.

| **Intervention** | **SUCRA (%)** | **PrBest (%)** | **Mean rank** |
| --- | --- | --- | --- |
| **LC + LCBDE (mixed)** | **77.5** | **37.9** | **2.1** |
| **LC + LCBDE + PS** | **63.0** | **22.4** | **2.9** |
| **LC + LTCBDE** | **50.9** | **23.4** | **3.5** |
| **LC + ERCP (one-stage)** | **45.9** | **8.7** | **3.7** |
| **LC + LCBDE + TTD** | **33.7** | **7.2** | **4.3** |
| **LC + ERCP (two-stage)** | **29.0** | **0.4** | **4.5** |

Abbreviations: SUCRA, surface under the cumulative ranking curve; PrBest, probability of best; LC, laparoscopic cholecystectomy; LCBDE, laparoscopic common bile duct exploration; LTCBDE, laparoscopic transcystic bile duct exploration; ERCP, endoscopic retrograde cholangiopancreatography; PS, primary suture; TTD,T-tube drainage.

**7.7 Reintervention**

**Figure S7.7a:** Cumulative ranking curve plots of different minimally invasive strategies for reintervention in RCTs.

A higher surface under the cumulative ranking curve indicates a higher probability of lower reintervention.

Abbreviations: LC, laparoscopic cholecystectomy; LCBDE, laparoscopic common bile duct exploration; LTCBDE, laparoscopic transcystic bile duct exploration; ERCP, endoscopic retrograde cholangiopancreatography; PS, primary suture;TTD,T-tube drainage.

**Table S7.7a:** SUCRA of the effects of different minimally invasive strategies for reintervention in RCTs.

| **Intervention** | **SUCRA (%)** | **PrBest (%)** | **Mean rank** |
| --- | --- | --- | --- |
| **LC + LCBDE (mixed)** | **74.8** | **37.4** | **2.3** |
| **LC + LCBDE + PS** | **71.2** | **22.0** | **2.4** |
| **LC + LCBDE + TTD** | **64.8** | **23.8** | **2.8** |
| **LC + ERCP (one-stage)** | **58.5** | **12.6** | **3.1** |
| **LC + ERCP (two-stage)** | **17.2** | **0.0** | **5.1** |
| **LC + LTCBDE** | **13.4** | **4.1** | **5.3** |

Abbreviations: SUCRA, surface under the cumulative ranking curve; PrBest, probability of best; LC, laparoscopic cholecystectomy; LCBDE, laparoscopic common bile duct exploration; LTCBDE, laparoscopic transcystic bile duct exploration; ERCP, endoscopic retrograde cholangiopancreatography; PS, primary suture; TTD,T-tube drainage.

**Figure S7.7b:** Cumulative ranking curve plots of different minimally invasive strategies for reintervention in Cohort studies.

A higher surface under the cumulative ranking curve indicates a higher probability of lower reintervention.

Abbreviations: LC, laparoscopic cholecystectomy; LCBDE, laparoscopic common bile duct exploration; LTCBDE, laparoscopic transcystic bile duct exploration; ERCP, endoscopic retrograde cholangiopancreatography; PS, primary suture;TTD,T-tube drainage.

**Table S7.7b:** SUCRA of the effects of different minimally invasive strategies for reintervention in Cohort studies.

| **Intervention** | **SUCRA (%)** | **PrBest (%)** | **Mean rank** |
| --- | --- | --- | --- |
| **LC + LCBDE (mixed)** | **84.1** | **52.3** | **1.8** |
| **LC + LTCBDE** | **73.0** | **27.0** | **2.4** |
| **LC + LCBDE + TTD** | **57.5** | **17.0** | **3.1** |
| **LC + LCBDE + PS** | **51.6** | **2.7** | **3.4** |
| **LC + ERCP (one-stage)** | **24.5** | **1.0** | **4.8** |
| **LC + ERCP (two-stage)** | **9.3** | **0.0** | **5.5** |

Abbreviations: SUCRA, surface under the cumulative ranking curve; PrBest, probability of best; LC, laparoscopic cholecystectomy; LCBDE, laparoscopic common bile duct exploration; LTCBDE, laparoscopic transcystic bile duct exploration; ERCP, endoscopic retrograde cholangiopancreatography; PS, primary suture; TTD,T-tube drainage.

**7.8 Postoperative bile leakage**

**Figure S7.8a:** Cumulative ranking curve plots of different minimally invasive strategies for postoperative bile leakage in RCTs. A higher surface under the cumulative ranking curve indicates a higher probability of lower postoperative bile leakage.

Abbreviations: LC, laparoscopic cholecystectomy; LCBDE, laparoscopic common bile duct exploration; LTCBDE, laparoscopic transcystic bile duct exploration; ERCP, endoscopic retrograde cholangiopancreatography; PS, primary suture;TTD,T-tube drainage.

**Table S7.8a:** SUCRA of the effects of different minimally invasive strategies for postoperative bile leakage in RCTs.

| **Intervention** | **SUCRA (%)** | **PrBest (%)** | **Mean rank** |
| --- | --- | --- | --- |
| **LC + ERCP (two-stage)** | **78.6** | **25.4** | **2.1** |
| **LC + ERCP (one-stage)** | **71.1** | **24.1** | **2.4** |
| **LC + LTCBDE** | **62.8** | **46.0** | **2.9** |
| **LC + LCBDE + TTD** | **41.4** | **3.3** | **3.9** |
| **LC + LCBDE + PS** | **37.1** | **1.2** | **4.1** |
| **LC + LCBDE (mixed)** | **9.0** | **0.0** | **5.6** |

Abbreviations: SUCRA, surface under the cumulative ranking curve; PrBest, probability of best; LC, laparoscopic cholecystectomy; LCBDE, laparoscopic common bile duct exploration; LTCBDE, laparoscopic transcystic bile duct exploration; ERCP, endoscopic retrograde cholangiopancreatography; PS, primary suture; TTD,T-tube drainage.

**Figure S7.8b:** Cumulative ranking curve plots of different minimally invasive strategies for postoperative bile leakage in Cohort studies. A higher surface under the cumulative ranking curve indicates a higher probability of lower postoperative bile leakage.

Abbreviations: LC, laparoscopic cholecystectomy; LCBDE, laparoscopic common bile duct exploration; LTCBDE, laparoscopic transcystic bile duct exploration; ERCP, endoscopic retrograde cholangiopancreatography; PS, primary suture;TTD,T-tube drainage.

**Table S7.8b:** SUCRA of the effects of different minimally invasive strategies for postoperative bile leakage in Cohort studies.

| **Intervention** | **SUCRA (%)** | **PrBest (%)** | **Mean rank** |
| --- | --- | --- | --- |
| **LC + ERCP (two-stage)** | **91.8** | **59.6** | **1.4** |
| **LC + LTCBDE** | **85.7** | **40.0** | **1.7** |
| **LC + ERCP (one-stage)** | **42.8** | **0.3** | **3.9** |
| **LC + LCBDE + PS** | **41.7** | **0.0** | **3.9** |
| **LC + LCBDE (mixed)** | **27.0** | **0.0** | **4.6** |
| **LC + LCBDE + TTD** | **11.0** | **0.0** | **5.5** |

Abbreviations: SUCRA, surface under the cumulative ranking curve; PrBest, probability of best; LC, laparoscopic cholecystectomy; LCBDE, laparoscopic common bile duct exploration; LTCBDE, laparoscopic transcystic bile duct exploration; ERCP, endoscopic retrograde cholangiopancreatography; PS, primary suture; TTD,T-tube drainage.

**7.9 Postoperative pancreatitis**

**Figure S7.9a:** Cumulative ranking curve plots of different minimally invasive strategies for postoperative pancreatitis in RCTs.

A higher surface under the cumulative ranking curve indicates a higher probability of lower postoperative pancreatitis.

Abbreviations: LC, laparoscopic cholecystectomy; LCBDE, laparoscopic common bile duct exploration; LTCBDE, laparoscopic transcystic bile duct exploration; ERCP, endoscopic retrograde cholangiopancreatography; PS, primary suture;TTD,T-tube drainage.

**Table S7.9a:** SUCRA of the effects of different minimally invasive strategies for postoperative pancreatitis in RCTs.

| **Intervention** | **SUCRA (%)** | **PrBest (%)** | **Mean rank** |
| --- | --- | --- | --- |
| **LC + LCBDE + TTD** | **85.2** | **52.4** | **1.7** |
| **LC + LCBDE + PS** | **77.8** | **27.8** | **2.1** |
| **LC + LCBDE (mixed)** | **52.7** | **3.3** | **3.4** |
| **LC + ERCP (one-stage)** | **36.0** | **2.0** | **4.2** |
| **LC + LTCBDE** | **32.5** | **14.6** | **4.4** |
| **LC + ERCP (two-stage)** | **15.8** | **0.0** | **5.2** |

Abbreviations: SUCRA, surface under the cumulative ranking curve; PrBest, probability of best; LC, laparoscopic cholecystectomy; LCBDE, laparoscopic common bile duct exploration; LTCBDE, laparoscopic transcystic bile duct exploration; ERCP, endoscopic retrograde cholangiopancreatography; PS, primary suture; TTD,T-tube drainage.

**Figure S7.9b:** Cumulative ranking curve plots of different minimally invasive strategies for postoperative pancreatitis in Cohort studies..

A higher surface under the cumulative ranking curve indicates a higher probability of lower postoperative pancreatitis.

Abbreviations: LC, laparoscopic cholecystectomy; LCBDE, laparoscopic common bile duct exploration; LTCBDE, laparoscopic transcystic bile duct exploration; ERCP, endoscopic retrograde cholangiopancreatography; PS, primary suture;TTD,T-tube drainage.

**Table S7.9b:** SUCRA of the effects of different minimally invasive strategies for postoperative pancreatitis in Cohort studies.

| **Intervention** | **SUCRA (%)** | **PrBest (%)** | **Mean rank** |
| --- | --- | --- | --- |
| **LC + LTCBDE** | **82.9** | **47.9** | **1.9** |
| **LC + LCBDE (mixed)** | **80.9** | **33.3** | **2.0** |
| **LC + LCBDE + TTD** | **64.4** | **18.1** | **2.8** |
| **LC + LCBDE + PS** | **44.7** | **0.7** | **3.8** |
| **LC + ERCP (one-stage)** | **24.0** | **0.0** | **4.8** |
| **LC + ERCP (two-stage)** | **3.1** | **0.0** | **5.8** |

Abbreviations: SUCRA, surface under the cumulative ranking curve; PrBest, probability of best; LC, laparoscopic cholecystectomy; LCBDE, laparoscopic common bile duct exploration; LTCBDE, laparoscopic transcystic bile duct exploration; ERCP, endoscopic retrograde cholangiopancreatography; PS, primary suture; TTD,T-tube drainage.

**7.10 Postoperative biliary bleeding**

**Figure S7.10a:** Cumulative ranking curve plots of different minimally invasive strategies for postoperative biliary bleeding in RCTs. A higher surface under the cumulative ranking curve indicates a higher probability of lower postoperative biliary bleeding.

Abbreviations: LC, laparoscopic cholecystectomy; LCBDE, laparoscopic common bile duct exploration; LTCBDE, laparoscopic transcystic bile duct exploration; ERCP, endoscopic retrograde cholangiopancreatography; PS, primary suture;TTD,T-tube drainage.

**Table S7.10a:** SUCRA of the effects of different minimally invasive strategies for postoperative biliary bleeding in RCTs.

| **Intervention** | **SUCRA (%)** | **PrBest (%)** | **Mean rank** |
| --- | --- | --- | --- |
| **LC + LCBDE (mixed)** | **78.5** | **42.1** | **2.1** |
| **LC + LCBDE + PS** | **76.7** | **36.1** | **2.2** |
| **LC + LCBDE + TTD** | **54.5** | **7.1** | **3.3** |
| **LC + LTCBDE** | **35.2** | **14.3** | **4.2** |
| **LC + ERCP (one-stage)** | **29.2** | **0.3** | **4.5** |
| **LC + ERCP (two-stage)** | **25.8** | **0.1** | **4.7** |

Abbreviations: SUCRA, surface under the cumulative ranking curve; PrBest, probability of best; LC, laparoscopic cholecystectomy; LCBDE, laparoscopic common bile duct exploration; LTCBDE, laparoscopic transcystic bile duct exploration; ERCP, endoscopic retrograde cholangiopancreatography; PS, primary suture; TTD,T-tube drainage.

**Figure S7.10b:** Cumulative ranking curve plots of different minimally invasive strategies for postoperative biliary bleeding in Cohort studies. A higher surface under the cumulative ranking curve indicates a higher probability of lower postoperative biliary bleeding.

Abbreviations: LC, laparoscopic cholecystectomy; LCBDE, laparoscopic common bile duct exploration; LTCBDE, laparoscopic transcystic bile duct exploration; ERCP, endoscopic retrograde cholangiopancreatography; PS, primary suture;TTD,T-tube drainage.

**Table S7.10b:** SUCRA of the effects of different minimally invasive strategies for postoperative biliary bleeding in Cohort studies.

| **Intervention** | **SUCRA (%)** | **PrBest (%)** | **Mean rank** |
| --- | --- | --- | --- |
| **LC + LTCBDE** | **79.0** | **59.6** | **2.0** |
| **LC + ERCP (one-stage)** | **62.6** | **22.0** | **2.9** |
| **LC + LCBDE + PS** | **59.0** | **8.5** | **3.0** |
| **LC + LCBDE (mixed)** | **39.7** | **4.4** | **4.0** |
| **LC + LCBDE + TTD** | **33.1** | **5.3** | **4.3** |
| **LC + ERCP (two-stage)** | **26.7** | **0.3** | **4.7** |

Abbreviations: SUCRA, surface under the cumulative ranking curve; PrBest, probability of best; LC, laparoscopic cholecystectomy; LCBDE, laparoscopic common bile duct exploration; LTCBDE, laparoscopic transcystic bile duct exploration; ERCP, endoscopic retrograde cholangiopancreatography; PS, primary suture; TTD,T-tube drainage.

**7.11 Postoperative surgical site infection**

**Figure S7.11a.** Cumulative ranking curve plots of different minimally invasive strategies for postoperative surgical site infection in RCTs. A higher surface under the cumulative ranking curve indicates a higher probability of lower postoperative surgical site infection.

Abbreviations: LC, laparoscopic cholecystectomy; LCBDE, laparoscopic common bile duct exploration; LTCBDE, laparoscopic transcystic bile duct exploration; ERCP, endoscopic retrograde cholangiopancreatography; PS, primary suture;TTD,T-tube drainage.

**Table S7.11a:** SUCRA of the effects of different minimally invasive strategies for postoperative surgical site infection in RCTs.

| **Intervention** | **SUCRA (%)** | **PrBest (%)** | **Mean rank** |
| --- | --- | --- | --- |
| **LC + LCBDE + PS** | 57.6 | **20.1** | **3.1** |
| **LC + LCBDE + TTD** | **56.4** | **24.2** | **3.2** |
| **LC + LTCBDE** | **50.6** | **11.4** | **3.5** |
| **LC + ERCP (one-stage)** | **45.9** | **3.1** | **3.7** |
| **LC + ERCP (two-stage)** | **48.4** | **35.2** | **3.6** |
| **LC + LCBDE (mixed)** | **41.1** | **6.0** | **3.9** |

Abbreviations: SUCRA, surface under the cumulative ranking curve; PrBest, probability of best; LC, laparoscopic cholecystectomy; LCBDE, laparoscopic common bile duct exploration; LTCBDE, laparoscopic transcystic bile duct exploration; ERCP, endoscopic retrograde cholangiopancreatography; PS, primary suture; TTD,T-tube drainage.

**Figure S7.11b:** Cumulative ranking curve plots of different minimally invasive strategies for postoperative surgical site infection in Cohort studies. A higher surface under the cumulative ranking curve indicates a higher probability of lower postoperative surgical site infection.

Abbreviations: LC, laparoscopic cholecystectomy; LCBDE, laparoscopic common bile duct exploration; LTCBDE, laparoscopic transcystic bile duct exploration; ERCP, endoscopic retrograde cholangiopancreatography; PS, primary suture;TTD,T-tube drainage.

**Table S7.11b:** SUCRA of the effects of different minimally invasive strategies for postoperative surgical site infection in Cohort studies.

| **Intervention** | **SUCRA (%)** | **PrBest (%)** | **Mean rank** |
| --- | --- | --- | --- |
| **LC + LTCBDE** | **70.0** | **31.0** | **2.5** |
| **LC + ERCP (two-stage)** | **61.9** | **10.7** | **2.9** |
| **LC + LCBDE (mixed)** | **59.6** | **28.4** | **3.0** |
| **LC + LCBDE + PS** | **54.9** | **25.3** | **3.3** |
| **LC + LCBDE + TTD** | **33.3** | **4.3** | **4.3** |
| **LC + ERCP (one-stage)** | **20.2** | **0.3** | **5.0** |

Abbreviations: SUCRA, surface under the cumulative ranking curve; PrBest, probability of best; LC, laparoscopic cholecystectomy; LCBDE, laparoscopic common bile duct exploration; LTCBDE, laparoscopic transcystic bile duct exploration; ERCP, endoscopic retrograde cholangiopancreatography; PS, primary suture; TTD,T-tube drainage.

**7.12 Postoperative intra-abdominal infection**

**Figure S7.12a:** Cumulative ranking curve plots of different minimally invasive strategies for postoperative intra-abdominal infection in RCTs. A higher surface under the cumulative ranking curve indicates a higher probability of lower postoperative intra-abdominal infection.

Abbreviations: LC, laparoscopic cholecystectomy; LCBDE, laparoscopic common bile duct exploration; LTCBDE, laparoscopic transcystic bile duct exploration; ERCP, endoscopic retrograde cholangiopancreatography; PS, primary suture;TTD,T-tube drainage.

**Table S7.12a:** SUCRA of the effects of different minimally invasive strategies for postoperative intra-abdominal infection in RCTs.

| **Intervention** | **SUCRA (%)** | **PrBest (%)** | **Mean rank** |
| --- | --- | --- | --- |
| **LC + ERCP (one-stage)** | **62.6** | **18.8** | **2.9** |
| **LC + LCBDE (mixed)** | **62.6** | **30.6** | **2.9** |
| **LC + LCBDE + PS** | **51.3** | **15.6** | **3.4** |
| **LC + LTCBDE** | **47.0** | **27.3** | **3.7** |
| **LC + ERCP (two-stage)** | **41.9** | **2.3** | **3.9** |
| **LC + LCBDE + TTD** | **34.7** | **5.4** | **4.3** |

Abbreviations: SUCRA, surface under the cumulative ranking curve; PrBest, probability of best; LC, laparoscopic cholecystectomy; LCBDE, laparoscopic common bile duct exploration; LTCBDE, laparoscopic transcystic bile duct exploration; ERCP, endoscopic retrograde cholangiopancreatography; PS, primary suture; TTD,T-tube drainage.

**Figure S7.12b:** Cumulative ranking curve plots of different minimally invasive strategies for postoperative intra-abdominal infection in Cohort studies. A higher surface under the cumulative ranking curve indicates a higher probability of lower postoperative intra-abdominal infection.

Abbreviations: LC, laparoscopic cholecystectomy; LCBDE, laparoscopic common bile duct exploration; LTCBDE, laparoscopic transcystic bile duct exploration; ERCP, endoscopic retrograde cholangiopancreatography; PS, primary suture;TTD,T-tube drainage.

**Table S7.12b:** SUCRA of the effects of different minimally invasive strategies for postoperative intra-abdominal infection in Cohort studies.

| **Intervention** | **SUCRA (%)** | **PrBest (%)** | **Mean rank** |
| --- | --- | --- | --- |
| **LC + LTCBDE** | **84.2** | **61.2** | **1.8** |
| **LC + ERCP (two-stage)** | **64.6** | **13.6** | **2.8** |
| **LC + LCBDE (mixed)** | **59.5** | **11.3** | **3.0** |
| **LC + LCBDE + PS** | **38.6** | **6.2** | **4.1** |
| **LC + ERCP (one-stage)** | **35.2** | **7.3** | **4.2** |
| **LC + LCBDE + TTD** | **17.9** | **0.5** | **5.1** |

Abbreviations: SUCRA, surface under the cumulative ranking curve; PrBest, probability of best; LC, laparoscopic cholecystectomy; LCBDE, laparoscopic common bile duct exploration; LTCBDE, laparoscopic transcystic bile duct exploration; ERCP, endoscopic retrograde cholangiopancreatography; PS, primary suture; TTD,T-tube drainage.

**7.13 Postoperative cholangitis**

**Figure S7.13a:** Cumulative ranking curve plots of different minimally invasive strategies for Postoperative cholangitis in RCTs. A higher surface under the cumulative ranking curve indicates a higher probability of lower Postoperative cholangitis.

Abbreviations: LC, laparoscopic cholecystectomy; LCBDE, laparoscopic common bile duct exploration; LTCBDE, laparoscopic transcystic bile duct exploration; ERCP, endoscopic retrograde cholangiopancreatography; PS, primary suture;TTD,T-tube drainage.

**Table S7.13a:** SUCRA of the effects of different minimally invasive strategies for postoperative intra-abdominal infection in RCTs.

| **Intervention** | **SUCRA (%)** | **PrBest (%)** | **Mean rank** |
| --- | --- | --- | --- |
| **LC + LTCBDE** | **71.0** | **25.4** | **2.5** |
| **LC + LCBDE + TTD** | **65.6** | **24.8** | **2.7** |
| **LC + LCBDE (mixed)** | **61.5** | **40.4** | **2.9** |
| **LC + LCBDE + PS** | **57.4** | **6.5** | **3.1** |
| **LC + ERCP (two-stage)** | **26.4** | **0.6** | **4.7** |
| **LC + ERCP (one-stage)** | **18.2** | **2.2** | **5.1** |

Abbreviations: SUCRA, surface under the cumulative ranking curve; PrBest, probability of best; LC, laparoscopic cholecystectomy; LCBDE, laparoscopic common bile duct exploration; LTCBDE, laparoscopic transcystic bile duct exploration; ERCP, endoscopic retrograde cholangiopancreatography; PS, primary suture; TTD,T-tube drainage.

**Figure S7.13b:** Cumulative ranking curve plots of different minimally invasive strategies for Postoperative cholangitis in Cohort studies. A higher surface under the cumulative ranking curve indicates a higher probability of lower Postoperative cholangitis.

Abbreviations: LC, laparoscopic cholecystectomy; LCBDE, laparoscopic common bile duct exploration; LTCBDE, laparoscopic transcystic bile duct exploration; ERCP, endoscopic retrograde cholangiopancreatography; PS, primary suture;TTD,T-tube drainage.

**Table S7.13b:** SUCRA of the effects of different minimally invasive strategies for postoperative intra-abdominal infection in Cohort studies.

| **Intervention** | **SUCRA (%)** | **PrBest (%)** | **Mean rank** |
| --- | --- | --- | --- |
| **LC + LTCBDE** | **82.9** | **66.3** | **1.9** |
| **LC + LCBDE (mixed)** | **74.5** | **19.5** | **2.3** |
| **LC + LCBDE + PS** | **55.9** | **3.0** | **3.2** |
| **LC + ERCP (one-stage)** | **49.0** | **9.4** | **3.6** |
| **LC + LCBDE + TTD** | **21.3** | **1.8** | **4.9** |
| **LC + ERCP (two-stage)** | **16.5** | **0.0** | **5.2** |

Abbreviations: SUCRA, surface under the cumulative ranking curve; PrBest, probability of best; LC, laparoscopic cholecystectomy; LCBDE, laparoscopic common bile duct exploration; LTCBDE, laparoscopic transcystic bile duct exploration; ERCP, endoscopic retrograde cholangiopancreatography; PS, primary suture; TTD,T-tube drainage.

**7.14 Symptomatic biliary stricture**

**Figure S7.14a:** Cumulative ranking curve plots of different minimally invasive strategies for symptomatic biliary stricture in RCTs.

A higher surface under the cumulative ranking curve indicates a higher probability of lower symptomatic biliary stricture.

Abbreviations: LC, laparoscopic cholecystectomy; LCBDE, laparoscopic common bile duct exploration; ERCP, endoscopic retrograde cholangiopancreatography; PS, primary suture;TTD,T-tube drainage.

**Table S7.14a:** SUCRA of the effects of different minimally invasive strategies for symptomatic biliary stricture in RCTs.

| **Intervention** | **SUCRA (%)** | **PrBest (%)** | **Mean rank** |
| --- | --- | --- | --- |
| LC + ERCP (two-stage) | **70.4** | **35.4** | **1.9** |
| LC + LCBDE (mixed) | **65.4** | **45.5** | **2.0** |
| LC + LCBDE + PS | **34.0** | **14.0** | **3.0** |
| LC + LCBDE + TTD | **30.2** | **5.1** | **3.1** |

Abbreviations: SUCRA, surface under the cumulative ranking curve; PrBest, probability of best; LC, laparoscopic cholecystectomy; LCBDE, laparoscopic common bile duct exploration; ERCP, endoscopic retrograde cholangiopancreatography; PS, primary suture; TTD,T-tube drainage.

**Figure S7.14b:** Cumulative ranking curve plots of different minimally invasive strategies for symptomatic biliary stricture in Cohort studies.

A higher surface under the cumulative ranking curve indicates a higher probability of lower symptomatic biliary stricture.

Abbreviations: LC, laparoscopic cholecystectomy; LCBDE, laparoscopic common bile duct exploration; ERCP, endoscopic retrograde cholangiopancreatography; PS, primary suture;TTD,T-tube drainage.

**Table S7.14b:** SUCRA of the effects of different minimally invasive strategies for symptomatic biliary stricture in Cohort studies.

| **Intervention** | **SUCRA (%)** | **PrBest (%)** | **Mean rank** |
| --- | --- | --- | --- |
| **LC + ERCP (two-stage)** | **69.7** | **22.8** | **2.2** |
| **LC + ERCP (one-stage)** | **57.0** | **33.9** | **2.7** |
| **LC + LCBDE (mixed)** | **57.1** | **29.6** | **2.7** |
| **LC + LCBDE + PS** | **46.9** | **12.0** | **3.1** |
| LC + LCBDE + TTD | **19.2** | **1.6** | **4.2** |

Abbreviations: SUCRA, surface under the cumulative ranking curve; PrBest, probability of best; LC, laparoscopic cholecystectomy; LCBDE, laparoscopic common bile duct exploration; ERCP, endoscopic retrograde cholangiopancreatography; PS, primary suture; TTD,T-tube drainage.

**7.15 Stone recurrence rate**

**Figure S7.15a:** Cumulative ranking curve plots of different minimally invasive strategies for stone recurrence in RCTs.

A higher surface under the cumulative ranking curve indicates a higher probability of lower stone recurrence.

Abbreviations: LC, laparoscopic cholecystectomy; LCBDE, laparoscopic common bile duct exploration; LTCBDE, laparoscopic transcystic bile duct exploration; ERCP, endoscopic retrograde cholangiopancreatography; PS, primary suture;TTD,T-tube drainage.

**Table S7.15a:** SUCRA of the effects of different minimally invasive strategies for stone recurrence in RCTs.

| **Intervention** | **SUCRA (%)** | **PrBest (%)** | **Mean rank** |
| --- | --- | --- | --- |
| **LC + LCBDE + TTD** | **69.9** | **24.4** | **2.5** |
| **LC + ERCP (one-stage)** | **66.3** | **34.2** | **2.7** |
| **LC + LCBDE + PS** | **58.8** | **17.3** | **3.1** |
| **LC + LTCBDE** | **43.9** | **21.2** | **3.8** |
| **LC + ERCP (two-stage)** | **39.4** | **1.8** | **4.0** |
| **LC + LCBDE (mixed)** | **21.7** | **1.0** | **4.9** |

Abbreviations: SUCRA, surface under the cumulative ranking curve; PrBest, probability of best; LC, laparoscopic cholecystectomy; LCBDE, laparoscopic common bile duct exploration; LTCBDE, laparoscopic transcystic bile duct exploration; ERCP, endoscopic retrograde cholangiopancreatography; PS, primary suture; TTD,T-tube drainage.

**Figure S7.15b:** Cumulative ranking curve plots of different minimally invasive strategies for stone recurrence in Cohort studies.

A higher surface under the cumulative ranking curve indicates a higher probability of lower stone recurrence.

Abbreviations: LC, laparoscopic cholecystectomy; LCBDE, laparoscopic common bile duct exploration; LTCBDE, laparoscopic transcystic bile duct exploration; ERCP, endoscopic retrograde cholangiopancreatography; PS, primary suture;TTD,T-tube drainage.

**Table S7.15b:** SUCRA of the effects of different minimally invasive strategies for stone recurrence in Cohort studies.

| **Intervention** | **SUCRA (%)** | **PrBest (%)** | **Mean rank** |
| --- | --- | --- | --- |
| **LC + LTCBDE** | **93.2** | **84.0** | **1.3** |
| **LC + LCBDE (mixed)** | **58.3** | **6.0** | **3.1** |
| **LC + LCBDE + TTD** | **56.8** | **5.4** | **3.2** |
| **LC + LCBDE + PS** | **48.1** | **1.3** | **3.6** |
| **LC + ERCP (one-stage)** | **39.2** | **3.4** | **4.0** |
| **LC + ERCP (two-stage)** | **4.3** | **0.0** | **5.8** |

Abbreviations: SUCRA, surface under the cumulative ranking curve; PrBest, probability of best; LC, laparoscopic cholecystectomy; LCBDE, laparoscopic common bile duct exploration; LTCBDE, laparoscopic transcystic bile duct exploration; ERCP, endoscopic retrograde cholangiopancreatography; PS, primary suture; TTD,T-tube drainage.

# Appendix 8: League table of summary effect estimates

**8.1. Single-session stone clearance rate**

**Table S8.1a:** League table of single-session stone clearance rate in RCTs. The columns represent the comparison of the intervention listed in the row to the intervention listed in the column. The rows represent the comparison of the intervention listed in the row to the intervention listed in the column. Effect estimates are expressed as risk ratios (RRs) with 95% confidence intervals (CIs). For example, the RR for single-session stone clearance for LC + LCBDE + TTD compared with LC + LCBDE + PS is 1.01 (95% Cl 1.00 to 1.08). An RR greater than 1 favors the intervention listed in the row, whereas an RR less than 1 favors the intervention listed in the column.

| LC + LCBDE + PS |  |  |  |  |  |
| --- | --- | --- | --- | --- | --- |
| 1.02 (0.96,1.08) | LC + ERCP (one-stage) |  |  |  |  |
| 1.05 (1.00,1.10) | 1.03 (1.00,1.06) | LC + ERCP (two-stage) |  |  |  |
| 1.01 (0.94,1.09) | 1.00 (0.91,1.09) | 0.97 (0.89,1.05) | LC + LTCBDE |  |  |
| 1.04 (0.99,1.09) | 1.02 (0.99,1.05) | 0.99 (0.97,1.01) | 1.02 (0.94,1.12) | LC + LCBDE (mixed) |  |
| 1.01 (0.99,1.04) | 0.99 (0.94,1.05) | 0.97 (0.92,1.01) | 1.00 (0.92,1.08) | 0.98 (0.93,1.03) | LC + LCBDE + TTD |

**Table S8.1b:** League table of single-session stone clearance rate in Cohort studies. The columns represent the comparison of the intervention listed in the row to the intervention listed in the column. The rows represent the comparison of the intervention listed in the row to the intervention listed in the column. Effect estimates are expressed as RRs with 95% CIs. For example, the RR for single-session stone clearance for LC + LCBDE + TTD compared with LC + LCBDE + PS is 1.01 (95% Cl 0.97 to 1.05). An RR greater than 1 favors the intervention listed in the row, whereas an RR less than 1 favors the intervention listed in the column.

| LC + LCBDE + PS |  |  |  |  |  |
| --- | --- | --- | --- | --- | --- |
| 0.98 (0.92,1.05) | LC + ERCP (one-stage) |  |  |  |  |
| 1.05 (1.01,1.09) | 1.06 (1.01,1.13) | LC + ERCP (two-stage) |  |  |  |
| 1.01 (0.96,1.06) | 1.03 (0.95,1.10) | 0.96 (0.92,1.01) | LC + LTCBDE |  |  |
| 1.00 (0.95,1.05) | 1.02 (0.96,1.08) | 0.95 (0.92,0.99) | 0.99 (0.93,1.05) | LC + LCBDE (mixed) |  |
| 1.01 (0.97,1.05) | 1.03 (0.96,1.10) | 0.96 (0.92,1.01) | 1.00 (0.94,1.06) | 1.01 (0.95,1.07) | LC + LCBDE + TTD |

**8.2 Severe postoperative complications.**

**Table S8.2a:** League table of severe postoperative complications in RCTs. The columns represent the comparison of the intervention listed in the row to the intervention listed in the column. The rows represent the comparison of the intervention listed in the row to the intervention listed in the column. Effect estimates are expressed as ORs with 95% CIs. For example, the OR for severe postoperative complications for LC + LCBDE + TTD compared with LC + LCBDE + PS is 0.43 (95% Cl 0.16 to 1.14). An OR greater than 1 indicates higher odds of the Severe postoperative complications for the intervention listed in the row, whereas an OR less than 1 indicates lower odds.

| LC + LCBDE + PS |  |  |  |  |  |
| --- | --- | --- | --- | --- | --- |
| 1.60 (0.38,6.71) | LC + ERCP (one-stage) |  |  |  |  |
| 0.53 (0.19,1.48) | 0.33 (0.11,1.02) | LC + ERCP (two-stage) |  |  |  |
| 0.55 (0.01,32.18) | 0.35 (0.01,20.68) | 1.04 (0.02,53.12) | LC + LTCBDE |  |  |
| 1.50 (0.30,7.43) | 0.94 (0.20,4.29) | 2.81 (0.80,9.85) | 2.71 (0.04,169.09) | LC + LCBDE (mixed) |  |
| 0.43 (0.16,1.14) | 0.27 (0.05,1.48) | 0.81 (0.21,3.16) | 0.78 (0.01,50.35) | 0.29 (0.05,1.81) | LC + LCBDE + TTD |

**Table S8.2b:** League table of severe postoperative complications in Cohort studies. The columns represent the comparison of the intervention listed in the row to the intervention listed in the column. The rows represent the comparison of the intervention listed in the row to the intervention listed in the column. Effect estimates are expressed as ORs with 95% CIs. For example, the OR for severe postoperative complications for LC + LCBDE + TTD compared with LC + LCBDE + PS is 1.55 (95% Cl 0.49 to 4.96). An OR greater than 1 indicates higher odds of the Severe postoperative complications for the intervention listed in the row, whereas an OR less than 1 indicates lower odds.

| LC + LCBDE + PS |  |  |  |  |  |
| --- | --- | --- | --- | --- | --- |
| 1.20 (0.25,5.65) | LC + ERCP (one-stage) |  |  |  |  |
| 1.11 (0.46,2.66) | 0.92 (0.26,3.32) | LC + ERCP (two-stage) |  |  |  |
| 1.36 (0.30,6.20) | 1.13 (0.19,6.72) | 1.22 (0.35,4.24) | LC + LTCBDE |  |  |
| 1.71 (0.61,4.78) | 1.43 (0.40,5.05) | 1.54 (0.90,2.64) | 1.26 (0.33,4.88) | LC + LCBDE (mixed) |  |
| 1.55 (0.49,4.96) | 1.30 (0.26,6.46) | 1.40 (0.53,3.71) | 1.15 (0.24,5.55) | 0.91 (0.30,2.76) | LC + LCBDE + TTD |

**8.3 Operative time**

**Table S8.3a:** League table of operative time in RCTs. The columns represent the comparison of the intervention listed in the row to the intervention listed in the column. The rows represent the comparison of the intervention listed in the row to the intervention listed in the column. The effect estimates are expressed as MDs with 95% CIs. For example, the OR for severe postoperative complications for LC + LCBDE + TTD compared with LC + LCBDE + PS is -20.02 (95% Cl -33.26 to -6.78). A mean difference < 0 favors the intervention listed in the column, whereas a mean difference > 0 favors the intervention listed in the row.

| LC + LCBDE + PS |  |  |  |  |  |
| --- | --- | --- | --- | --- | --- |
| -4.17 (-23.97,15.64) | LC + ERCP (one-stage) |  |  |  |  |
| 16.35  (0.07,32.62) | 20.51  (9.15,31.88) | LC + ERCP (two-stage) |  |  |  |
| 26.99  (6.70,47.28) | 31.16  (6.61,55.70) | 10.64 (-11.15,32.44) | LC + LTCBDE |  |  |
| -8.70 (-31.46,14.06) | -4.53  (-20.54,11.48) | -25.05 (-41.12,-8.98) | -35.69 (-62.68,-8.70) | LC + LCBDE (mixed) |  |
| -20.02 (-33.26,-6.78) | -15.85  (-39.68,7.98) | -36.37 (-57.33,-15.40) | -47.01 (-71.23,-22.78) | -11.32 (-37.69,15.05) | LC + LCBDE + TTD |

**Table S8.3b:** League table of operative time in Cohort studies. The columns represent the comparison of the intervention listed in the row to the intervention listed in the column. The rows represent the comparison of the intervention listed in the row to the intervention listed in the column. The effect estimates are expressed as MDs with 95% CIs. For example, the OR for severe postoperative complications for LC + LCBDE + TTD compared with LC + LCBDE + PS is -3.40 (95% Cl -26.59 to 19.80). A mean difference < 0 favors the intervention listed in the column, whereas a mean difference > 0 favors the intervention listed in the row.

| LC + LCBDE + PS |  |  |  |  |  |
| --- | --- | --- | --- | --- | --- |
| -14.09 (-44.11,15.93) | LC + ERCP (one-stage) |  |  |  |  |
| 7.84  (-16.64,32.33) | 21.93  (-0.53,44.38) | LC + ERCP (two-stage) |  |  |  |
| 25.34  (-7.72,58.40) | 39.43  (1.66,77.20) | 17.50 (-16.00,51.00) | LC + LTCBDE |  |  |
| -14.12 (-45.10,16.86) | -0.03 (-25.54,25.48) | -21.96 (-44.58,0.66) | -39.46 (-75.19,-3.73) | LC + LCBDE (mixed) |  |
| -3.40  (-26.59,19.80) | 10.69 (-22.65,44.03) | -11.24 (-38.69,16.22) | -28.74  (-64.39,6.92) | 10.72 (-22.99,44.44) | LC + LCBDE + TTD |

**8.4 Total length of hospital stay**

**Table S8.4a:** League table of total length of hospital stay in RCTs. The columns represent the comparison of the intervention listed in the row to the intervention listed in the column. The rows represent the comparison of the intervention listed in the row to the intervention listed in the column. The effect estimates are expressed as MDs with 95% CIs. For example, the OR for severe postoperative complications for LC + LCBDE + TTD compared with LC + LTCBDE is -5.78 (95% Cl -7.96 to -2.42). A mean difference < 0 favors the intervention listed in the column, whereas a mean difference > 0 favors the intervention listed in the row.

| LC + LCBDE + PS |  |  |  |  |  |
| --- | --- | --- | --- | --- | --- |
| 1.98 (-0.16,4.13) | LC + ERCP (one-stage) |  |  |  |  |
| -0.20 (-1.77,1.38) | -2.18 (-3.65,-0.71) | LC + ERCP (two-stage) |  |  |  |
| 2.75 (0.42,5.08) | 0.77 (-2.01,3.55) | 2.95 (0.59,5.31) | LC + LTCBDE |  |  |
| 2.16 (-0.26,4.58) | 0.17 (-1.83,2.18) | 2.35 (0.51,4.20) | -0.59 (-3.59,2.40) | LC + LCBDE (mixed) |  |
| -3.03 (-4.51,-1.55) | -5.02 (-7.54,-2.49) | -2.84 (-4.90,-0.77) | -5.78 (-8.51,-3.05) | -5.19 (-7.96,-2.42) | LC + LCBDE + TTD |

**Table S8.4b:** League table of total length of hospital stay in Cohort studies. The columns represent the comparison of the intervention listed in the row to the intervention listed in the column. The rows represent the comparison of the intervention listed in the row to the intervention listed in the column. The effect estimates are expressed as MDs with 95% CIs. For example, the OR for severe postoperative complications for LC + LCBDE + TTD compared with LC + LTCBDE is -3.78 (95% Cl -6.27 to -1.29). A mean difference < 0 favors the intervention listed in the column, whereas a mean difference > 0 favors the intervention listed in the row.

| LC + LCBDE + PS |  |  |  |  |  |
| --- | --- | --- | --- | --- | --- |
| 0.45 (-1.70,2.59) | LC + ERCP (one-stage) |  |  |  |  |
| -2.85 (-4.46,-1.23) | -3.30 (-4.98,-1.61) | LC + ERCP (two-stage) |  |  |  |
| 1.42 (-0.86,3.70) | 0.97 (-1.66,3.60) | 4.27 (2.09,6.44) | LC + LTCBDE |  |  |
| -0.49 (-2.53,1.56) | -0.94 (-2.77,0.90) | 2.36 (0.93,3.79) | -1.91 (-4.30,0.49) | LC + LCBDE (mixed) |  |
| -2.36 (-4.04,-0.68) | -2.81 (-5.23,-0.39) | 0.49 (-1.40,2.38) | -3.78 (-6.27,-1.29) | -1.87 (-4.16,0.42) | LC + LCBDE + TTD |

**8.5 Intraoperative blood loss**

**Table S8.5:** League table of intraoperative blood loss in Cohort studies. The columns represent the comparison of the intervention listed in the row to the intervention listed in the column. The rows represent the comparison of the intervention listed in the row to the intervention listed in the column. The effect estimates are expressed as MDs with 95% CIs. For example, the OR for severe postoperative complications for LC + LCBDE + TTD compared with LC + LTCBDE is -12.95 (95% Cl -25.16 to -0.74). A mean difference < 0 favors the intervention listed in the column, whereas a mean difference > 0 favors the intervention listed in the row.

| LC + LCBDE + PS |  |  |  |  |  |
| --- | --- | --- | --- | --- | --- |
| 6.93 (-7.22,21.09) | LC + ERCP (one-stage) |  |  |  |  |
| 1.83 (-5.27,8.94) | -5.10 (-17.35,7.15) | LC + ERCP (two-stage) |  |  |  |
| 16.80 (5.96,27.64) | 9.87 (-6.79,26.53) | 14.97 (3.68,26.26) | LC + LTCBDE |  |  |
| 10.40 (-4.08,24.89) | 3.47 (-15.68,22.61) | 8.57 (-6.15,23.28) | -6.40 (-16.95,4.15) | LC + LCBDE (mixed) |  |
| 3.85 (-2.89,10.58) | -3.08 (-17.71,11.54) | 2.01 (-5.99,10.02) | -12.95 (-25.16,-0.74) | -6.55 (-21.97,8.86) | LC + LCBDE + TTD |

**8.6 Conversion to open surgery.**

**Table S8.6a:** League table of conversion to open surgery in RCTs. The columns represent the comparison of the intervention listed in the row to the intervention listed in the column. The rows represent the comparison of the intervention listed in the row to the intervention listed in the column. Effect estimates are expressed as ORs with 95% CIs. For example, the OR for severe postoperative complications for LC + LCBDE + TTD compared with LC + LTCBDE is 0.88 (95% Cl 0.07 to 10.30). An OR greater than 1 indicates higher odds of the Conversion to open surgery for the intervention listed in the row, whereas an OR less than 1 indicates lower odds.

| LC + LCBDE + PS |  |  |  |  |  |
| --- | --- | --- | --- | --- | --- |
| 0.73 (0.20,2.67) | LC + ERCP (one-stage) |  |  |  |  |
| 0.77 (0.28,2.11) | 1.06 (0.46,2.42) | LC + ERCP (two-stage) |  |  |  |
| 1.04 (0.11,10.09) | 1.43 (0.12,17.17) | 1.35 (0.13,14.07) | LC + LTCBDE |  |  |
| 0.69 (0.18,2.63) | 0.95 (0.35,2.56) | 0.89 (0.37,2.17) | 0.66 (0.05,8.16) | LC + LCBDE (mixed) |  |
| 0.91 (0.31,2.69) | 1.25 (0.30,5.26) | 1.18 (0.36,3.83) | 0.88 (0.07,10.30) | 1.32 (0.30,5.75) | LC + LCBDE + TTD |

**Table S8.6b:** League table of conversion to open surgery in Cohort studies. The columns represent the comparison of the intervention listed in the row to the intervention listed in the column. The rows represent the comparison of the intervention listed in the row to the intervention listed in the column. Effect estimates are expressed as ORs with 95% CIs. For example, the OR for severe postoperative complications for LC + LCBDE + TTD compared with LC + LTCBDE is 0.82 (95% Cl 0.28 to 2.40). An OR greater than 1 indicates higher odds of the Conversion to open surgery for the intervention listed in the row, whereas an OR less than 1 indicates lower odds.

| LC + LCBDE + PS |  |  |  |  |  |
| --- | --- | --- | --- | --- | --- |
| 1.25 (0.44,3.49) | LC + ERCP (one-stage) |  |  |  |  |
| 0.87 (0.42,1.83) | 0.70 (0.33,1.47) | LC + ERCP (two-stage) |  |  |  |
| 1.05 (0.34,3.23) | 0.84 (0.20,3.53) | 1.20 (0.35,4.14) | LC + LTCBDE |  |  |
| 1.42 (0.58,3.48) | 1.14 (0.61,2.15) | 1.63 (0.98,2.72) | 1.36 (0.36,5.17) | LC + LCBDE (mixed) |  |
| 0.86 (0.33,2.27) | 0.69 (0.20,2.44) | 0.99 (0.35,2.76) | 0.82 (0.28,2.40) | 0.60 (0.19,1.91) | LC + LCBDE + TTD |

**8.7 Reintervention.**

**Table S8.7a:** League table of reintervention in RCTs. The columns represent the comparison of the intervention listed in the row to the intervention listed in the column. The rows represent the comparison of the intervention listed in the row to the intervention listed in the column. Effect estimates are expressed as ORs with 95% CIs. For example, the OR for severe postoperative complications for LC + LCBDE + TTD compared with LC + LTCBDE is 0.82 (95% Cl 0.28 to 2.40). An OR greater than 1 indicates higher odds of reintervention in the intervention listed in the row,whereas an OR less than 1 indicates lower odds.

| LC + LCBDE + PS |  |  |  |  |  |
| --- | --- | --- | --- | --- | --- |
| 1.25 (0.44,3.49) | LC + ERCP (one-stage) |  |  |  |  |
| 0.87 (0.42,1.83) | 0.70 (0.33,1.47) | LC + ERCP (two-stage) |  |  |  |
| 1.05 (0.34,3.23) | 0.84 (0.20,3.53) | 1.20 (0.35,4.14) | LC + LTCBDE |  |  |
| 1.42 (0.58,3.48) | 1.14 (0.61,2.15) | 1.63 (0.98,2.72) | 1.36 (0.36,5.17) | LC + LCBDE (mixed) |  |
| 0.86 (0.33,2.27) | 0.69 (0.20,2.44) | 0.99 (0.35,2.76) | 0.82 (0.28,2.40) | 0.60 (0.19,1.91) | LC + LCBDE + TTD |

**Table S8.7b:** League table of reintervention in Cohort studies. The columns represent the comparison of the intervention listed in the row to the intervention listed in the column. The rows represent the comparison of the intervention listed in the row to the intervention listed in the column. Effect estimates are expressed as ORs with 95% CIs. For example, the OR for severe postoperative complications for LC + LCBDE + TTD compared with LC + LTCBDE is 0.82 (95% Cl 0.28 to 2.40). An OR greater than 1 indicates higher odds of reintervention in the intervention listed in the row,whereas an OR less than 1 indicates lower odds.

| LC + LCBDE + PS |  |  |  |  |  |
| --- | --- | --- | --- | --- | --- |
| 1.25 (0.44,3.49) | LC + ERCP (one-stage) |  |  |  |  |
| 0.87 (0.42,1.83) | 0.70 (0.33,1.47) | LC + ERCP (two-stage) |  |  |  |
| 1.05 (0.34,3.23) | 0.84 (0.20,3.53) | 1.20 (0.35,4.14) | LC + LTCBDE |  |  |
| 1.42 (0.58,3.48) | 1.14 (0.61,2.15) | 1.63 (0.98,2.72) | 1.36 (0.36,5.17) | LC + LCBDE (mixed) |  |
| 0.86 (0.33,2.27) | 0.69 (0.20,2.44) | 0.99 (0.35,2.76) | 0.82 (0.28,2.40) | 0.60 (0.19,1.91) | LC + LCBDE + TTD |

**8.8 Postoperative bile leakage**

**Table S8.8a:** League table of postoperative bile leakage in RCTs. The columns represent the comparison of the intervention listed in the row to the intervention listed in the column. The rows represent the comparison of the intervention listed in the row to the intervention listed in the column. Effect estimates are expressed as odds ratios (ORs) with 95% CIs. For example, the OR for severe postoperative complications for LC + LCBDE + TTD compared with LC + LCBDE + PS is 1.07 (95% Cl 0.52 to 2.17). An OR greater than 1 indicates higher odds of postoperative bile leakage in the intervention listed in the row,whereas an OR less than 1 indicates lower odds.

| LC + LCBDE + PS |  |  |  |  |  |
| --- | --- | --- | --- | --- | --- |
| 2.23 (0.43,11.56) | LC + ERCP (one-stage) |  |  |  |  |
| 2.48 (0.81,7.55) | 1.11 (0.33,3.71) | LC + ERCP (two-stage) |  |  |  |
| 2.39 (0.04,138.48) | 1.07 (0.02,63.63) | 0.97 (0.02,47.83) | LC + LTCBDE |  |  |
| 0.40 (0.08,1.97) | 0.18 (0.05,0.67) | 0.16 (0.05,0.51) | 0.17 (0.00,9.73) | LC + LCBDE (mixed) |  |
| 1.07 (0.52,2.17) | 0.48 (0.09,2.65) | 0.43 (0.13,1.45) | 0.45 (0.01,26.57) | 2.68 (0.50,14.22) | LC + LCBDE + TTD |

**Table S8.8b:** League table of postoperative bile leakage in Cohort studies. The columns represent the comparison of the intervention listed in the row to the intervention listed in the column. The rows represent the comparison of the intervention listed in the row to the intervention listed in the column. Effect estimates are expressed as odds ratios (ORs) with 95% CIs. For example, the OR for severe postoperative complications for LC + LCBDE + TTD compared with LC + LTCBDE is 0.22 (95% Cl 0.09 to 0.59). An OR greater than 1 indicates higher odds of postoperative bile leakage in the intervention listed in the row,whereas an OR less than 1 indicates lower odds.

| LC + LCBDE + PS |  |  |  |  |  |
| --- | --- | --- | --- | --- | --- |
| 1.04 (0.35,3.13) | LC + ERCP (one-stage) |  |  |  |  |
| 2.98 (1.30,6.83) | 2.85 (1.28,6.33) | LC + ERCP (two-stage) |  |  |  |
| 2.58 (1.08,6.17) | 2.47 (0.72,8.49) | 0.87 (0.32,2.36) | LC + LTCBDE |  |  |
| 0.80 (0.28,2.27) | 0.76 (0.29,2.01) | 0.27 (0.13,0.56) | 0.31 (0.10,0.96) | LC + LCBDE (mixed) |  |
| 0.58 (0.28,1.18) | 0.55 (0.16,1.88) | 0.19 (0.07,0.52) | 0.22 (0.09,0.59) | 0.72 (0.22,2.32) | LC + LCBDE + TTD |

**8.9 Postoperative pancreatitis**

**Table S8.9a:** League table of postoperative pancreatitis in RCTs. The columns represent the comparison of the intervention listed in the row to the intervention listed in the column. The rows represent the comparison of the intervention listed in the row to the intervention listed in the column. Effect estimates are expressed as ORs with 95% CIs. For example, the OR for severe postoperative complications for LC + LCBDE + TTD compared with LC + LTCBDE is 6.92 (95% Cl 0.52 to 18.62). An OR greater than 1 indicates higher odds of postoperative pancreatitis in the intervention listed in the row,whereas an OR less than 1 indicates lower odds.

| LC + LCBDE + PS |  |  |  |  |  |
| --- | --- | --- | --- | --- | --- |
| 0.29 (0.05,1.80) | LC + ERCP (one-stage) |  |  |  |  |
| 0.19 (0.04,0.84) | 0.67 (0.23,2.02) | LC + ERCP (two-stage) |  |  |  |
| 0.19 (0.00,12.07) | 0.65 (0.01,37.50) | 0.97 (0.02,47.83) | LC + LTCBDE |  |  |
| 0.41 (0.08,2.02) | 1.45 (0.45,4.62) | 2.15 (1.20,3.84) | 2.22 (0.04,114.91) | LC + LCBDE (mixed) |  |
| 1.29 (0.29,5.72) | 4.51 (0.60,33.78) | 6.68 (1.23,36.24) | 6.92 (0.10,486.72) | 3.12 (0.52,18.62) | LC + LCBDE + TTD |

**Table S8.9b:** League table of postoperative pancreatitis in Cohort studies. The columns represent the comparison of the intervention listed in the row to the intervention listed in the column. The rows represent the comparison of the intervention listed in the row to the intervention listed in the column. Effect estimates are expressed as ORs with 95% CIs. For example, the OR for severe postoperative complications for LC + LCBDE + TTD compared with LC + LTCBDE is 0.59 (95% Cl 0.08 to 4.13). An OR greater than 1 indicates higher odds of postoperative pancreatitis in the intervention listed in the row,whereas an OR less than 1 indicates lower odds.

| LC + LCBDE + PS |  |  |  |  |  |
| --- | --- | --- | --- | --- | --- |
| 0.59 (0.19,1.86) | LC + ERCP (one-stage) |  |  |  |  |
| 0.35 (0.14,0.84) | 0.59 (0.24,1.42) | LC + ERCP (two-stage) |  |  |  |
| 2.83 (0.61,13.27) | 4.81 (0.94,24.67) | 8.19 (1.93,34.71) | LC + LTCBDE |  |  |
| 2.54 (0.77,8.37) | 4.31 (1.52,12.17) | 7.34 (3.12,17.23) | 0.90 (0.19,4.23) | LC + LCBDE (mixed) |  |
| 1.68 (0.35,7.96) | 2.85 (0.51,15.81) | 4.85 (1.07,21.93) | 0.59 (0.08,4.13) | 0.66 (0.12,3.66) | LC + LCBDE + TTD |

**8.10 Postoperative biliary bleeding**

**Table S8.10a:** League table of postoperative biliary bleeding in RCTs. The columns represent the comparison of the intervention listed in the row to the intervention listed in the column. The rows represent the comparison of the intervention listed in the row to the intervention listed in the column. Effect estimates are expressed as ORs with 95% CIs. For example, the OR for severe postoperative complications for LC + LCBDE + TTD compared with LC + LCBDE + PS is 0.48 (95% Cl 0.09 to 2.74). An OR greater than 1 indicates higher odds of postoperative biliary bleeding in the intervention listed in the row,whereas an OR less than 1 indicates lower odds.

| LC + LCBDE + PS |  |  |  |  |  |
| --- | --- | --- | --- | --- | --- |
| 0.21 (0.02,2.69) | LC + ERCP (one-stage) |  |  |  |  |
| 0.20 (0.02,1.80) | 0.96 (0.26,3.54) | LC + ERCP (two-stage) |  |  |  |
| 0.19 (0.00,17.55) | 0.92 (0.01,58.59) | 0.97 (0.02,49.49) | LC + LTCBDE |  |  |
| 1.07 (0.06,20.32) | 5.11 (0.82,31.94) | 5.33 (0.75,38.01) | 5.52 (0.07,449.83) | LC + LCBDE (mixed) |  |
| 0.48 (0.09,2.74) | 2.31 (0.22,24.32) | 2.41 (0.34,17.07) | 2.50 (0.03,202.80) | 0.45 (0.03,7.24) | LC + LCBDE + TTD |

**Table S8.10b:** League table of postoperative biliary bleeding in Cohort studies. The columns represent the comparison of the intervention listed in the row to the intervention listed in the column. The rows represent the comparison of the intervention listed in the row to the intervention listed in the column. Effect estimates are expressed as ORs with 95% CIs. For example, the OR for severe postoperative complications for LC + LCBDE + TTD compared with LC + LTCBDE is 0.27 (95% Cl 0.02 to 4.08). An OR greater than 1 indicates higher odds of postoperative biliary bleeding in the intervention listed in the row,whereas an OR less than 1 indicates lower odds.

| LC + LCBDE + PS |  |  |  |  |  |
| --- | --- | --- | --- | --- | --- |
| 1.12 (0.21,5.91) | LC + ERCP (one-stage) |  |  |  |  |
| 0.60 (0.21,1.69) | 0.54 (0.12,2.39) | LC + ERCP (two-stage) |  |  |  |
| 2.26 (0.20,25.11) | 2.01 (0.13,31.03) | 3.76 (0.37,38.55) | LC + LTCBDE |  |  |
| 0.71 (0.18,2.89) | 0.64 (0.12,3.36) | 1.19 (0.45,3.13) | 0.32 (0.03,3.92) | LC + LCBDE (mixed) |  |
| 0.61 (0.15,2.43) | 0.54 (0.07,4.34) | 1.01 (0.21,4.95) | 0.27 (0.02,4.08) | 0.85 (0.13,5.41) | LC + LCBDE + TTD |

**8.11: Postoperative surgical site infection**

**Table S8.11a:** league table of postoperative surgical site infection in RCTs. The columns represent the comparison of the intervention listed in the row to the intervention listed in the column. The rows represent the comparison of the intervention listed in the row to the intervention listed in the column. Effect estimates are expressed as ORs with 95% CIs. For example, the OR for severe postoperative complications for LC + LCBDE + TTD compared with LC + ERCP (one-stage) is 0.86 (95% Cl 0.12 to 6.04). An OR greater than 1 indicates higher odds of postoperative surgical site infection in the intervention listed in the row,whereas an OR less than 1 indicates lower odds.

| LC + LCBDE + PS |  |  |  |  |  |
| --- | --- | --- | --- | --- | --- |
| 0.99 (0.13,7.86) | LC + ERCP (one-stage) |  |  |  |  |
| 0.79 (0.20,3.05) | 0.79 (0.17,3.79) | LC + ERCP (two-stage) |  |  |  |
| 0.76 (0.01,47.24) | 0.77 (0.01,51.25) | 0.97 (0.02,47.83) | LC + LTCBDE |  |  |
| 0.73 (0.13,4.01) | 0.74 (0.14,3.77) | 0.93 (0.33,2.60) | 0.96 (0.02,54.55) | LC + LCBDE (mixed) |  |
| 0.85 (0.23,3.11) | 0.86 (0.12,6.04) | 1.09 (0.34,3.47) | 1.13 (0.02,66.02) | 1.17 (0.25,5.51) | LC + LCBDE + TTD |

**Table S8.11b:** league table of postoperative surgical site infection in Cohort studies. The columns represent the comparison of the intervention listed in the row to the intervention listed in the column. The rows represent the comparison of the intervention listed in the row to the intervention listed in the column. Effect estimates are expressed as ORs with 95% CIs. For example, the OR for severe postoperative complications for LC + LCBDE + TTD compared with LC + LTCBDE is 0.34 (95% Cl 0.10 to 1.16). An OR greater than 1 indicates higher odds of postoperative surgical site infection in the intervention listed in the row,whereas an OR less than 1 indicates lower odds.

| LC + LCBDE + PS |  |  |  |  |  |
| --- | --- | --- | --- | --- | --- |
| 2.08 (0.15,27.86) | LC + ERCP (one-stage) |  |  |  |  |
| 2.05 (0.32,13.02) | 0.99 (0.16,6.11) | LC + ERCP (two-stage) |  |  |  |
| 2.35 (0.54,10.29) | 1.13 (0.09,13.56) | 1.15 (0.21,6.19) | LC + LTCBDE |  |  |
| 1.84 (0.13,27.05) | 0.89 (0.06,12.79) | 0.90 (0.13,6.32) | 0.78 (0.06,10.29) | LC + LCBDE (mixed) |  |
| 0.81 (0.26,2.51) | 0.39 (0.03,4.48) | 0.39 (0.08,2.01) | 0.34 (0.10,1.16) | 0.44 (0.03,5.56) | LC + LCBDE + TTD |

**8.12 Postoperative intra-abdominal infection**

**Table S8.12a:** league table of postoperative intra-abdominal infection in RCTs. The columns represent the comparison of the intervention listed in the row to the intervention listed in the column. The rows represent the comparison of the intervention listed in the row to the intervention listed in the column. Effect estimates are expressed as ORs with 95% CIs. For example, the OR for severe postoperative complications for LC + LCBDE + TTD compared with LC + LCBDE + PS is 0.68 (95% Cl 0.20 to 2.39). An OR greater than 1 indicates higher odds of postoperative surgical site infection in the intervention listed in the row,whereas an OR less than 1 indicates lower odds.

| LC + LCBDE + PS |  |  |  |  |  |
| --- | --- | --- | --- | --- | --- |
| 1.52 (0.10,22.34) | LC + ERCP (one-stage) |  |  |  |  |
| 0.92 (0.09,8.93) | 0.60 (0.14,2.53) | LC + ERCP (two-stage) |  |  |  |
| 0.89 (0.01,81.27) | 0.58 (0.01,37.29) | 0.97 (0.02,47.83) | LC + LTCBDE |  |  |
| 1.66 (0.07,41.90) | 1.09 (0.11,11.07) | 1.80 (0.18,17.88) | 1.87 (0.02,172.62) | LC + LCBDE (mixed) |  |
| 0.68 (0.20,2.39) | 0.45 (0.03,7.25) | 0.74 (0.07,8.07) | 0.77 (0.01,74.58) | 0.41 (0.02,11.30) | LC + LCBDE + TTD |

**Table S8.12b:** league table of postoperative intra-abdominal infection in Cohort studies. The columns represent the comparison of the intervention listed in the row to the intervention listed in the column. The rows represent the comparison of the intervention listed in the row to the intervention listed in the column. Effect estimates are expressed as ORs with 95% CIs. For example, the OR for severe postoperative complications for LC + LCBDE + TTD compared with LC + LTCBDE is 0.05 (95% Cl 0.00 to 0.91). An OR greater than 1 indicates higher odds of postoperative surgical site infection in the intervention listed in the row,whereas an OR less than 1 indicates lower odds.

| LC + LCBDE + PS |  |  |  |  |  |
| --- | --- | --- | --- | --- | --- |
| 1.31 (0.01,220.27) | LC + ERCP (one-stage) |  |  |  |  |
| 3.91 (0.05,335.83) | 2.99 (0.24,37.06) | LC + ERCP (two-stage) |  |  |  |
| 10.36 (0.39,273.74) | 7.92 (0.16,403.66) | 2.65 (0.13,54.26) | LC + LTCBDE |  |  |
| 3.53 (0.04,337.89) | 2.70 (0.22,32.33) | 0.90 (0.37,2.20) | 0.34 (0.01,7.94) | LC + LCBDE (mixed) |  |
| 0.47 (0.09,2.35) | 0.36 (0.00,50.72) | 0.12 (0.00,8.47) | 0.05 (0.00,0.91) | 0.13 (0.00,10.42) | LC + LCBDE + TTD |

**8.13 Postoperative cholangitis**

**Table S8.13a:** league table of postoperative cholangitis in RCTs. The columns represent the comparison of the intervention listed in the row to the intervention listed in the column. The rows represent the comparison of the intervention listed in the row to the intervention listed in the column. Effect estimates are expressed as ORs with 95% CIs. For example, the OR for severe postoperative complications for LC + LCBDE + TTD compared with LC + LTCBDE is 0.93 (95% Cl 0.12 to 7.26). An OR greater than 1 indicates higher odds of postoperative cholangitis in the intervention listed in the row, whereas an OR less than 1 indicates lower odds.

| LC + LCBDE + PS |  |  |  |  |  |
| --- | --- | --- | --- | --- | --- |
| 0.24 (0.02,3.40) | LC + ERCP (one-stage) |  |  |  |  |
| 0.37 (0.05,2.57) | 1.52 (0.25,9.16) | LC + ERCP (two-stage) |  |  |  |
| 1.17 (0.79,1.74) | 4.82 (0.34,68.65) | 3.17 (0.45,22.43) | LC + LTCBDE |  |  |
| 1.11 (0.03,46.16) | 4.57 (0.12,177.04) | 3.00 (0.12,72.61) | 0.95 (0.02,39.91) | LC + LCBDE (mixed) |  |
| 1.08 (0.14,8.28) | 4.47 (0.40,50.54) | 2.94 (0.57,15.00) | 0.93 (0.12,7.26) | 0.98 (0.03,35.10) | LC + LCBDE + TTD |

**Table S8.13b:** league table of postoperative cholangitis in Cohort studies. The columns represent the comparison of the intervention listed in the row to the intervention listed in the column. The rows represent the comparison of the intervention listed in the row to the intervention listed in the column. Effect estimates are expressed as ORs with 95% CIs. For example, the OR for severe postoperative complications for LC + LCBDE + TTD compared with LC + LTCBDE is 0.11 (95% Cl 0.00 to 3.13). An OR greater than 1 indicates higher odds of postoperative cholangitis in the intervention listed in the row, whereas an OR less than 1 indicates lower odds.

| LC + LCBDE + PS |  |  |  |  |  |
| --- | --- | --- | --- | --- | --- |
| 0.89 (0.15,5.10) | LC + ERCP (one-stage) |  |  |  |  |
| 0.43 (0.20,0.96) | 0.49 (0.09,2.65) | LC + ERCP (two-stage) |  |  |  |
| 3.59 (0.20,65.27) | 4.05 (0.15,112.51) | 8.27 (0.46,150.20) | LC + LTCBDE |  |  |
| 1.56 (0.52,4.67) | 1.76 (0.29,10.49) | 3.59 (1.65,7.79) | 0.43 (0.02,8.71) | LC + LCBDE (mixed) |  |
| 0.41 (0.07,2.24) | 0.46 (0.04,4.84) | 0.94 (0.17,5.14) | 0.11 (0.00,3.13) | 0.26 (0.04,1.69) | LC + LCBDE + TTD |

**8.14: Symptomatic biliary stricture**

**Table S8.14a:** league table of symptomatic biliary stricture in RCTs. The columns represent the comparison of the intervention listed in the row to the intervention listed in the column. The rows represent the comparison of the intervention listed in the row to the intervention listed in the column. Effect estimates are expressed as ORs with 95% CIs. For example, the OR for severe postoperative complications for LC + LCBDE + TTD compared with LC + ERCP (two-stage) is 0.20 (95% Cl 0.00 to 28.57). An OR greater than 1 indicates higher odds of postoperative cholangitis in the intervention listed in the row, whereas an OR less than 1 indicates lower odds.

| LC + LCBDE + PS |  |  |  |  |
| --- | --- | --- | --- | --- |
| 4.85 (0.08,289.77) | LC + ERCP (two-stage) |  |  |  |
| 4.85 (0.02,1388.49) | 1.00 (0.02,49.82) | LC + LTCBDE |  |  |
| 0.99 (0.06,15.57) | 0.20 (0.01,4.20) | 0.20 (0.00,28.57) | LC + LCBDE + TTD |  |

**Table S8.14b:** league table of symptomatic biliary stricture in Cohort studies. The columns represent the comparison of the intervention listed in the row to the intervention listed in the column. The rows represent the comparison of the intervention listed in the row to the intervention listed in the column. Effect estimates are expressed as ORs with 95% CIs. For example, the OR for severe postoperative complications for LC + LCBDE + TTD compared with LC + LTCBDE is 0.24 (95% Cl 0.00 to 12.72). An OR greater than 1 indicates higher odds of postoperative cholangitis in the intervention listed in the row, whereas an OR less than 1 indicates lower odds.

| LC + LCBDE + PS |  |  |  |  |
| --- | --- | --- | --- | --- |
| 1.77 (0.02,173.48) | LC + ERCP (one-stage) |  |  |  |
| 2.31 (0.20,26.46) | 1.30 (0.03,63.29) | LC + ERCP (two-stage) |  |  |
| 1.72 (0.03,96.00) | 0.97 (0.01,148.59) | 0.75 (0.03,18.23) | LC + LCBDE (mixed) |  |
| 0.41 (0.05,3.01) | 0.23 (0.00,21.87) | 0.18 (0.02,1.92) | 0.24 (0.00,12.72) | LC + LCBDE + TTD |

**8.15: Stone recurrence**

**Table S8.15a:** league table of stone recurrence in RCTs. The columns represent the comparison of the intervention listed in the row to the intervention listed in the column. The rows represent the comparison of the intervention listed in the row to the intervention listed in the column. Effect estimates are expressed as ORs with 95% CIs. For example, the OR for severe postoperative complications for LC + LCBDE + TTD compared with LC + LCBDE + PS is 1.35 (95% Cl 0.19 to 9.83). An OR greater than 1 indicates higher odds of postoperative cholangitis in the intervention listed in the row, whereas an OR less than 1 indicates lower odds.

| LC + LCBDE + PS |  |  |  |  |  |
| --- | --- | --- | --- | --- | --- |
| 1.28 (0.03,51.18) | LC + ERCP (one-stage) |  |  |  |  |
| 0.51 (0.04,6.26) | 0.39 (0.02,6.91) | LC + ERCP (two-stage) |  |  |  |
| 0.49 (0.00,61.76) | 0.38 (0.00,58.18) | 0.97 (0.02,60.24) | LC + LTCBDE |  |  |
| 0.25 (0.01,5.79) | 0.20 (0.01,2.77) | 0.50 (0.06,4.00) | 0.52 (0.01,53.04) | LC + LCBDE (mixed) |  |
| 1.35 (0.19,9.83) | 1.05 (0.03,31.75) | 2.66 (0.33,21.45) | 2.76 (0.03,282.83) | 5.30 (0.32,86.68) | LC + LCBDE + TTD |

**Table S8.15b:** league table of stone recurrence in RCTs. The columns represent the comparison of the intervention listed in the row to the intervention listed in the column. The rows represent the comparison of the intervention listed in the row to the intervention listed in the column. Effect estimates are expressed as ORs with 95% CIs. For example, the OR for severe postoperative complications for LC + LCBDE + TTD compared with LC + LTCBDE is 0.33 (95% Cl 0.07 to 1.65). An OR greater than 1 indicates higher odds of postoperative cholangitis in the intervention listed in the row, whereas an OR less than 1 indicates lower odds.

| LC + LCBDE + PS |  |  |  |  |  |
| --- | --- | --- | --- | --- | --- |
| 0.87 (0.28,2.68) | LC + ERCP (one-stage) |  |  |  |  |
| 0.50 (0.26,0.96) | 0.57 (0.19,1.69) | LC + ERCP (two-stage) |  |  |  |
| 3.39 (0.74,15.50) | 3.88 (0.61,24.66) | 6.79 (1.38,33.55) | LC + LTCBDE |  |  |
| 1.13 (0.46,2.77) | 1.30 (0.52,3.26) | 2.27 (1.13,4.56) | 0.33 (0.06,1.86) | LC + LCBDE (mixed) |  |
| 1.13 (0.52,2.46) | 1.30 (0.37,4.56) | 2.27 (0.99,5.22) | 0.33 (0.07,1.65) | 1.00 (0.35,2.84) | LC + LCBDE + TTD |

# Appendix 9: CINeMA Assessment and Transitivity Assessment

We used the CINeMA framework to assess the certainty of evidence for each network estimate based on the following domains:

**Within study bias:** We classified the overall risk of bias for each study as low risk of bias, the risk of bias as moderate when none of the four assessed risk of bias items were rated as high risk, and the risk of bias as high when one or both items were rated as high risk. See Appendix 4 for the bias assessment. The risk-of-bias assessments for single-session stone clearance rate are shown in Figures S9.1 and S9.2, and those for severe postoperative complications are presented in Figures S9.3 and S9.4.

**Figure S9.1:** Risk of bias contribution by intervention group for Single-session stone clearance rate.

Abbreviations: LC, laparoscopic cholecystectomy; LCBDE, laparoscopic common bile duct exploration; LTCBDE, laparoscopic transcystic bile duct exploration; ERCP, endoscopic retrograde cholangiopancreatography; PS, primary suture;TTD,T-tube drainage.

**Figure S9.2:** Overall risk of bias by treatment comparison in Single-session stone clearance rate.

Abbreviations: LC, laparoscopic cholecystectomy; LCBDE, laparoscopic common bile duct exploration; LTCBDE, laparoscopic transcystic bile duct exploration; ERCP, endoscopic retrograde cholangiopancreatography; PS, primary suture;TTD,T-tube drainage.

**Figure S9.3:** Risk of bias contribution by intervention group for severe postoperative complications.

Abbreviations: LC, laparoscopic cholecystectomy; LCBDE, laparoscopic common bile duct exploration; LTCBDE, laparoscopic transcystic bile duct exploration; ERCP, endoscopic retrograde cholangiopancreatography; PS, primary suture;TTD,T-tube drainage.

**Figure S9.4:** Overall risk of bias by treatment comparison in severe postoperative complications.

Abbreviations: LC, laparoscopic cholecystectomy; LCBDE, laparoscopic common bile duct exploration; LTCBDE, laparoscopic transcystic bile duct exploration; ERCP, endoscopic retrograde cholangiopancreatography; PS, primary suture;TTD,T-tube drainage.

**Reporting bias:** We judged it visually by a funnel plot (Appendix 10).

**Indirectness:** The transitivity assumption was assessed by comparing key clinical effect modifiers across intervention groups, including patient age, stone size, and common bile duct diameter.

**Figure S9.5:** Distribution of mean age, common bile duct diameter, and stone size across intervention groups (RCTs).

**Table S9.1:** Transitivity (Indirectness) Assessment for RCTs

| Intervention | Baseline variable (Mean ± SD) | | |
| --- | --- | --- | --- |
|  | Age (year) | Stone size (cm) | CBD diameter (cm) |
| LC + LCBDE + PS | 53.4 ± 14.6 | 1.28 ± 1.64 | 1.18 ± 0.41 |
| LC + ERCP (one-stage) | 41.2 ± 12.8 | 0.78 ± 0.48 | 0.87 ± 0.26 |
| LC + ERCP (two-stage) | 49.8 ± 15.9 | 0.92 ± 0.54 | 1.02 ± 0.31 |
| LC + LTCBDE | 40.3 ± 8.1 | 1.00 ± 1.00 | 0.86 ± 0.12 |
| LC + LCBDE (mixed) | 40.1 ± 13.9 | 0.73 ± 0.41 | 0.90 ± 0.25 |
| LC + LCBDE + TTD | 52.8 ± 14.3 | 1.14 ± 0.40 | 1.27 ± 0.36 |

Abbreviations: LC, laparoscopic cholecystectomy; LCBDE, laparoscopic common bile duct exploration; LTCBDE, laparoscopic transcystic bile duct exploration; ERCP, endoscopic retrograde cholangiopancreatography; PS, primary suture;TTD,T-tube drainage.

**Figure S9.6:** Distribution of mean age, common bile duct diameter, and stone size across intervention groups (Cohort studies).

**Table S9.2:** Transitivity (Indirectness) Assessment for Cohort studies

| Intervention | Baseline variable (Mean ± SD) | | |
| --- | --- | --- | --- |
|  | Age (year) | Stone size (cm) | CBD diameter (cm) |
| LC + LCBDE + PS | 58.9 ± 15.8 | 1.31 ± 0.69 | 1.25 ± 0.39 |
| LC + ERCP (one-stage) | 56.3 ± 14.0 | 0.84 ± 0.46 | 1.05 ± 0.32 |
| LC + ERCP (two-stage) | 58.6 ± 16.1 | 0.93 ± 0.47 | 1.01 ± 0.34 |
| LC + LTCBDE | 52.4 ± 15.6 | 0.78 ± 0.36 | 0.92 ± 0.29 |
| LC + LCBDE (mixed) | 59.8 ± 16.3 | 0.71 ± 0.38 | 1.13 ± 0.36 |
| LC + LCBDE + TTD | 60.7 ± 14.9 | 1.21 ± 0.81 | 1.33 ± 0.47 |

Abbreviations: LC, laparoscopic cholecystectomy; LCBDE, laparoscopic common bile duct exploration; LTCBDE, laparoscopic transcystic bile duct exploration; ERCP, endoscopic retrograde cholangiopancreatography; PS, primary suture;TTD,T-tube drainage.

**Imprecision:** We use the CINeMA website to grade the accuracy of each comparison.

**Heterogeneity:** We assessed the degree of worry by comparing clinical reasoning based on 95% CIs while applying the same clinical reasoning framework as for inaccuracy. In particular, we judged the consistency of our findings based on the confidence and prediction intervals associated with clinically important effect sizes. And we used the same thresholds of clinical significance as described above and followed the recommendations automatically provided by CINeMA (https://cinema.ispm.unibe.ch/).

**Inconsistency:** For inconsistency, we looked at the results for node splitting (Appendix 5) and we saw major problems when p<0.10, but otherwise no problems.

**Table S9.3:** CINeMA results for single-session stone clearance rate.

| **Comparison** | **Within-study bias** | **Reporting bias** | **Indirectness** | **Imprecision** | **Heterogeneity** | **Incoherence** | **Confidence rating** |
| --- | --- | --- | --- | --- | --- | --- | --- |
| **LC + ERCP (one-stage):LC + ERCP (two-stage)** | Some concerns | Low risk | No concerns | Major concerns | No concerns | No concerns | Low |
| **LC + ERCP (one-stage):LC + LCBDE (mixed)** | No concerns | Low risk | No concerns | Major concerns | No concerns | No concerns | Low |
| **LC + ERCP (two-stage):LC + LCBDE (mixed)** | No concerns | Low risk | No concerns | Major concerns | No concerns | Major concerns | Very low |
| **LC + ERCP (two-stage):LC + LCBDE + PS** | Some concerns | Low risk | No concerns | Major concerns | No concerns | No concerns | Low |
| **LC + ERCP (two-stage):LC + LCBDE + TTD** | Some concerns | Low risk | No concerns | Major concerns | No concerns | No concerns | Low |
| **LC + ERCP (two-stage):LC + LTCBDE** | Some concerns | Low risk | No concerns | Major concerns | No concerns | No concerns | Low |
| **LC + LCBDE + PS:LC + LCBDE + TTD** | Some concerns | Low risk | No concerns | Major concerns | No concerns | No concerns | Low |
| **LC + ERCP (one-stage):LC + LCBDE + PS** | Some concerns | Low risk | No concerns | Major concerns | No concerns | No concerns | Low |
| **LC + ERCP (one-stage):LC + LCBDE + TTD** | Some concerns | Low risk | No concerns | Major concerns | No concerns | No concerns | Low |
| **LC + ERCP (one-stage):LC + LTCBDE** | Some concerns | Low risk | No concerns | Major concerns | No concerns | No concerns | Low |
| **LC + LCBDE (mixed):LC + LCBDE + PS** | Some concerns | Low risk | No concerns | Major concerns | No concerns | No concerns | Low |
| **LC + LCBDE (mixed):LC + LCBDE + TTD** | Some concerns | Low risk | No concerns | Major concerns | No concerns | No concerns | Low |
| **LC + LCBDE (mixed):LC + LTCBDE** | Some concerns | Low risk | No concerns | Major concerns | No concerns | No concerns | Low |
| **LC + LCBDE + PS:LC + LTCBDE** | Some concerns | Low risk | No concerns | Major concerns | No concerns | No concerns | Low |
| **LC + LCBDE + TTD:LC + LTCBDE** | Some concerns | Low risk | No concerns | Major concerns | No concerns | No concerns | Low |

Abbreviations: LC, laparoscopic cholecystectomy; LCBDE, laparoscopic common bile duct exploration; LTCBDE, laparoscopic transcystic bile duct exploration; ERCP, endoscopic retrograde cholangiopancreatography; PS, primary suture;TTD,T-tube drainage.

**Table S9.3:** CINeMA results for severe postoperative complications.

| **Comparison** | **Within-study bias** | **Reporting bias** | **Indirectness** | **Imprecision** | **Heterogeneity** | **Incoherence** | **Confidence rating** |
| --- | --- | --- | --- | --- | --- | --- | --- |
| LC + ERCP (one-stage) VS LC + ERCP (two-stage) | Some concerns | Low risk | No concerns | Major concerns | No concerns | No concerns | Low |
| LC + ERCP (one-stage) VS LC + LCBDE (mixed) | No concerns | Low risk | No concerns | Major concerns | No concerns | No concerns | Low |
| LC + ERCP (two-stage) VS LC + LCBDE (mixed) | No concerns | Low risk | No concerns | Major concerns | No concerns | No concerns | Low |
| LC + ERCP (two-stage) VS LC + LCBDE + PS | Some concerns | Low risk | No concerns | No concerns | Major concerns | Major concerns | Very low |
| LC + ERCP (two-stage) VS LC + LCBDE + TTD | Some concerns | Low risk | No concerns | Major concerns | No concerns | No concerns | Low |
| LC + ERCP (two-stage) VS LC + LTCBDE | Some concerns | Low risk | No concerns | Major concerns | No concerns | No concerns | Low |
| LC + LCBDE + PS VS LC + LCBDE + TTD | Some concerns | Low risk | No concerns | Major concerns | No concerns | No concerns | Low |
| LC + LCBDE + PS VS LC + LTCBDE | Some concerns | Low risk | No concerns | Major concerns | No concerns | No concerns | Low |
| LC + ERCP (one-stage) VS LC + LCBDE + PS | Some concerns | Low risk | No concerns | Major concerns | No concerns | No concerns | Low |
| LC + ERCP (one-stage) VS LC + LCBDE + TTD | Some concerns | Low risk | No concerns | Major concerns | No concerns | No concerns | Low |
| LC + ERCP (one-stage) VS LC + LTCBDE | Some concerns | Low risk | No concerns | Major concerns | No concerns | No concerns | Low |
| LC + LCBDE (mixed) VS LC + LCBDE + PS | Some concerns | Low risk | No concerns | Major concerns | No concerns | No concerns | Low |
| LC + LCBDE (mixed) VS LC + LCBDE + TTD | Some concerns | Low risk | No concerns | Major concerns | No concerns | No concerns | Low |
| LC + LCBDE (mixed) VS LC + LTCBDE | Some concerns | Low risk | No concerns | Major concerns | No concerns | No concerns | Low |
| LC + LCBDE + TTD VS LC + LTCBDE | Some concerns | Low risk | No concerns | Major concerns | No concerns | No concerns | Low |

Abbreviations: LC, laparoscopic cholecystectomy; LCBDE, laparoscopic common bile duct exploration; LTCBDE, laparoscopic transcystic bile duct exploration; ERCP, endoscopic retrograde cholangiopancreatography; PS, primary suture;TTD,T-tube drainage.

# Appendix 10: Funnel plots

The figures show the assessment of small study effect bias in studies on the effects of different minimally invasive strategies for the management of gallbladder stones with concomitant common bile duct stones. The funnel plots pertain to all trials included in the network meta-analysis, in which at least one T-tube–free minimally invasive strategy was compared with laparoscopic common bile duct exploration with T-tube drainage or other alternative interventions.In the case of multi-arm trials that compared more than two interventions within the same study, each relevant pairwise comparison was included in the funnel plot analysis.Egger’s regression test was used to assess the symmetry of the funnel plots.When the P value was greater than 0.05, the funnel plot was considered to be symmetrical, indicating no evidence of small-study effects or publication bias. When the P value was less than 0.05, trim-and-fill analysis using the “metatrim” method was planned to evaluate the robustness of the pooled estimates.

**10.1 Single-session stone clearance rate**

**Figure S10.1a:** Funnel plot of single-session stone clearance rate in (RCTs only) .

**Figure S10.1b:** Funnel plot of single-session stone clearance rate (Cohort studies only) .

**10.2 Severe postoperative complications**

**Figure S10.2a:** Funnel plot of severe postoperative complications (RCTs only) .

**Figure S10.2b:** Funnel plot of severe postoperative complications (Cohort studies only) .

**10.3 Operative time**

**Figure S10.3a:** Funnel plot of Operative time (RCTs only) .

**Figure S10.3b:** Funnel plot of Operative time (Cohort studies only) .

**10.4 Total length of hospital stay**

**Figure S10.4a:** Funnel plot of total length of hospital stay (RCTs only) .

**Figure S10.4b:** Funnel plot of total length of hospital stay (Cohort studies only) .

**10.5 Intraoperative blood loss**

**Figure S10.5:** Funnel plot of Intraoperative blood loss (Cohort studies only) .

**10.6 Conversion to open surgery**

**Figure S10.6a:** Funnel plot of conversion to open surgery (RCTs only) .

**Figure S10.6b:** Funnel plot of conversion to open surgery (Cohort studies only) .

**10.7 Reintervention**

**Figure S10.7a:** Funnel plot of reintervention (RCTs only) .

**Figure S10.7b:** Funnel plot of reintervention (Cohort studies only) .

**10.8 Postoperative bile leakage.**

**Figure S10.8a:** Funnel plot of postoperative bile leakage (RCTs only) .

**Figure S10.8b:** Funnel plot of postoperative bile leakage (Cohort studies only) .

**10.9 Postoperative pancreatitis.**

**Figure S10.9a:** Funnel plot of postoperative pancreatitis (RCTs only) .

**Figure S10.9b:** Funnel plot of postoperative pancreatitis (Cohort studies only) .

**10.10 Postoperative biliary bleeding**

**Figure S10.10a:** Funnel plot of postoperative biliary bleeding (RCTs only) .

**Figure S10.10b:** Funnel plot of postoperative biliary bleeding (Cohort studies only) .

**10.11 Postoperative surgical site infection**

**Figure S10.11a:** Funnel plot of postoperative SSI (RCTs only) .

**Figure S10.11b:** Funnel plot of postoperative SSI (Cohort studies only) .

**10.12 Postoperative intra-abdominal infection**

**Figure S10.12a:** Funnel plot of postoperative intra-abdominal infection (RCTs only) .

**Figure S10.12b:** Funnel plot of postoperative intra-abdominal infection (Cohort studies only) .

**10.13 Postoperative cholangitis**

**Figure S10.13a:** Funnel plot of postoperative cholangitis (RCTs only) .

**Figure S10.13b:** Funnel plot of postoperative cholangitis (Cohort studies only) .

**10.14 Symptomatic biliary stricture**

**Figure S10.14a:** Funnel plot of symptomatic biliary stricture (RCTs only) .

**Figure S10.14b:** Funnel plot of symptomatic biliary stricture (Cohort studies only) .

**10.15 Stone recurrence**

**Figure S10.15a:** Funnel plot of Stone recurrence (RCTs only) .

**Figure S10.15b:** Funnel plot of Stone recurrence (Cohort studies only) .

# Appendix 11: Sensitivity analyses

Sensitivity analyses were performed to assess the impact of excluding the mixed intervention group (LC + LCBDE mixed) on the results of the network meta-analysis.

**Figure S11.1:** Flow diagram of sensitivity analyses

.

**11.2 Sensitivity analyses of primary outcomes**

Effect estimates are expressed as risk ratios (RRs) for single-session stone clearance rate and odds ratios (ORs) for severe postoperative complications. LC + LCBDE + TTD was used as the reference treatment for all comparisons. Sensitivity analyses were performed by excluding the mixed intervention node (LC + LCBDE [mixed]) from the network meta-analysis.

**Table S11.2a:** RCT-based sensitivity analysis

| Intervention | Single-session stone clearance rate(RR) | | Severe postoperative complications(OR) | |
| --- | --- | --- | --- | --- |
|  | Main estimate | Sensitivity analyses | Main estimate | Sensitivity analyses |
| LC + LCBDE + PS | 1.01 (0.99,1.04) | 1.01 (0.94,1.09) | 0.43 (0.16,1.14) | 0.43 (0.16,1.14) |
| LC + ERCP  (one-stage) | 0.99 (0.94,1.05) | 0.95 (0.83,1.09) | 0.27 (0.05,1.48) | 0.42 (0.07,2.52) |
| LC + ERCP  (two-stage) | 0.97 (0.92,1.01) | 0.93 (0.83,1.03) | 0.81 (0.21,3.16) | 0.78 (0.20,3.04) |
| LC + LTCBDE | 1.00 (0.92,1.08) | 0.96 (0.81,1.13) | 0.78 (0.01,50.35) | 0.75 (0.01,48.39) |

Abbreviations: LC, laparoscopic cholecystectomy; LCBDE, laparoscopic common bile duct exploration; LTCBDE, laparoscopic transcystic bile duct exploration; ERCP, endoscopic retrograde cholangiopancreatography; PS, primary suture.

**Table S11.2b:**Cohort-based sensitivity analysis

| Intervention | Single-session stone clearance rate(RR) | | Severe postoperative complications(OR) | |
| --- | --- | --- | --- | --- |
|  | Main estimate | Sensitivity analyses | Main estimate | Sensitivity analyses |
| LC + LCBDE + PS | 1.01 (0.97,1.05) | 1.01 (0.96,1.05) | 1.55 (0.49,4.96) | 1.59 (0.47,5.35) |
| LC + ERCP  (one-stage) | 1.03 (0.96,1.10) | 1.03 (0.94,1.12) | 1.30 (0.26,6.46) | 3.17 (0.16,62.75) |
| LC + ERCP  (two-stage) | 0.96 (0.92,1.01) | 0.96 (0.91,1.01) | 1.40 (0.53,3.71) | 1.44 (0.51,4.05) |
| LC + LTCBDE | 1.00 (0.94,1.06) | 1.00 (0.94,1.07) | 1.15 (0.24,5.55) | 1.16 (0.22,6.20) |

Abbreviations: LC, laparoscopic cholecystectomy; LCBDE, laparoscopic common bile duct exploration; LTCBDE, laparoscopic transcystic bile duct exploration; ERCP, endoscopic retrograde cholangiopancreatography; PS, primary suture.
